# Decreased expression of ATF3, orchestrated by β-catenin/TCF3, miR-17-5p and HOXA11-AS, promoted gastric cancer progression via increased β-catenin and CEMIP

**DOI:** 10.1038/s12276-021-00694-9

**Published:** 2021-11-02

**Authors:** Guohua Xie, Ping Dong, Hui Chen, Ling Xu, Yi Liu, Yanhui Ma, Yingxia Zheng, Junyao Yang, Yunlan Zhou, Lei Chen, Lisong Shen

**Affiliations:** 1 Department of Clinical Laboratory, Xinhua Hospital, Shanghai Jiao Tong University School of Medicine, Shanghai, China; 2 Department of General Surgery, Xinhua Hospital, Shanghai Jiao Tong University School of Medicine, Shanghai, China; 3 Faculty of Medical Laboratory Sciences, Ruijin Hospital, Shanghai Jiao Tong University School of Medicine, Shanghai, China

**Keywords:** Gastric cancer, Experimental models of disease

## Abstract

ATF3 has been reported to be dysregulated in various cancers and involved in various steps of tumorigenesis. However, the mechanisms underlying the abnormal expression of ATF3 and its biological function in gastric cancer (GC) have not been well investigated. Here, we report ATF3 as one of the key regulators of GC development and progression. Patients with low ATF3 expression had shorter survival and a poorer prognosis. In vitro and in vivo assays investigating ATF3 alterations revealed a complex integrated phenotype that affects cell growth and migration. Strikingly, high-throughput sequencing and microarray analysis of cells with ATF3 silencing or of ATF3-low GC tissues indicated alterations in the Wnt signaling pathway, focal adhesions and adherens junctions. Mechanistically, the expression of β-catenin and cell migration inducing hyaluronidase 1 (CEMIP) was significantly upregulated in GC cells with downregulated ATF3, which was synergistically repressed by the β-catenin/TCF3 signaling axis and noncoding RNA miR-17-5p and HOXA11-AS. In addition, we found that WDR5 expression was promoted by TCF3 and is involved in miR-17-5p and HOXA11-AS activation in GC cells. Taken together, our findings revealed the mechanism of ATF3 downregulation and its biological role in regulating the expression of Wnt signaling-related genes during GC progression, suggesting new informative biomarkers of malignancy and therapeutic directions for GC patients.

## Gastric cancer: Possible treatment targets identified

New treatments for gastric cancer could involve controlling the activity of a regulatory gene and associated signaling pathway. Over-activation of the Wnt signaling pathway, which regulates many cellular functions, occurs in around half of gastric cancers. Further, the activating transcription factor 3 gene (*ATF3*) is thought to influence tumorigenesis, although its role in gastric cancer is unclear. Guohua Xie and co-workers at Shanghai Jiao Tong University, China, explored the function of *ATF3* in human gastric cancer tissues. Patients with low *ATF3* expression had poorer prognosis and shorter life expectancy. The team discovered that reduced expression of *ATF3* triggered the increased expression of two of its target genes, which then altered Wnt signaling. Reduced *ATF3* expression also boosted the invasiveness of gastric cancer cells. Initial results suggest that overexpression of *ATF3* could suppress gastric cancer progression.

## Introduction

Gastric cancer (GC) is one of the most common malignancies in the world and is characterized by a high frequency of metastasis and low 5-year survival rate^1,2^. The pathogenesis of GC is multifactorial and includes *Helicobacter pylori* infection, Epstein-Barr virus, dietary pattern, alcohol consumption and sodium intake^3,4^. Despite advances in diagnostic and therapeutic strategies, the clinical outcomes and prognosis of GC patients (GCs) remain pessimistic. More than one million new cases are diagnosed annually, and more than 700,000 GCs die every year worldwide^5,6^. Thus, a better understanding of the mechanisms underlying GC pathogenesis is essential to the development of effective and novel therapies.

The canonical Wnt signaling pathway, in addition to its role in early embryogenesis and immune system development, plays a key role in regulating many cell functions, including proliferation, differentiation, stem cell maintenance, and homeostasis^7–10^. In the absence of Wnt ligands, β-catenin is targeted for ubiquitin/proteasome-mediated degradation by a destruction complex composed of the tumor suppressor adenomatous polyposis coli, glycogen synthase kinase-3β, axin, and calcium kinase-1. Wnt signaling suppresses the function of this complex and induces the association of β-catenin with T-cell factor/lymphoid enhancing factor, thus activating the transcription of a variety of Wnt target genes involved in tumorigenesis^7,8,10–12^. Studies have reported that canonical Wnt signaling is dysregulated in many kinds of cancers, including GC^12^, hepatocellular carcinoma (HCC)^11^ and colorectal cancer (CRC)^13^. Abnormally activated canonical Wnt/β-catenin signaling was observed in more than 30–50% of GCs^7,14^, and a large body of evidence implicates aberrant activation of Wnt signaling in virtually all stages of tumorigenesis, from malignant transformation to metastatic dissemination and resistance to treatment^8–10,12,14^.

Activating transcription factor 3 (ATF3), a stress-inducible gene that encodes a basic leucine zipper transcription factor, belongs to the ATF/CREB subfamily^15^. In response to cellular stressors, ATF3 regulates diverse cellular functions by binding to the ATF/CREB cis-regulatory element or interacting with other proteins^15–18^. There is growing evidence that ATF3 is involved in several important cellular signaling pathways, including those mediated by Toll-like receptor 4^15^, actin-modifying protein^18^, transforming growth factor-β (TGF-β)^19^ and TP53^20^. Previously, we demonstrated that ATF3 directly induced the transcription of gelsolin concomitant with alterations in the actin cytoskeleton and was functionally involved with metastasis of bladder cancer^18^. ATF3 can also interact with the NF-κB network to regulate the expression of cytokines, thereby participating in the cellular immune response^15^. Although recent studies have revealed that ATF3 is involved in several human diseases, including sepsis-associated immunosuppression^21^, nonalcoholic fatty liver disease^22^ and colitis^23^, the biological role of ATF3 in cancer is obscure. ATF3 has been shown to suppress tumor growth and metastasis in many other cancer types, such as colon cancer^13^, bladder cancer^18^, glioblastoma^19^ and lung cancer^24^. However, ATF3 can promote the proliferation of human HCC^25^ and prostate cancer^26^. Moreover, recent reports showed that ATF3 expressed in stromal cells and noncancer host cells promoted breast cancer cell dissemination into the lung and chemotherapy-exacerbated breast cancer metastasis, respectively^27,28^. Therefore, ATF3 is involved in the regulation of tumorigenesis, and its potential function depends on the cancer types and cell-specific context. However, whether and how ATF3 regulates GC development and progression has not been explored. In addition, the underlying molecular mechanisms involved in the regulation of ATF3 expression, particularly the regulation of noncoding RNAs, are poorly characterized.

Herein, a series of in vitro and in vivo experiments demonstrated that (1) ATF3 expression was significantly reduced in GC tissues and is associated with the survival of GCs; (2) the expression level of ATF3 affected GC cell proliferation and metastasis; (3) ATF3 was involved in Wnt/β-catenin signaling in GC, and decreased ATF3 significantly promoted the upregulation of CEMIP expression; (4) the expression of miR-17-5p and HOXA11-AS was significantly increased in ATF3^low^ GC tissues and contributed to ATF3 repression through direct and indirect regulatory mechanisms; and (5) remarkably elevated levels of miR-17-5p and HOXA11-AS were promoted by WDR5, which was transactivated by abnormally elevated levels of TCF3 in GC cells.

## Materials and methods

### Patients and specimens

Samples of fresh tumor tissues and adjacent normal tissues were gathered from 46 GCs who underwent curative surgery from 2016 to 2017 at Xinhua Hospital Affiliated to Shanghai Jiao Tong University School of Medicine. Written informed consent was obtained from all patients. The tissues were stored at −80 °C and subjected to mRNA extraction for reverse transcription quantitative real-time PCR (RT-qPCR). One commercial tissue microarray (TMA, catalog no. HStmA180Su09, named TMA180Su09) was purchased from Shanghai Outdo Biotech Company. This TMA contained 180 tissue spots comprising 90 pairs of GC tissues and the corresponding adjacent noncancerous mucosa tissues. The clinicopathological features and survival data of the patients were provided by the manufacturer. This TMA was used to detect ATF3 expression by immunohistochemistry (IHC) staining and evaluated its relationship with clinicopathological factors and clinical outcomes. Another two TMAs without survival data (G10 comprising 35 pairs of GC tissues and matching normal tissues; GC1-a comprising tumor tissues from 54 GCs) were purchased from Wuhan Servicebio Technology. TMA G10 was used to detect TCF3 and ATF3 expression in consecutive GC tissue sections by IHC. The TMA GC1-a was used to detect the expression levels of miR-17-5p and ATF3 in consecutive GC tissue sections by fluorescence in situ hybridization (FISH) and IHC, respectively. Informed consent was signed by all the patients and possessed by the TMA manufacturer. This study was approved by the Ethics Committee of Xinhua Hospital (XHEC-D-2020-036).

### Cell culture and reagents

The human GC cell lines AGS, BGC-823, HGC-27, KATO III, MGC-803, MKN-45, SGC-7901, SNU-1, and NCI-N87 and the human embryonic kidney cell line HEK293T were purchased from the Cell Bank of the Chinese Academy of Sciences (Shanghai, China). All cell lines were routinely maintained in high-glucose Dulbecco’s modified Eagle’s medium (DMEM, HyClone, USA) supplemented with 10% fetal bovine serum (FBS, Gibco, USA) at 37 °C under 5% CO_2_ in an incubator. XAV-939 (S1180, Selleck, USA), Lipofectamine^TM^ 3000 Transfection Reagent (L3000015, Thermo Fisher, USA), riboFECT^TM^ CP Reagent (C10511-1, Ribobio, China), Attractene Transfection Reagent (Cat No. 301005, QIAGEN, Germany), TRIzol^TM^ LS Reagent (Cat No. 10296-028, Thermo Fisher, USA), miRNA mimics (YM00471966-ADA) and inhibitors (YI04100215-DDA, QIAGEN, Germany), the lncRNA FISH probe (lnc1100261, Ribobio, China), a Fluorescent In Situ Hybridization Kit (C10910, Ribobio, China), and h-U6 FISH Probe Mix (lnc110101, Ribobio, China) were used in this study.

### RNA extraction and RT-qPCR

Total RNA was extracted from cells and tissues using TRIzol LS reagent (Thermo Fisher, USA). RNA was reverse transcribed into cDNA, and cDNA was used as a template for amplification with specific primers (Supplementary Table 1). U6 and β-actin were used as miRNA and mRNA internal controls, respectively. Experiments were performed according to the manufacturer’s instructions (TaKaRa, Japan).

### Western blot analysis and antibodies

Western blots were performed as described^18^ with primary antibodies targeting ATF3 (1:1000, #33593, Cell Signaling, USA), CEMIP (1:5000, 45750002, Novus Biological, USA), β-catenin (1:1000, #8480, Cell Signaling, USA), TCF3 (1:1000, #12258, Cell Signaling, USA) and WDR5 (1:1000, #13105, Cell Signaling, USA). Antibody targeting β-actin (A5441, 1:5000, Sigma Aldrich, USA) was used as a loading control for all blots. Images were viewed with an Odyssey imaging system (LI-COR Biosciences, Lincoln, NE, USA).

### Immunohistochemistry

TMA tissue sections were deparaffinized, and antigen retrieval was performed in citrate buffer for 3 min at 100 °C. Endogenous peroxidase activity and nonspecific antigens were blocked with peroxidase blocking reagent followed by incubation with primary antibodies against ATF3 (1:300, HPA001562; Sigma–Aldrich, USA) and TCF3 (1:200, sc-133075, Santa Cruz, USA) overnight at 4 °C. After washing, sections were incubated with biotin-labeled secondary antibody and subsequently incubated with a streptavidin-peroxidase complex. The peroxidase reaction was developed using 3,3′-diaminobenzidine (DAB) chromogen solution in DAB buffer substrate (#8059, Cell Signaling, USA). Sections were visualized with DAB, counterstained with hematoxylin, mounted in neutral gum and analyzed using a brightfield microscope. The IHC score was calculated by multiplying the staining intensity grade (grade of 0, 1, 2, or 3 indicating negative, weak-positive, moderate-positive or strong-positive, respectively) by the positive rate score (score of 0, 1, 2, 3, or 4 indicating positive areas of 0–5%, 6–25%, 26–50%, 51–75%, or 76–100%, respectively).

### RNA interference

Chemically modified small interfering RNAs (siRNAs) targeting CEMIP (SIGS0012573-1), β-catenin (SIGS0002532-1), TCF3 (SIGS0002560-1), WDR5 (SIGS0009833-1), h-HOXA11-AS Smart Silencer (lnc3161111110630), lncRNA Smart Silencer NC (lnc3N0000001-1-5), and negative control RNAs (siCont, siN0000001-1-5) were purchased from RiboBio Company (Guangzhou, China). The sequences of the above siRNAs are listed in Supplementary Table 2. RiboFECT™ CP Reagent (RiboBio, Guangzhou, China) was used to transfect siRNAs according to the manufacturer’s instructions.

### Lentivirus production and infection

Lentiviral particles carrying hsa-miR-17-5p inhibitor, the human ATF3 coding sequence, ATF3 short hairpin RNA (shATF3-1, −2) and their respective controls (shCtrl) were constructed by GeneChem, Shanghai, China (Supplementary Table 2). MGC-803, MKN-45, AGS, and HGC-27 cells were infected with lentiviral particles, and the expression of ATF3 was measured using RT-qPCR and western blot.

### Cell proliferation and colony formation

For the proliferation assay, GC cells were seeded in 96-well plates at a density of 2000 cells/well for 6 days with six duplicates per condition. For the colony formation assay, GC cells were seeded in 6-well culture plates at 200 cells/well. After incubation for 14 days, cells were washed twice with Hank’s solution and stained with hematoxylin solution. The number of colonies containing ≥ 50 cells was counted under a microscope. For the soft agar colony formation assay, cells were seeded in 6-well plates in growth medium containing 0.35% agar on top of a layer of growth medium containing 0.5% agar. Growth medium supplemented with 10% fetal bovine serum was added on top of the agar. After incubation for 14–21 days, colonies were fixed with 4% paraformaldehyde, stained with 0.05% crystal violet in 25% methanol, and counted under a microscope. All experiments were repeated at least three times.

### In vitro cell migration and invasion assays

In vitro cell migration and invasion assays were performed according to a previous description^18^. All assays were independently repeated at least three times.

### In vivo tumorigenesis and metastasis assays

For xenograft experiments, ATF3-overexpressing or ATF3-knockdown GC cells (2 × 10^6^ cells/mouse, 0.2 ml) were subcutaneously inoculated into the flanks of 4- to 6-week-old female BALB/c-nu/nu mice (*n* = 5 per group). Mice were maintained in a barrier facility on HEPA-filtered racks and fed an autoclaved laboratory rodent diet. All animal studies were conducted in accordance with the principles and procedures outlined in the Institutional Animal Care and Use Committee of Xinhua Hospital (XHEC-F-2020-007). Mice were killed at the indicated time, and tumors were excised, weighed, and processed for histology.

For assessment of peritoneal carcinomatosis, stably infected AGS and MGC-803 cells (2 × 10^6^) were implanted into the abdominal cavity of nude mice via intraperitoneal injection. Mice were monitored for five weeks and sacrificed; animals were evaluated for the presence of ascites, and tumor nodules were counted.

In vivo metastasis assays were performed according to our previous study^18^. A total of 2 × 10^6^ cells were injected into the lateral tail vein of nude mice (*n* = 5 per group). Forty days later, all mice were sacrificed, and lungs were collected for evaluation of metastatic foci and standard histopathology.

### RNA sequencing

Total RNA was isolated by TRIzol LS reagent (Thermo Fisher, USA) and purified using an RNeasy Mini Kit (Qiagen, Germany). The concentration and integrity of total RNA were estimated using a Qubit 2.0 Fluorometer (Thermo Fisher, USA) and an Agilent 2100 Bioanalyzer (Agilent Technologies, USA), respectively. Then, library construction and RNA sequencing (RNA-seq) were performed at LC-Bio Technologies Corporation (Hangzhou, China) with an Illumina NovaSeq 6000 (Illumina, USA), followed by computational analysis. The criteria for differentially expressed genes were defined as a *P* value < 0.05 and fold change > 2.0 or fold change < −2.0.

### Chromatin immunoprecipitation

Chromatin immunoprecipitation (ChIP) assays were performed using the SimpleChIP® Kit (#9003, Cell Signaling, USA). Cells were cross linked with 1% formaldehyde for 10 minutes at room temperature and quenched in glycine solution. DNA was immunoprecipitated from the micrococcal nuclease-digested cell lysates and quantified using SYBR Green in an ABI 7900HT Fast Real-Time PCR System (Applied Biosystems, USA). Primer sequences are listed in Supplementary Table 3. Fold enrichment was calculated based on the 2^−Δ(ΔCt)^ method, where Δ Ct = Ct_IP_ − Ct_Input_ and Δ (ΔCt) = Δ Ct_antibody_ − Δ Ct_IgG_.

### Luciferase reporter assay

The binding motif of the gene promoters and the miRNA binding site in the ATF3 3′UTR were identified by JASPAR (http://jaspar.genereg.net/), TargetScan (http://www.targetscan.org/) or miRTarBase (http://mirtarbase.mbc.nctu.edu.tw). The different promoters or 3′UTR fragments were synthesized and inserted into the expression vector, and all recombinant vectors were verified by sequencing. HEK293T cells were plated in 96-well plates and cotransfected with various plasmids as indicated in the figures. Cells were collected 24–48 h post transfection, and luciferase activities were analyzed by the dual-luciferase reporter assay system (Cat. # E1910, Promega, USA). Reporter activity was normalized to that of Renilla luciferase.

### Fluorescence in situ hybridization

GC cells were fixed in 4% formaldehyde for 15 min and washed with PBS. Fixed cells were treated with pepsin and sequentially dehydrated in 70%, 90%, and 100% ethanol. The air-dried cells were further incubated with a fluorescence in situ hybridization (FISH) probe in hybridization buffer. Hybridization was performed at 55 °C for 2 hours, and the slide was washed and dehydrated. For double FISH, primary and secondary antibodies were added. Finally, the air-dried slide was mounted with Prolong Gold Antifade Reagent with DAPI (#8961 S, Cell Signaling, USA). The sequence of the miR-17-5p probe was 5′-CTACCTGCACTGTAAGCACTTTG-3′ and was synthesized by Servicebio (Wuhan, China). The FISH assay kit and the probe for lncRNA HOXA11-AS were purchased from RiboBio (C10910, lnc1100261 and lnc110101, Guangzhou, China).

### Expression profile of miRNAs and lncRNAs

The mRNA expression levels of ATF3 in GC tissues were analyzed by RT-qPCR. Four GC tissues with relatively lower ATF3 expression compared with that in matched normal tissues were selected for the subsequent miRNA, mRNA and lncRNA ChIP assay according to standard Agilent protocols (Agilent Human miRNA (8 × 60 K) V21.0 and SBC-ceRNA (human 4 × 180 K)). Bioinformatics analysis and visualization of the microarray data were performed with the MeV v4.4 program (http://www.tm4.org/mev/). The criteria for differentially expressed genes were defined as a *P* value < 0.05 and fold change > 2.0 or fold change < −2.0.

### Expression date and bioinformatics analysis

Four public GC microarray gene profiling datasets (GSE27342, GSE66229, GSE61687 and GSE35809) were downloaded from the Gene Expression Omnibus (GEO) on the NCBI web server. The expression data of normal stomach tissue and stomach tumor tissues were obtained from The Cancer Genome Atlas (TCGA). Bioinformatics was used to predict the possibility of interaction of HOXA11-AS with the histone methylation modifiers EZH2 and WDR5 and the transcription factor TCF3. Predictions with probabilities > 0.5 were considered positive. RPISeq predictions were based on random forest or support vector machine (online URL: http://pridb.gdcb.iastate.edu/RPISeq/references.php).

### RNA immunoprecipitation

The RNA immunoprecipitation assay (RIP) was performed with the Magna RIP RNA-binding protein immunoprecipitation kit (Millipore, USA). Briefly, cells were lysed in RNA immunoprecipitation lysis buffer and immunoprecipitated with either the indicated antibody or IgG with protein magnetic beads. After proteinase K digestion, the precipitated nucleotides were purified by phenol chloroform extraction. RNA was then resuspended in RNase-free water, and complementary DNA was synthesized and subjected to RT-qPCR to measure the corresponding mRNA levels of HOXA11-AS or β-actin (internal control).

### In vitro transcription and RNA pull-down assay

In vitro transcription assays were performed using a MEGAscript kit (Life Technologies, USA) according to the manufacturer’s instructions. HOXA11-AS RNAs were labeled via desthiobiotinylation using the Pierce RNA 3′ End Desthiobiotinylation Kit (Thermo Fisher, USA). Then, RNA pull-down assays were performed using the Magnetic RNA-Protein Pull-Down Kit (Thermo Fisher, USA) according to the manufacturer’s instructions.

### Statistical analysis

Statistical analyses were performed with SPSS 16.0 (SPSS, Inc., Chicago, IL, USA). Data are expressed as the means ± s.d. from at least three independent experiments. One-way analysis of variance (ANOVA) or two-tailed Student’s t test was applied to compare quantitative data, while the nonparametric χ2 test was used to analyze qualitative data. Survival analysis was performed using the Kaplan–Meier method. The Cox proportional hazard regression model was employed for univariate or multivariate analysis to determine independent prognostic factors. The level of statistical significance was defined as *p* < 0.05 (*p < 0.05, **p < 0.01 and ***p < 0.001).

## Results

### Downregulated ATF3 expression correlated with poor prognosis of GCs

**Fig. 1:**
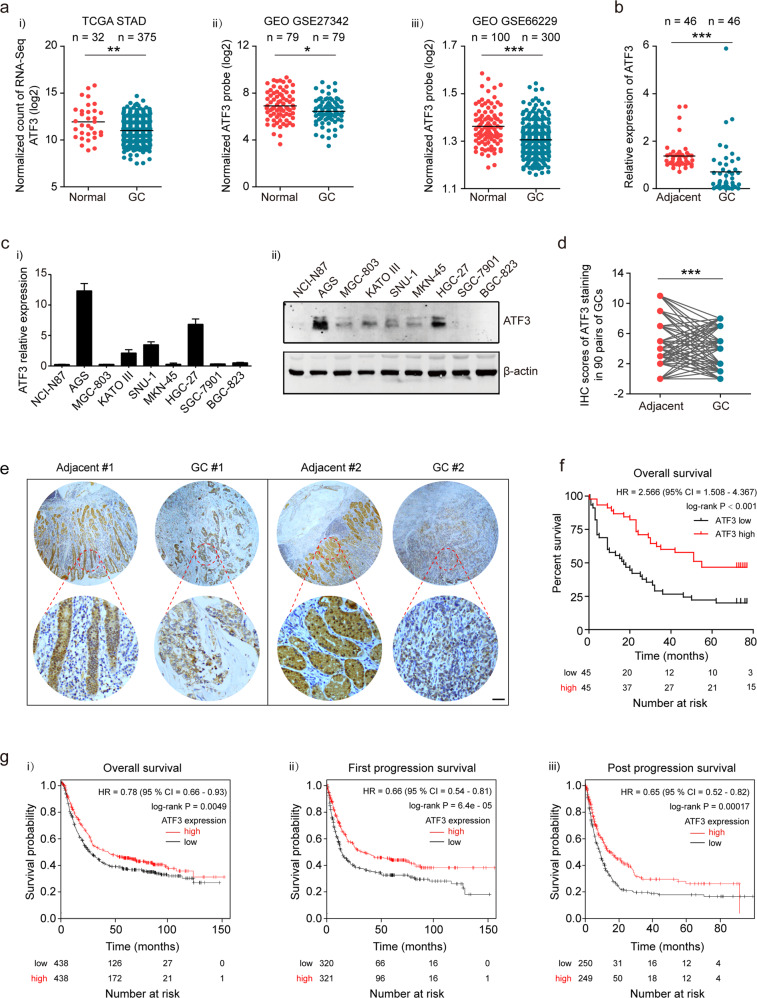
Downregulation of ATF3 expression in GC and its prognostic significance. **a** ATF3 expression data as measured by RNA-seq or a normalized probe were retrieved from The Cancer Genome Atlas (TCGA STAD, i) and Gene Expression Omnibus (GEO, GSE27342-ii, GSE66229-iii) databases, respectively, and were used for comparison between GC samples and normal tissues. The data are presented as a scatter plot. **b** ATF3 mRNA expression was compared between 46 fresh GC samples and their paired adjacent tissues. **c** The expression of ATF3 mRNA (i) and protein (ii) was detected by RT-qPCR and western blot, respectively, in nine GC cell lines. **d** IHC scores of 90 pairs of GC tissues in the TMA180Su09 dataset based on ATF3 expression analyzed by IHC staining. **e** Representative IHC staining results of ATF3 in two human GC tissues and their paired normal tissues (scale bar, 20 μm). **f** Kaplan–Meier (K-M) analysis of overall survival (OS) of 90 GC patients according to the ATF3 IHC scores (data from the TMA180Su09 dataset). **g** i) K–M plot of OS of 876 GCs; ii) K–M plot of first progression survival (FPS) of 641 GCs; iii) K–M plot of post progression survival (PPS) of 499 GCs. The data were obtained from an online dataset from K–M Plotter with 202672_s_as the probe, and GC patients were stratified by the median ATF3 expression (*p* = 0.0049 for OS; *p* = 6.4e-05 for FP; *p* = 0.0002 for PPS; log-rank test). *p < 0.05, **p < 0.01 and ***p < 0.001 by Student’s *t* test compared with the normal group.

Previous studies reported that ATF3 expression was frequently downregulated in several tumor types. To explore the role of ATF3 in GCs, we examined ATF3 expression in 375 GC samples and 32 normal tissues using RNA-seq data deposited in the TCGA database. Consistent with previous reports in other tumor types, ATF3 expression was significantly lower in GC tissues than in normal tissues (*p* = 0.0076, Fig. 1a-i). Further comparison of ATF3 expression between paired GC tissues and normal tissues in two reports from the GEO datasets also showed significantly decreased ATF3 expression in GCs (*p* = 0.0126 for GSE27342, *p* < 0.001 for GSE66229, Fig. 1a-ii, -iii). To confirm the RNA sequencing or normalized probe results obtained from the database, we examined 46 paired GC and normal tissue samples by RT-qPCR analysis, and ATF3 mRNA was conspicuously lower in the tumor tissues (71.7%, 33 of 46 pairs, *p* = 0.0003, Fig. 1b). Cytological experiments revealed similar data, RT-qPCR and western blot assays showed that low ATF3 expression in most of the GC cell lines (Fig. 1c).

We next explored the correlation between ATF3 expression and the clinicopathological factors of 90 GCs in the TMA180Su09 dataset. The IHC staining intensity of ATF3 was remarkably downregulated in GC samples compared with paired adjacent tissues (Fig. 1d, e). Furthermore, we divided the GC samples into high and low ATF3 expression groups according to the median IHC score of ATF3 expression. The chi-square test was performed to evaluate differences in the clinicopathological factors between the two groups. As shown in Supplementary Table 4, ATF3 expression was correlated with tumor size (*p* = 0.006), serosal invasion (*p* = 0.014), peritoneal dissemination (*p* = 0.049) and tumor invasion depth (*p* = 0.038). Other clinical factors, such as patient sex and age, histological grade, lymphatic invasion, lymph node metastasis and TNM stage, were not found to be significantly associated with ATF3 expression in this study.

To determine the relationship between ATF3 expression and GC prognosis, we attempted to evaluate the correlation between ATF3 expression and clinical outcomes. K–M analysis and log-rank tests were used to evaluate the effects of ATF3 expression and clinicopathological characteristics on OS. The median survival time for the high ATF3 expression group was 30 ± 4.743 months, while that for the low ATF3 expression group was only 17 ± 4.695 months. As shown in Fig. 1f, downregulation of ATF3 predicted a poor prognosis in GCs (log-rank *p* < 0.001). In addition, the survival data for GCs registered in the K-M Plotter online database (202672_s_at probe, http://kmplot.com/analysis/index.php?p=service&cancer=gastric) were analyzed and revealed that low ATF3 expression was significantly associated with poor OS (log-rank *p* = 0.0049), FPS (log-rank *p* = 6.4e-05) and PPS (log-rank *p* = 0.00017) in GCs (Fig. 1g).

To further confirm the prognostic role of ATF3 in GCs, univariate and multivariate survival analyses were performed. Univariate analysis identified eight prognostic factors: tumor size, histological grade, serosal invasion, peritoneal dissemination, tumor invasion depth, lymph node metastasis, TNM stage and ATF3 expression. Multivariate analysis further revealed that ATF3 expression could be regarded as an independent predictor for OS in GCs (*p* = 0.032), as well as tumor size (*p* = 0.005) (Supplementary Table 5). Taken together, our data confirmed that ATF3 is frequently downregulated in GCs and is closely related to poor prognosis.

### Downregulated ATF3 facilitated the proliferation and tumorigenic ability of GC cells in vitro and in vivo

**Fig. 2:**
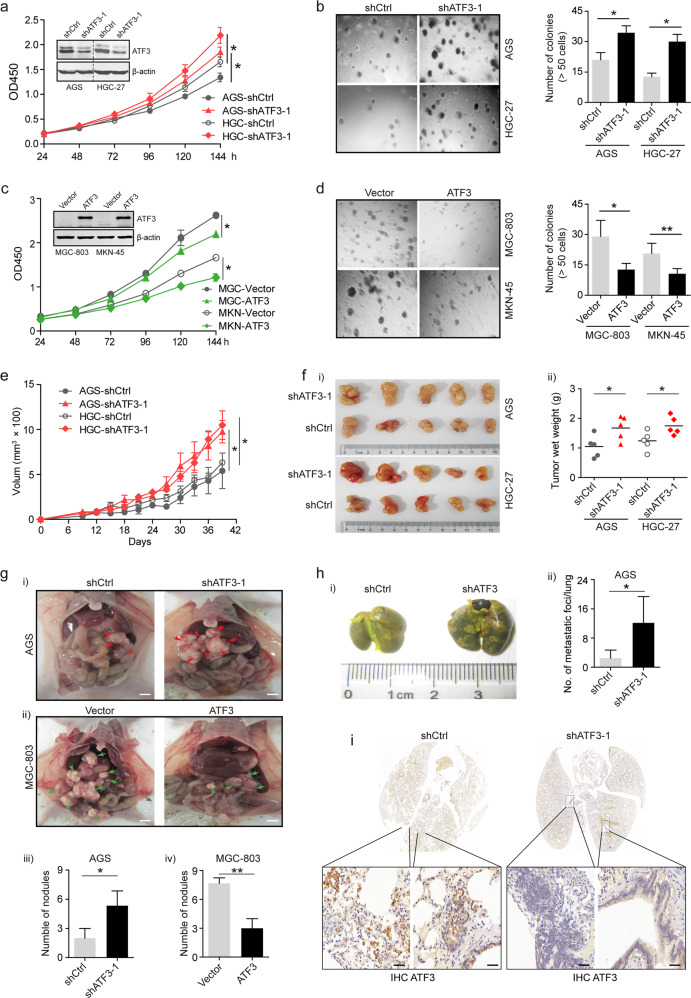
ATF3 exerts a tumor suppressive function in GC cells in vitro and in vivo. **a**, **b** shATF3 lentivirus or control lentivirus was individually transduced into AGS and HGC-27 cells, respectively. The knockdown efficiency was validated, and the proliferation capacities were detected by CCK-8 (**a**) and colony formation assays in soft agar (**b**), with bar charts showing the colony numbers. The results are expressed as the mean ± s.d. of 3 independent experiments. **c**, **d** ATF3 lentivirus or control was individually transduced into MGC-803 and MKN-45 cells. The overexpression efficiency was confirmed, and the proliferation capacities were detected by CCK-8 (**c**) and colony formation assays in soft agar (**d**), with bar charts showing the colony numbers. The results are expressed as the mean ± s.d. of 3 independent experiments. **e**, **f** Tumor growth curves (**e**) of ATF3-knockdown AGS and HGC-27 cells (or negative control cells) in the xenograft mouse model based on tumor size. Moreover, tumor nodules (**f**-**i**) were collected, and tumor weights (**f**-**ii**) were recorded to highlight the growth difference under the influence of ATF3 knockdown. The values shown are the mean ± s.d. of five tumors. **g** ATF3 affected peritoneal spreading in nude mice. Representative images of nude mice intraperitoneally injected with ATF3-knockdown AGS cells (i) or ATF3-overexpressing MGC-803 cells (ii). Control lentivirus-infected cells were used as the corresponding control. Quantification of the peritoneal nodules is shown in the bar graph (iii, iv; lower panels). Data are presented as the mean ± s.d. of five mice, Scale bar: 5 mm. **h** ATF3-knockdown AGS cells or control cells were injected into the tail vein of nude mice (*n* = 5). Representative images of lung metastasis models eight weeks after tail vein injection are shown (i), and the number of metastatic lung nodules was counted (ii). Data are presented as the mean ± s.d. of five mice. **i** Histological analysis showed ATF3 expression levels in metastatic GC cells in lung tissue sections from the experimental and control groups. Scale bar: 20 μm. *p < 0.05; **p < 0.01 by Student’s *t* test compared with *t*he control group.

To investigate the biological implications of ATF3 in GC, based on the mRNA and protein expression levels of ATF3 among 9 GC cell lines (Supplementary Fig. 1a–e), we used lentiviral transduction to knock down ATF3 expression in AGS and HGC-27 cells and overexpress ATF3 in MGC-803 and MKN-45 cells. CCK-8 and anchorage-dependent and anchorage-independent colony formation assays indicated that ATF3 deficiency promoted the proliferative ability of AGS and HGC-27 cells (Fig. 2a, b and Supplementary Fig. 1f). Conversely, upregulated ATF3 had the opposite effect in MGC-803 and MKN-45 cells (Fig. 2c, d and Supplementary Fig. 1g). To confirm the role of ATF3 in vivo, tumor xenograft models were constructed, and ATF3 depletion enhanced tumorigenesis with prominently higher tumor volumes and weights than those in the negative control group (Fig. 2e, f). Moreover, overexpression of ATF3 caused an inverse phenotype in xenograft mice (Supplementary Fig. 1h–j). In summary, we believe that downregulating ATF3 is beneficial for the proliferation and tumorigenesis of GC cells.

### Downregulated ATF3 facilitated GC cell motility and invasiveness in vitro and in vivo

As several studies reported that ATF3 was significantly associated with tumor metastasis^18,27,29,30^, the effect of ATF3 on GC cell migratory and invasive capabilities was further investigated by performing transwell assays. The results showed that knockdown of ATF3 in AGS cells resulted in a significant increase in cell migration and invasion (Supplementary Fig. 1k-i, -iii and 1l-i, -iii). However, upregulation of ATF3 in MGC-803 cells elicited the opposite effect (Supplementary Fig. 1k-ii, -iv and 1l-ii, -iv). Next, we assessed the impact of ATF3 on tumor metastasis in vivo. Tumor peritoneal metastasis models were established and revealed that the number of peritoneal nodules in the ATF3-knockdown AGS group was significantly greater than that in the control group (Fig. 2g-i, -iii). Moreover, ectopic expression of ATF3 in MGC-803 cells caused an inverse phenotype (Fig. 2g-ii, -iv), implying that reduced ATF3 expression enhanced the metastatic ability of GC cells in vivo. Furthermore, ATF3-knockdown AGS cells and control cells were injected via tail vein into nude mice to establish lung metastasis models. Compared with the control group, the ATF3-knockdown AGS group was associated with considerably more lung metastatic nodules (Fig. 2h-i, -ii) and more metastatic foci in lung tissue sections. The ATF3-knockdown AGS group formed more irregular luminal structures in the mouse lung than did the control group (Fig. 2i), indicating that the metastatic colonization potential of GC cells could be facilitated by inhibiting ATF3 expression. Our in vivo data therefore complemented the results of the functional in vitro studies on ATF3.

### β-catenin and CEMIP are direct targets of ATF3 and contribute to the proliferation and metastasis of GC cells with low ATF3 expression

**Fig. 3:**
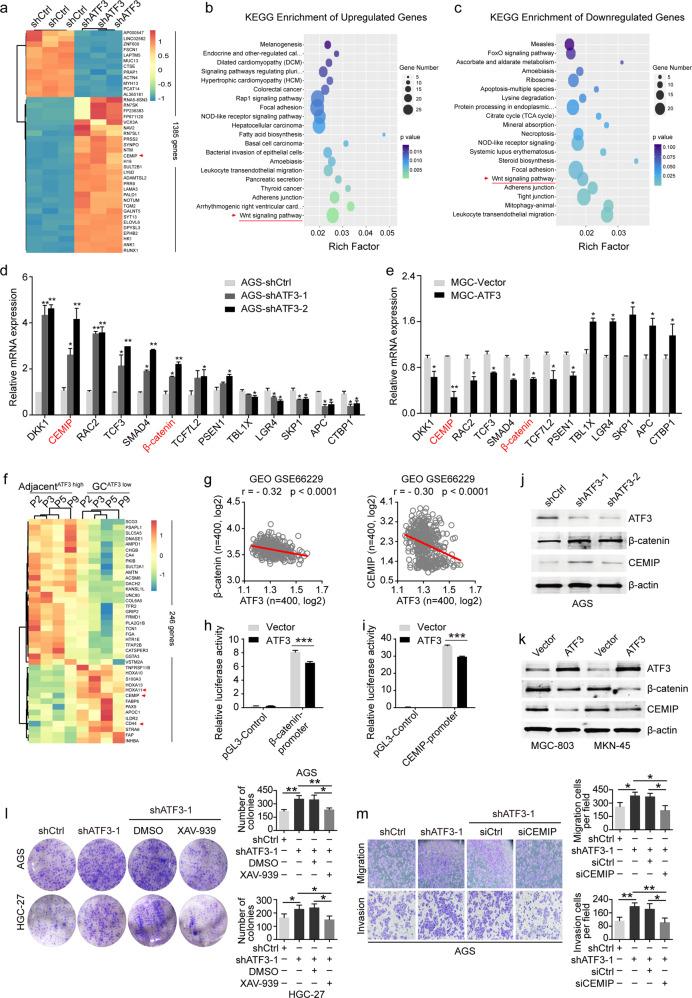
β-catenin and CEMIP are direct targets of ATF3 in GC cells, and decreased ATF3 expression promoted the expression of β-catenin and CEMIP. **a** Mean-centered hierarchical clustering of 776 transcripts that were altered (≥ 2-fold change, *p* < 0.05) in shATF3 lentivirus-treated cells (shATF3) compared to control lentivirus-treated cells (shCtrl), with three repeats. **b**, **c** KEGG analysis of altered transcripts with upregulated or downregulated expression. **d**, **e** The mRNA levels of the identified genes were selectively confirmed by RT-qPCR in GC cells with ATF3 knockdown (**d**) or ATF3 overexpression (e). **f** Hierarchical clustering of 264 transcripts with altered expression (≥2-fold or ≤ −2-fold changes, *p* < 0.05) in ATF3^low^ GC tissues compared with paired adjacent ATF3^high^ normal tissues. **g** Correlation between the mRNA levels of ATF3 and those of β-catenin (i) or CEMIP in the GEO GSE66229 dataset. **h**, **i** Promoter activity of β-catenin (**h**) and CEMIP (i) as measured by luciferase reporter assays in HEK293 cells cotransfected with the indicated promoter regions upstream of luciferase and either ATF3 or empty vector, respectively. The data are presented as the mean ± s.d. **j** Western blot analysis of ATF3, β-catenin and CEMIP expression in ATF3-knockdown AGS cells and control cells. **k** Western blot analysis of ATF3, β-catenin and CEMIP expression in ATF3-overexpressing BGC-803 and MKN-45 cells. **l** Colony formation assay in ATF3-knockdown AGS and HGC-27 cells upon β-catenin inhibition. **m** Transwell migration and Matrigel invasion assays were performed to determine the effect of CEMIP inhibition on the migration and invasion of ATF3-knockdown AGS cells. The data are presented as the mean ± s.d., *n* = 3. *p < 0.05; **p < 0.01, ***p < 0.001 and n.s. (not significant) by Student’s *t* test compared with the control group.

To identify potential ATF3-associated pathways and target genes that could mediate GC progression on an unbiased basis, we performed RNA transcriptome sequencing and assessed the gene expression profile of AGS cells that were suppressed by ATF3. A common set of 776 mRNAs showed ≥ 2-fold increased abundance, and silencing ATF3 also reduced the abundance (≤−2-fold) of 609 genes (Fig. 3a and Supplementary Table 6). Gene ontology (GO) analysis indicated that the significantly enriched biological processes included pathways related to signal transduction, regulation of transcription, cell adhesion and cell proliferation, and cell apoptosis (Supplementary Fig. 2a). Kyoto Encyclopedia of Genes and Genomes (KEGG) pathway analysis of the top twenty significantly enriched pathways showed that the upregulated and downregulated genes mainly belonged to the “Wnt signaling pathway”, “focal adhesion” and “adherens junction” (Fig. 3b, c). These genes included many well-known Wnt signaling-related genes (e.g., DKK1, CEMIP, RAC2, TCF3, SMAD4, β-catenin, TCF7L2, PSEN1, TBL1X, LGR4, SKP1, APC and CTBP1). The expression changes of these genes were selectively confirmed by RT-qPCR (Fig. 3d, e). To further explore and confirm the role of ATF3, 4 GC tissues with low ATF3 expression (ATF3^low^) compared to paired adjacent tissues (ATF3^high^) were selected (Supplementary Fig. 2b), and the mRNA expression profiles were analyzed (Fig. 3f). A common set of 87 mRNAs showed ≥ 2-fold increased abundance, and 177 genes also showed significantly reduced abundance (≤ −2-fold, Fig. 3f and Supplementary Table 7). RT-qPCR analysis indicated that many Wnt pathway-related genes were also severely dysregulated in ATF3^low^ GC tissues compared with control tissues, which was consistent with the RNA-sequencing analysis of the ATF3-knockdown AGS cells (Supplementary Fig. 2c).

ATF3 is a transcription factor whose key molecular function is DNA binding and transcriptional regulation (Supplementary Fig. 2a). We next analyzed whether the expression level of any of these aberrantly expressed Wnt signaling-related genes was negatively correlated with ATF3 expression in GC. To achieve this, we examined gene expression levels in publicly available transcriptomic profiling datasets and in our sample set. The data showed that β-catenin and CEMIP were significantly upregulated in the TCGA STAD and GSE66229 datasets as well as in our sample cohort (Supplementary Fig. 3a-i, a-ii, b-i, b-ii, c-i, c-ii, d-i, d-ii and c). In addition, a significant negative correlation between ATF3 and β-catenin or CEMIP expression was detected in both the TCGA STAD and GSE66229 datasets (Fig. 3g and Supplementary Fig. 3d). We next investigated whether β-catenin and CEMIP are direct transcriptional targets of ATF3. Available ATF3 ChIP-seq data (Cistrome Data Browser, http://cistrome.org) showed that ATF3 binds to several regions of the β-catenin and CEMIP promoters in a variety of human cells (Supplementary Fig. 3e, f). The luciferase reporter assay was used to further study the functional effect of ATF3 binding to the β-catenin and CEMIP promoters. The data indicated that ATF3 significantly inhibited the luciferase activity of both the β-catenin and CEMIP promoters (Fig. 3h, i). Furthermore, increased expression of β-catenin and CEMIP was detected by western blot in ATF3-knockdown AGS cells (Fig. 3j). Consistently, significantly reduced protein expression of β-catenin and CEMIP was also detected in ATF3-overexpressing MGC-803 and MKN-45 cells (Fig. 3k).

β-catenin and CEMIP are Wnt-related proteins, and a recent study reported that CEMIP is present in high levels in metastatic tumor-derived exosomes and promotes tumor metastasis by creating a metastatic environment^31^. To determine whether the suppression of β-catenin is sufficient to affect the proliferation ability of GC cells, β-catenin was specifically inhibited in ATF3-knockdown AGS cells through the specific β-catenin inhibitor XAV-939 (100 μM, Supplementary Fig. 3g), and the colony formation assay showed that ATF3 knockdown in AGS cells enhanced their colony-forming ability, an effect that was impaired upon β-catenin depletion (Fig. 3l). Next, to investigate whether CEMIP was involved in the enhanced migration and invasion capacity observed upon ATF3 knockdown, CEMIP expression was inhibited by specific siRNAs in ATF3-inhibited AGS cells. The knockdown efficiency of the siRNA targeting CEMIP was validated (Supplementary Fig. 3h). Depletion of CEMIP significantly reduced the migration and invasion capability of ATF3-inhibited AGS cells (Fig. 3m). Therefore, the proliferation, migration, and invasion capacity caused by downregulated ATF3 depends, at least in part, on its direct downstream target genes β-catenin and CEMIP.

### The β-catenin/TCF3 signaling axis suppressed ATF3 expression in GC

**Fig. 4:**
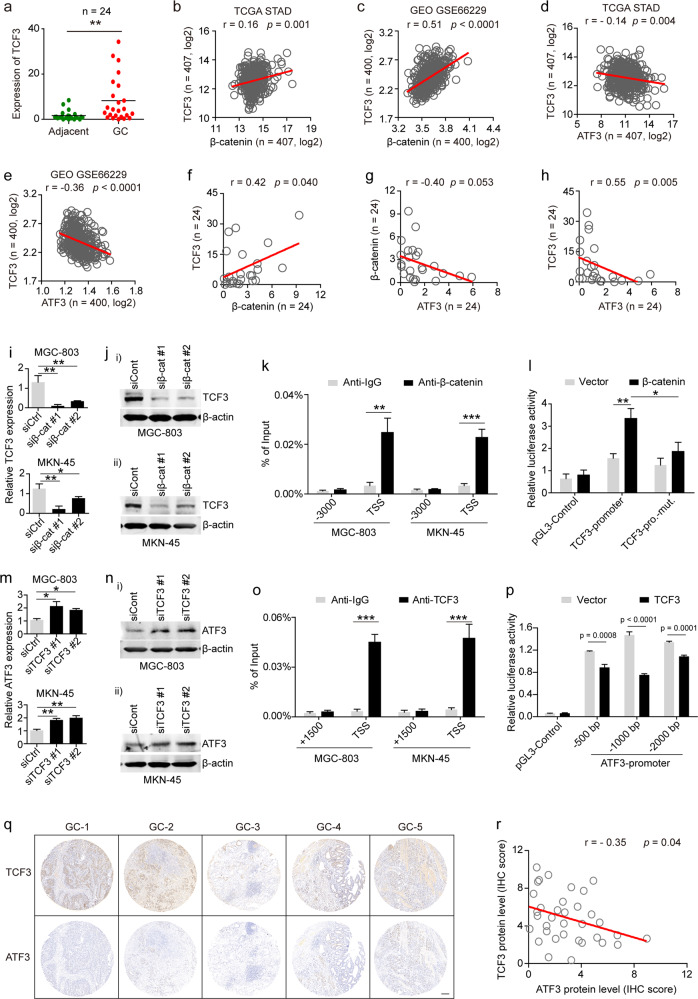
The β-catenin-TCF3 axis repressed ATF3 expression in GC cells. **a** RT-qPCR analysis of TCF3 mRNA expression in our GC sample set. **b**, **c** Correlation between the mRNA levels of β-catenin and TCF3 in the TCGA STAD specimens and the GEO GSE66229 dataset. **d**, **e** Correlations between the mRNA levels of TCF3 and ATF3 in the TCGA STAD specimens (**d**) and the GEO GSE66229 dataset (e). **f** Correlation between the mRNA levels of β-catenin and TCF3 in our sample set. **g**, **h** Correlations between the mRNA levels of ATF3 and β-catenin (**g**) or TCF3 (h) in our sample set. **i**, **j** qPCR and western blot analysis of TCF3 mRNA (**i**) and protein expression (**j**-**i**, -**ii**), respectively, in β-catenin-knockdown BGC-803 and MKN-45 cells. **k** The ChIP assay was performed to detect β-catenin occupancy in the TCF3 promoter region, and the IgG group was used as a negative control. **l** TCF3 promoter activity was measured by luciferase reporter assays in HEK293 cells cotransfected with β-catenin or empty vector. Data are presented as the mean ± s.d. *n* = 3. **m**, **n** RT-qPCR and western blot analysis of ATF3 mRNA (**m)** and protein expression (**n**-**i**, -**ii**), respectively, in TCF3-knockdown MGC-803 and MKN-45 cells. **o** ChIP assays were performed to detect TCF3 occupancy in the ATF3 promoter region, and the IgG group was used as a negative control. **p** ATF3 promoter activity was measured by luciferase reporter assays in HEK293 cells cotransfected with TCF3 or empty vector. The data are presented as the mean ± s.d. **q** The protein expression levels of TCF3 and ATF3 were detected in consecutive paraffin sections of tissues from the GC TMA G10 database by IHC staining. **r** Correlation between the IHC scores of ATF3 and TCF3 in the GC TMA G10 specimens as detected by IHC staining. *p < 0.05, **p < 0.01 and ***p < 0.001 by Student’s t test compared with the paired normal or control group.

To explore the role of the β-catenin/TCF3 signaling axis in ATF3 expression in GC cells, we first examined TCF3 expression and the expression correlation between ATF3 and β-catenin or TCF3 in the online database and our samples. We found that TCF3 was significantly upregulated in GC samples (*p* < 0.001 for our samples, *p* < 0.0001 for STAD TCGA and GSE66229) (Fig. 4a and Supplementary Fig. 4a-i, -ii). The expression of β-catenin was positively associated with TCF3 (Fig. 4b, c). In addition, the expression of β-catenin and TCF3 was negatively correlated with ATF3 expression (Fig. 4d, e and Supplementary Fig. 4b-i, -ii), and the expression correlation analysis of β-catenin, TCF3 and ATF3 in our GC samples was consistent with the data retrieved from the online database (Fig. 4f–h).

To investigate whether ATF3 was regulated by β-catenin and TCF3 in GC cells, the expression of β-catenin and TCF3 was knocked down in MGC-803 and MKN-45 cells by infection with a specific shRNA lentivirus (Supplementary Fig. 4c-i, -ii and 4d-i, -ii). We showed that knockdown of β-catenin or TCF3 resulted in increased ATF3 mRNA and protein levels in both MGC-803 and MKN-45 cells (Fig. 4i, j-i, j-ii, m, n-i, n-ii and Supplementary Fig. 4e-i, -ii). ChIP and luciferase reporter assays confirmed that β-catenin could directly bind to the TCF3 promoter (Fig. 4k) and promote TCF3 transcription (Fig. 4l). ChIP and luciferase assays also revealed that TCF3 could directly bind to the ATF3 promoter and repress ATF3 transcription (Fig. 4o, p). Our data were confirmed by the data derived from the GSE61687 dataset, in which ATF3 mRNA expression was increased while TCF3 and CEMIP mRNA was reduced upon β-catenin knockdown in AGS cells (Supplementary Fig. 4f-i, -ii, -iii, -iv).

Furthermore, the expression of TCF3 and ATF3 in clinical tissues was analyzed by using TMA G10. Representative IHC images of four GC tissues showed the opposite expression of TCF3 and ATF3 as that in consecutive IHC sections of normal tissues (Fig. 4q). The IHC score statistics showed that the IHC staining of TCF3 in GC tissues was significantly negatively correlated with the IHC staining of ATF3 (Fig. 4r). Taken together, our data demonstrated that the activated β-catenin/TCF3 axis in GC suppressed ATF3 expression.

### MiR-17-5p is specifically increased in GC tissues and inhibits ATF3

**Fig. 5:**
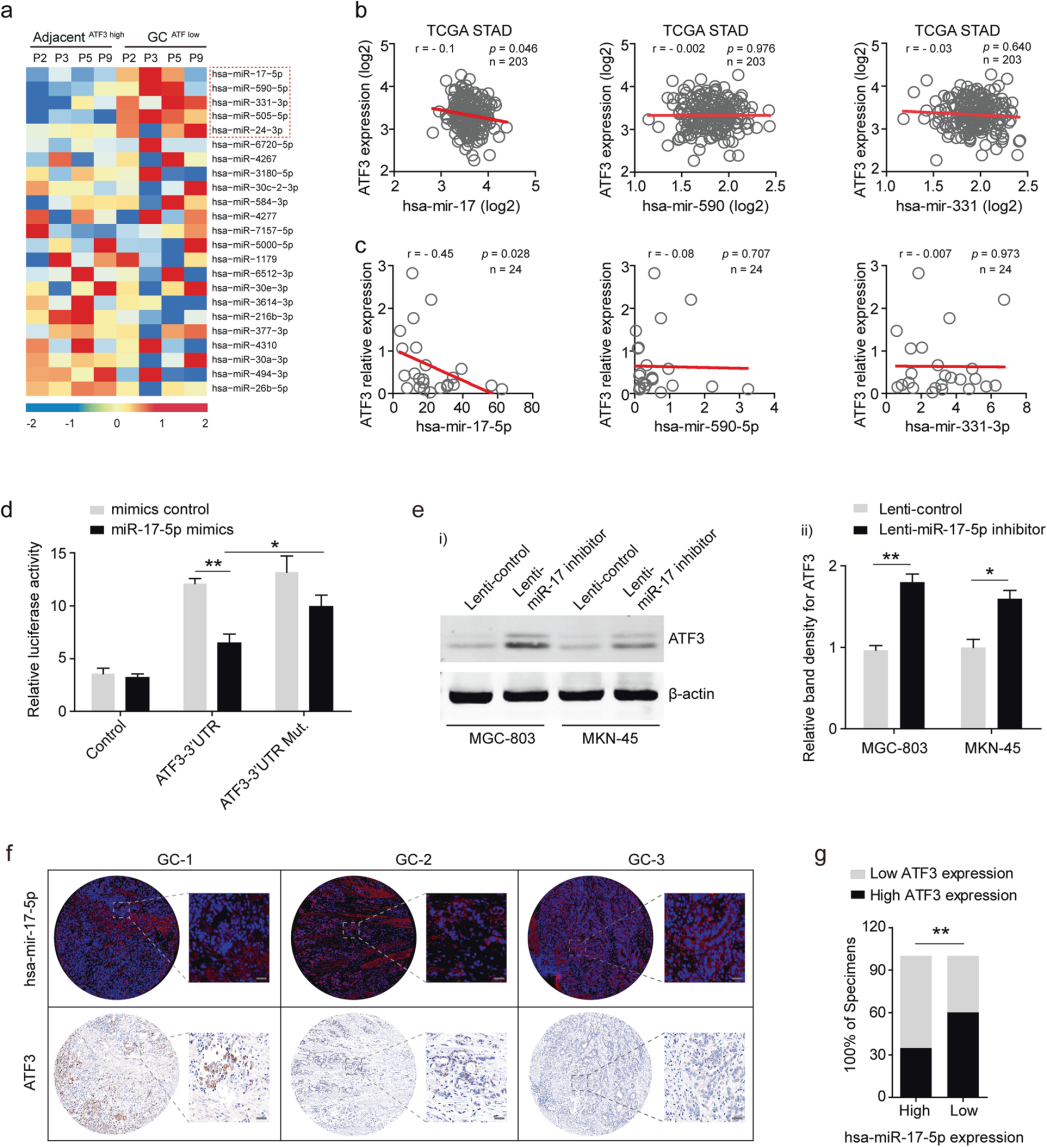
Increased miR-17-5p inhibited ATF3 transcription in GC cells. **a** Heatmap of altered ATF3-targeted microRNAs in ATF3^low^ GC tissues compared with paired adjacent ATF3^high^ normal tissues. The red dotted frame shows specifically increased ATF3-targeted microRNAs detected in ATF3^low^ GC tissues. **b** Correlations between the expression levels of ATF3 and miR-17, miR-590 or miR-331 in TCGA STAD samples. **c** Correlations between the expression levels of ATF3 and miR-17-5p, miR-590-5p or miR-331-3p in our GC samples. **d** A luciferase reporter assay was used to determine the interaction between miRNA and the specific binding site on the ATF3 3′UTR. HEK293 cells were cotransfected with an ATF3 3′UTR construct and either miR-17-5p mimics or control mimics. Luciferase activity is shown as relative luciferase activity normalized to that of Renilla luciferase. Data are expressed as the mean ± s.d. of 3 independent transfections. **e** Western blot analysis of ATF3 levels in MGC-803 and MKN-45 cells infected individually with miR-17-5p-inhibitor lentivirus or control lentivirus. β-actin served as an endogenous control (**e**-**i**). Densitometry analysis of protein bands was performed with ImageJ, and the normalized ATF3 band intensity in control lentivirus-infected GC cells was set as 1 (**e**-**ii**). **f** Detection of miR-17-5p and ATF3 expression in consecutive paraffin sections of GC tissues from the TMA GC1-a dataset by fluorescence in situ hybridization (FISH) and IHC staining, respectively. Representative images of samples from three GC patients are shown. FISH and histological analysis showed the fluorescence of the miR-17-5p probe and ATF3 IHC staining in the same tissue site in consecutive sections of GC tissues; scale bar: 20 μm. **g** The expression level of ATF3 in GC tissues with high or low expression of miR-17-5p was statistically analyzed. *p < 0.05 and **p < 0.01 by Student’s *t* test compared with the corresponding control group.

miRNAs are a group of short noncoding RNAs that can mediate posttranscriptional gene silencing. To investigate whether any microRNAs play a suppressive role in ATF3 transcription in GC, microRNA expression profiles of four ATF3^low^ GC specimens were analyzed by microRNA microarray, and the expression of five microRNAs specifically targeted to ATF3 was upregulated significantly (Fig. 5a). To avoid the detection bias owing to the small sample size, we analyzed the expression of these five microRNAs in the large cohort from the TCGA STAD dataset (*n* = 491) and found that the expression of hsa-miR-17, hsa-miR-590 and hsa-miR-505 were significantly increased in STAD samples compared to normal tissues (Supplementary Fig. 5a-i, -ii, -iii, -iv, -v, -vi). Among the five candidate miRNAs, only the hsa-miR-17 probe could be retrieved from the GSE66229 dataset (*n* = 400), which showed that hsa-miR-17 was significantly increased in GC tissues (Supplementary Fig. 5b). The correlation between ATF3 and the 5 specifically elevated microRNAs was further analyzed with the TCGA STAD specimens, which showed that only hsa-miR-17 expression was negatively correlated with ATF3 expression (Fig. 5b and Supplementary Fig. 5c-i, -ii, -iii). In addition, the correlation analysis of ATF3 and hsa-miR-17 was validated by the data derived from the GSE66229 dataset (Supplementary Fig. 5d). Assessment of our GC samples was consistent with the data derived from the above online database (Fig. 5c). The ATF3 3′UTR harbors miR-17-5p-interacting sequences (1382–1407 nt) that are highly conserved in mammals (Supplementary Fig. 5e). To validate the predicted miRNA-target interaction, HEK293T cells were transfected with miR-17-5p mimics (100 nM), which showed reduced (~40%) luciferase activity when cotransfected with plasmids containing the ATF3 3′UTR but not with control mimics or the mutant ATF3 3′UTR (Fig. 5d). Furthermore, the functional consequence of this miRNA-target interaction in GC cells was measured, and western blot assays showed that miR-17-5p inhibition significantly increased ATF3 protein expression in MGC-803 and MKN-45 cells (Fig. 5e-i, -ii). To further confirm the correlation of miR-17-5p and its specific target ATF3, miR-17-5p and ATF3 were detected in the TMA GC1-a by FISH and IHC staining, respectively, and the data showed that miR-17-5p expression was negatively associated with ATF3 expression (Fig. 5f). Statistics indicated that GC cells with higher miR-17-5p levels had relatively lower levels of ATF3 (as shown in the enlarged representative images of GC-2 and GC-3 tissues, Fig. 5g).

### Increased HOXA11-AS in GC cells inhibited ATF3 transcription

**Fig. 6:**
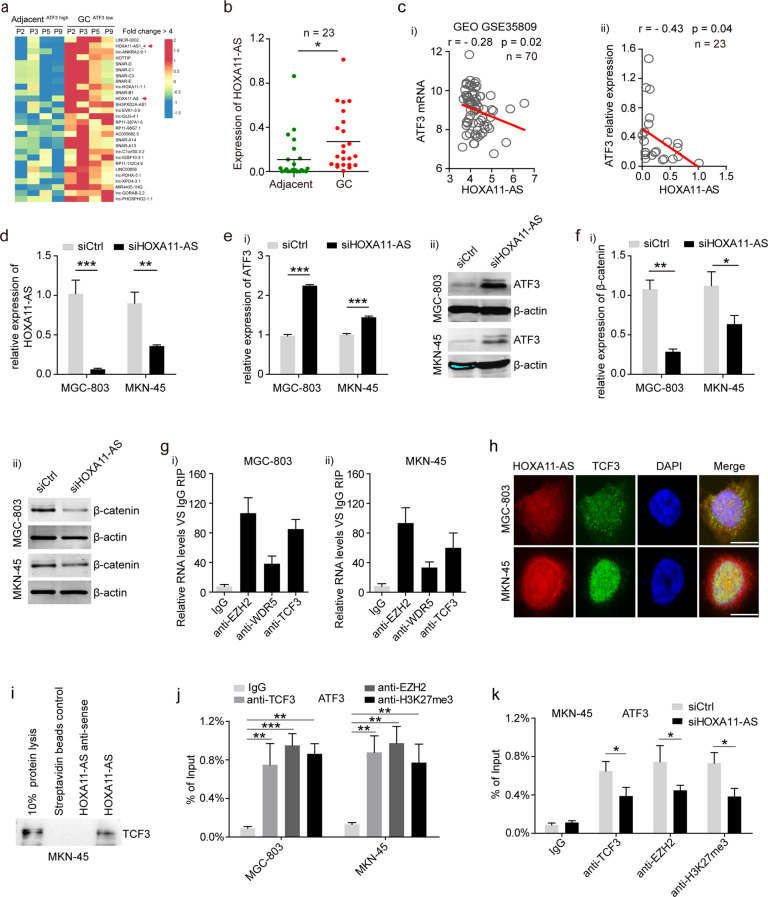
Increased HOXA11-AS expression suppressed ATF3 transcription in GC cells. **a** Heatmap of altered lncRNAs (fold change > 4, *p* < 0.05) in ATF3^low^ GC tissues compared with paired adjacent normal tissues. Red arrows indicate a significant increase in lncRNA HOXA11-AS expression in ATF3^low^ GC tissues. **b** The expression level of HOXA11-AS in GC tissues compared with that in paired normal gastric tissues as detected by RT-qPCR in our GC samples. **c** Correlation between the mRNA levels of HOXA11-AS and ATF3 in the GEO GSE35809 dataset (i) and in our GC samples (ii). **d** The expression level of HOXA11-AS was detected by RT-qPCR in HOXA11-AS-knockdown MGC-803 and MKN-45 cells. **e** ATF3 mRNA (i) and protein levels (ii) were detected by RT-qPCR and western blot, respectively, in HOXA11-AS-knockdown MGC-803 and MKN-45 cells, with β-actin serving as an endogenous control. **f** RT-qPCR and western blotting were performed to detect the mRNA (i) and protein (ii) levels, respectively, of β-catenin in HOXA11-AS-knockdown MGC-803 and MKN-45 cells, with β-actin serving as an endogenous control. **g** RIP analyses of HOXA11-AS1 and its predicted binding partners in MGC-803 (i) and MKN-45 cells (ii). **h** The colocalization of HOXA11-AS and TCF3 was analyzed by concomitant FISH and IF in MGC-803 and MKN-45 cells. Red, HOXA11-AS; Green, TCF3; blue, DAPI. Scale bar: 10 μm. **i** Western blotting analyses of TCF3 expression in MKN-45 cells subjected to pull-down with full-length HOXA11-AS. **j** ChIP-qPCR analysis of TCF3, EZH2, and H3K27me3 occupancy in the ATF3 promoter in MGC-803 and MKN-45 cells. **k** ChIP-qPCR analysis of TCF3, EZH2, and H3K27me3 occupancy in the ATF3 promoter in HOXA11-AS-knockdown MGC-803 cells. *p < 0.05; **p < 0.01 and ***p < 0.001 by Student’s t test compared with the corresponding control group.

Long noncoding RNAs (lncRNAs) are a highly heterogeneous group of transcripts that regulate gene expression by means of diverse mechanisms. To elucidate whether ATF3 expression was regulated by any endogenous lncRNAs in GC cells, the expression profile of lncRNAs in ATF3^low^ GC tissues was analyzed, and HOXA11-AS was found to be increased significantly in ATF3^low^ GC tissues (Fig. 6a). The analysis of our GC samples and online data confirmed highly expressed HOXA11-AS in GC tissues (Fig. 6b and Supplementary Fig. 6a-i, -ii). In addition, HOXA11-AS was found to be negatively correlated with ATF3 expression in both the GSE35809 dataset and our GC samples (Fig. 6c-i, -ii). To investigate whether abnormally elevated HOXA11-AS in GC cells contributes to ATF3 repression, HOXA11-AS was specifically inhibited in MGC-803 and MKN-45 cells (Fig. 6d). RT-qPCR and western blot assays showed that ATF3 expression was significantly increased upon HOXA11-AS inhibition (Fig. 6e-i, -ii).

HOXA11-AS could bind with WDR5 and promote β-catenin expression in GC cells^32^. Our data showed that activation of β-catenin/TCF3 signaling inhibited ATF3 expression, suggesting that increased HOXA11-AS expression results in its binding to WDR5 and promotion of β-catenin transcription, thereby inhibiting ATF3 in GC cells. To test this hypothesis, we first analyzed WDR5 expression in online databases and our GC samples, and WDR5 expression was found to be significantly upregulated (Supplementary Fig. 6b-i, -ii, -iii). Further analysis found that HOXA11-AS expression was positively correlated with β-catenin in the TCGA STAD specimens and the GSE66229 dataset (Supplementary Fig. 6c-i, -ii). In addition, WDR5 expression was positively correlated with β-catenin (Supplementary Fig. 6d-i, -ii). Next, RT-qPCR and western blot assays confirmed that specific inhibition of HOXA11-AS suppressed β-catenin expression in GC cells (Fig. 6f-i, -ii). Furthermore, the expression of WDR5 was downregulated by specific siRNA in MGC-803 and MKN-45 cells (Supplementary Fig. 6e-i, -ii and 6f). RT-qPCR and western blot assays of these cells showed that β-catenin expression was also significantly reduced (Supplementary Fig. 6g-i, -ii and 6h-i, -ii). ChIP assays confirmed that WDR5 could directly bind to the β-catenin promoter (Supplementary Fig. 6i) and that knockdown of HOXA11-AS reduced the deposition of WDR5 and H3K4me3 at the β-catenin promoter in MGC-803 and MKN-45 cells (Supplementary Fig. 6j-i, -ii).

A recent study reported that HOXA11-AS can interact with several RNA binding proteins, including EZH2 (H3K27me3), WDR5 (H3K4me3), LSD1 (H3K4me3) and DNMT1^33^. In addition, ATF3 expression could be regulated by the lncRNA ASBEL-TCF3 complex in CRC^13^, suggesting that HOXA11-AS binds to TCF3 and recruits chromatin modifying enzymes, thereby regulating ATF3 expression. To explore the mechanism by which HOXA11-AS mediated ATF3 expression in GC cells, bioinformatic analysis was performed to predict the interaction probability of HOXA11-AS and RNA-binding proteins by RNA-protein Interaction Prediction (http://pridb.gdcb.iastate.edu/RPISeq/), which indicated that HOXA11-AS may bind to EZH2, WDR5 and TCF3 (RF or SVM score >0.5; Supplementary Fig. 7a). We employed RIP with a panel of antibodies and confirmed that HOXA11-AS directly binds to chromatin modifiers EZH2 (H3K27me3), WDR5 (H3K4me3), and TCF3 in MGC-803 and MKN-45 cells (Fig. 6g-i, -ii). The interaction between HOXA11-AS and WDR5 or TCF3 was further confirmed by concomitant FISH and immunofluorescence (IF) assays. HOXA11-AS and either WDR5 or TCF3 were more prevalently expressed and colocalized in the nuclei of MGC-803 and MKN-45 cells (Fig. 6h and Supplementary Fig. 7b-i, -ii). In addition, an RNA pull-down assay revealed that TCF3 was detected in the retrieved RNA pull-down proteins from HOXA11-AS-overexpressing MKN-45 cells (Fig. 6i). Furthermore, ChIP analysis revealed that TCF3 and EZH2 could bind to the ATF3 promoter region (Fig. 6j), and HOXA11-AS knockdown reduced the binding efficiency of TCF3 and EZH2 in MGC-803 and MKN-45 cells (Fig. 6k and Supplementary Fig. 7c). Taken together, our data suggest that increased HOXA11-AS levels regulate ATF3 expression through the combination of indirect WDR5/HOXA11-AS signaling and direct TCF3/HOXA11-AS signaling in GC cells.

### TCF3-transactivated WDR5 promoted the expression of β-catenin, miR-17-5p, and HOXA11-AS

**Fig. 7:**
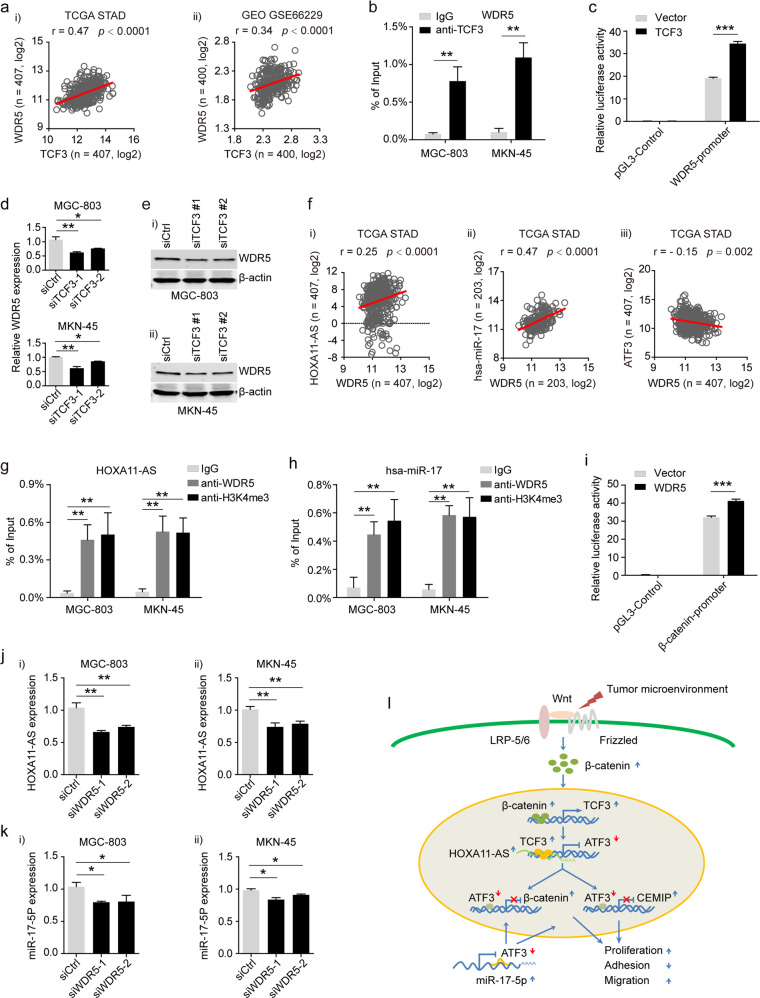
WDR5 was transactivated by TCF3 and promoted the expression of miR-17-5p and HOXA11-AS. **a** Correlation between the mRNA levels of TCF3 and WDR5 in the TCGA STAD specimens (i) and the GEO GSE66229 dataset (ii). **b** ChIP-qPCR analysis of TCF3 occupancy in the WDR5 promoter in MGC-803 and MKN-45 cells. **c** The promoter activity of WDR5 was measured by luciferase reporter gene analysis in HEK293T cells cotransfected with vector containing the target promoter upstream of luciferase and TCF3 or empty vector control. The data are presented as the mean ± s.d. **d**, **e** RT-qPCR and western blot analysis of WDR5 mRNA (**d**) and protein levels (**e**-**i**, -**ii**), respectively, in TCF3 knockdown MGC-803 and MKN-45 cells. **f** Correlations between the expression level of WDR5 and HOXA11-AS (i), miR-17 (ii), or ATF3 (iii) in the TCGA STAD specimens. **g**, **h** ChIP-qPCR analysis of WDR5 and H3K4me3 occupancy in the HOXA11-AS promoter (**g**) and miR-17 promoter (**h**) in MGC-803 and MKN-45 cells. **i** Promoter activity of β-catenin as measured by luciferase reporter assay in HEK293T cells cotransfected with vector containing the β-catenin promoter upstream of the luciferase gene and either WDR5 or empty vector controls. The data are presented as the mean ± s.d. *n* = 3. **j**, **k** RT-qPCR analysis of HOXA11-AS (**j**) and miR-17-5p RNA levels (**k**) in WDR5-knockdown MGC-803 (i) and MKN-45 cells (ii). **l** A schematic diagram illustrating the proposed molecular mechanisms of ATF3 inhibition in GC cells and its biological role in the progression of GC. *p < 0.05; **p < 0.01 and ***p < 0.001 by Student’s t test compared with the control group.

Available mouse TCF3 ChIP-seq data showed that TCF3 could bind to several regions of the WDR5 promoter in a variety of mouse cells (Supplementary Fig. 8a), suggesting that TCF3 regulates WDR5 in human GC cells. To verify our hypothesis, we first analyzed the correlation of TCF3 and WDR5 in both the TCGA STAD and GEO datasets. WDR5 expression was positively associated with TCF3 expression (Fig. 7a-i, -ii). ChIP assays indicated that TCF3 could directly bind to the WDR5 promoter (Fig. 7b). Consistently, upregulated TCF3 significantly increased the luciferase activity downstream of the WDR5 promoter relative to that in the control group (Fig. 7c). Furthermore, we found that the expression level of WDR5 was significantly decreased in TCF3-knockdown MGC-803 and MKN-45 cells (Figs. 7d, 7e-i, and e-ii). WDR5 is a core subunit of histone H3Lys4 (H3K4) methyltransferase complexes and an “effector” of HOX gene activation in human cells^34^. HOXA11 and its antisense transcript HOXA11-AS, both of which are members of the HOX gene family, overlap in the 5ʹUTR region in a head-to-head manner. The expression of HOXA11-AS was positively associated with HOXA11 in GC (Supplementary Fig. 8b-i, -ii). Available WDR5 ChIP-seq data showed that WDR5 binds to several regions of the β-catenin, miR-17 and HOXA11-AS promoters (Supplementary Fig. 8c–e). In addition, we found high enrichment of H3K4me3 and H3K27Ac modifications at the promoters of β-catenin, miR-17 and HOXA11-AS (Supplementary Fig. 8c–e), indicating that, in addition to promoting β-catenin expression, TCF3-transactivated WDR5 may promote the expression of HOXA11-AS and miR-17 in GC cells. To verify our hypothesis, we first analyzed the correlation of WDR5 and HOXA11-AS, hsa-miR-17 or ATF3 in the TCGA STAD and GEO datasets and found that WDR5 was positively correlated with HOXA11-AS or hsa-miR-17 but negatively associated with ATF3 (Fig. 7f-i, -ii, -iii and Supplementary Fig. 8f-i, -ii, -iii). Next, by performing ChIP assays, we observed increased WDR5 and H3K4me3 deposition in MGC-803 and MKN-45 cells at the promoters of HOXA11-AS and miR-17, respectively (Fig. 7g, h). A luciferase reporter assay also confirmed that WDR5 promoted the transactivation of β-catenin (Fig. 7i). In addition, HOXA11-AS and miR-17-5p expression was significantly reduced in WDR5-knockdown MGC-803 and MKN-45 cells (Fig. 7j-i, -ii and 7k-i, k-ii). In summary, our data indicate that TCF3 and WDR5 are abnormally elevated in GC cells, and WDR5 can be activated by TCF3 and promote the expression of HOXA11-AS and miR-17-5p.

## Discussion

Our data demonstrated an important role for decreased ATF3 expression in the stimulation of growth potential and carcinogenesis in GC cells. ATF3 repressed AGS and HGC-27 cells, which consequently exhibited increased cell proliferation and tumorigenic features. Conversely, ATF3 overexpression in MGC-803 and MKN-45 cells inhibited GC progression. Our in vivo results strongly supported the in vitro data: after injection of ATF3-shRNA/AGS cells into nude mice, tumor growth and pulmonary metastasis were significantly increased compared with those in control mice. On the other hand, significant decreases in the tumor growth of MGC-ATF3 cells were observed in nude mice. These results indicate that decreased ATF3 induced phenotypic transformation, causing GC cells to acquire more invasive and migratory properties and thus facilitating the capacity to metastasize. Consistently, in prostate cancer, ATF3 is believed to mediate the effect of androgen receptors and represses the androgen signaling required for sustaining prostate cancer cell proliferation and survival^30^. Ablation of ATF3 in a mouse model induced genome instability and promoted spontaneous tumorigenesis in different tissues^16^. Given the involvement of ATF3 in GC progression, our objective was to evaluate relevant ATF3 target genes and determined that β-catenin and CEMIP, which are closely related to the Wnt signaling pathway, were significantly upregulated in GC cells upon ATF3 downregulation.

β-catenin is one of the core proteins in the Wnt signaling pathway^7,8,33^. During the biological progression of cancer, β-catenin is known to shuttle between the cytosolic and nuclear compartments. This contributes to the oncogenic role of β-catenin: when it is hyperactivated by Wnt signaling to induce its stabilization and subsequent activation of the target genes required for cell proliferation, such as c-Myc^35^ and cyclin D1^36^. Previous studies have shown that the abnormal expression of β-catenin was related to the occurrence and development of GC^14,33,37^, which is consistent with our results that inactivation of β-catenin signaling via treatment with XAV-939 significantly inhibited ATF3 silencing-induced cell colony formation. Importantly, ATF3 silencing markedly enhanced the expression of β-catenin at both the mRNA and protein levels, whereas ATF3 overexpression decreased β-catenin transcriptional activity and protein expression, suggesting that tumorigenicity of downregulated ATF3 in GC cells is mediated, at least in part, by upregulating Wnt/β-catenin signaling activity.

CEMIP, also named KIAA1199, is a secreted factor that plays a role in extracellular ligand binding and processing^38^. CEMIP has been reported to be highly expressed in different cancers, including brain^31^, breast^39^, stomach^40^, and colon cancer^41^. Numerous studies have reported high expression levels of CEMIP in cancer cells and the relationship of CEMIP with invasive and metastatic disease mediated through the Wnt/β-catenin signaling pathway. For example, recent studies revealed that CEMIP promotes human osteoblastic stem cell migration via cooperative activation of canonical Wnt and p38/MAPK activity^38^. Exosomal CEMIP induces a proinflammatory state in the brain vascular niche that supports brain metastatic colonization^31^. In addition, in NCI-N87 and AGS cells, CEMIP upregulated Wnt/β-catenin signaling, and CEMIP deficiency reduced β-catenin expression^40^. Our results demonstrated that CEMIP is a direct transcriptional target of ATF3 in GC cells. ATF3 notably inhibited CEMIP promoter activity. ATF3 knockdown or overexpression significantly affected CEMIP expression at both the mRNA and protein levels. Furthermore, inhibition of CEMIP remarkably inhibited ATF3 silencing-induced cell migration and invasion. These findings suggest that reduced levels of ATF3 led to enhanced CEMIP expression and promoted GC progression.

In the current study, we further revealed that ATF3 is a novel downstream effector of canonical Wnt/β-catenin signaling and is directly transcriptionally repressed by activated β-catenin/TCF3 signaling in GC cells, suggesting negative feedback regulation of the β-catenin-TCF3-ATF3 axis. However, our finding was not in accordance with a previous study, which indicated that ATF3 is induced by Wnt or the ectopic expression of β-catenin and Tcf in breast and prostate cancer cells^42^. In addition, Inoue M et al. reported that activation of the canonical Wnt signaling pathway induces the expression of ATF3 in human colon cancer cells^43^. Interestingly, Taniue et al. recently demonstrated that transactivation of TCF3 by β-catenin is required for β-catenin-mediated proliferation of CRC cells, and ATF3 was specifically inhibited by TCF3 in CRC^13^, which is consistent with the data in the current study. The results observed in these studies were controversial, suggesting that the role of Wnt/β-catenin signaling in the transcriptional regulation of ATF3 in tumor cells is quite complex. In heterogeneous tumor cells, a single protein may play different roles in different tissues and stages or have differential transcriptional regulation depending on the cell type and state and context, which may result in the observation of opposing outcomes.

Recent reports have shown that, in addition to coding RNAs, a large number of noncoding RNAs play a crucial role in the regulation of gene expression in diverse biological pathways^14,32,33,40,44,45^. In CRC, Taniue et al. recently reported that ATF3 expression was regulated by the ASBEL-TCF3 complex^13^. Little is known about noncoding RNAs that contribute to ATF3 regulation in GC cells. We performed microRNA and lncRNA microarray screening on paired ATF3^low^ GC tissues and normal tissues and identified differential expression for hundreds of microRNAs and lncRNAs, among which miR-17-5p and HOXA11-AS had obvious differences. In the present study, we provide credible evidence for the first time that miR-17-5p and HOXA11-AS might be involved in regulating ATF3 expression and activity in GC cells. miR-17-5p, also known as miR-17 or oncomiR-1, is one of the members of the miRNA cluster miR-17-92^46^. Previous studies have reported that miR-17-5p is overexpressed in various cancers and is functionally involved in the regulation of malignancies in multiple cancers. For example, overexpressed miR-17-5p promoted the migration and invasion of breast cancer cells via direct binding to the 3′UTR of HMG box-containing protein 1 (HBP1) and subsequent activation of the Wnt/β-catenin pathway^47^. Overexpressed miR-17-5p promoted CRC progression by directly targeting the p130/Wnt/β-catenin pathway^46^. Additionally, HSP27, an oncogene related to the prognosis and metastasis of many cancers, has been identified to be modulated by miR-17-5p in HCC via p38 MAPK signaling^48^. In this study, we detected an inverse expression pattern between miR-17-5p and ATF3 in GC tissues and verified that ATF3 was a direct target of miR-17-5p, showing that the miR-17-5p inhibitor could promote ATF3 expression in GC cells. In addition, the inverse correlation was further confirmed in consecutive paraffin GC tissue sections. HOXA11-AS is the antisense RNA of HOXA11 in the HOX family. Several studies have reported the aberrant expression of HOXA11-AS in various cancers and its correlation with tumorigenesis and cancer development. In the current study, we found that HOXA11-AS was overexpressed in GC cells and negatively associated with ATF3 expression. Recently, Sun and Liu et al. reported that HOXA11-AS functions as a scaffold that could bind several RNA-binding proteins (WDR5, PRC2, LSD1, and DNMT1) and inhibit PRSS8 and KLF2 at the transcriptional level, thereby further stimulating β‐catenin protein and promoting GC progression^32,33^. Our findings confirmed that HOXA11-AS silencing markedly enhanced the expression of β-catenin at both the mRNA and protein levels. Moreover, consistent with the predictions of the bioinformatics tools, the RIP data, FISH/IF costaining, and RNA pull-down assays indicated that HOXA11-AS directly binds with WDR5 and TCF3. In addition, several studies have demonstrated that the lncRNA-mediated assembly of PRC2 and other RNA-binding proteins coordinates coupled H3K27 methylation of PRC2 in both mammalian and plant cells^49,50^. Indeed, our ChIP assay showed that HOXA11-AS knockdown reduced the TCF3 and EZH2 binding efficiency and H3K27me3 modifications in the ATF3 promoter in GC cells. To date, there has been no relevant research report on the regulation of ATF3 mediated by miR-17-5p and HOXA11-AS. The present study marks the first evidence of this interaction and suggests that aberrant expression of noncoding RNAs is an important step for GC progression mediated by the loss of ATF3 expression.

We also found that WDR5 and TCF3 were highly expressed and positively associated with each other in GC cells. We provide reliable evidence for the first time that WDR5 is a direct target of TCF3, suggesting a positive feedback loop of the β‐catenin-TCF3-WDR5 axis in GC cells. As a member of the WD40 repeat protein family, WDR5 is a core subunit of the histone H3K4 methyltransferase complex and mediates H3K4 methylation during gene transactivation^51^. WDR5 function is important for the maintenance of HOX gene expression in human cells^34,51^. Furthermore, as described in a recent report and confirmed in the current study, WDR5 could bind to HOXA11-AS and promote β-catenin expression in GC. Thus, we investigated whether WDR5 also played a role in the aberrant expression of HOXA11-AS and miR-17-5p, as WDR5 was positively associated with β-catenin, HOXA11-AS, and miR-17 in GC cells. Our data showed that higher levels of H3K4me3 and WDR5 were observed in the promoter loci of HOXA11-AS and hsa-miR-17. In addition, siRNA-mediated WDR5 knockdown in vitro significantly inhibited the expression of HOXA11-AS and hsa-miR-17 in GC cells. Thus, our data show that abnormal activation of the β-catenin/TCF3/WDR5 signaling axis in the GC environment leads to the abnormal expression of β-catenin, HOXA11-AS and miR-17-5p in GC cells.

Although we first reported that reduced ATF3 facilitates tumorigenesis of GC, there are still several limitations to our study. First, although we screened out the major target genes and signaling pathways that decreased ATF3 directly disturbed and performed serial in vitro experiments to validate the direct effects of ATF3 on GC tumorigenesis, in vivo experiments should be conducted to strengthen our hypothesis. In addition, the function of miR-17-5p and HOXA11-AS in the malignant transformation in GC cells promoted by ATF3 downregulation should be further validated in our experimental system, although many studies have reported that miR-17-5p and HOXA11-AS are highly expressed in GCs and a variety of other cancers and promote cancer progression. Finally, HOXA11-AS could serve as a scaffold and recruit multiple chromosome modifying enzymes to target specific genes, and the exact mechanism dictating HOXA11-AS-mediated suppression of ATF3 deserves further investigation.

In summary, our data demonstrated for the first time that abnormally activated Wnt/β-catenin/TCF3 signaling in GC cells, which cooperated with HOXA11-AS and miR-17-5p, promoted the downregulation of ATF3, which in turn elevated the expression of β-catenin and CEMIP and further enhanced the imbalanced Wnt/β-catenin signaling, synergistically accelerating the development of GC (Fig. 7l). Our data suggest that ATF3 acts as a brake in the Wnt/β-catenin signaling axis in normal gastric epithelial cells, which provides a targeting strategy using ATF3 and its regulatory factors as potential biomarkers and therapeutic targets for GC patients.

## Supplementary information

Supplementary Information for R1
