# Supplementary material for: Decreased expression of ATF3, orchestrated by β-catenin/TCF3, miR-17-5p and HOXA11-AS, promoted gastric cancer progression via increased β-catenin and CEMIP
Source: Exp Mol Med. 2021 Nov 2;53(11):1706–22. doi: 10.1038/s12276-021-00694-9 (PMC8639750; doi:10.1038/s12276-021-00694-9)
Supplement: Supplementary file 1 — Supplementary Information for R1 [file 12276_2021_694_MOESM1_ESM.pdf]

Supplementary Information

Decreased expression of ATF3, orchestrated by β-catenin/TCF3, miR-17-5p and HOXA11-AS, promoted gastric cancer progression via increased β-catenin and CEMIP

Guohua Xie[1*#], Ping Dong[2*], Hui Chen[1], Ling Xu[1], Yi Liu[1], Yanhui Ma[1], Yingxia Zheng[1], Junyao Yang[1], Yunlan Zhou[1], Lei Chen[2#], Lisong Shen[1, 3#]

[1] Department of Clinical Laboratory, Xinhua Hospital, Shanghai Jiao Tong University School of Medicine, Shanghai 200092, China;

[2] Department of General Surgery, Xinhua Hospital, Shanghai Jiao Tong University School of Medicine, Shanghai 200092, China;

[3] Faculty of Medical Laboratory Sciences, Ruijin Hospital, Shanghai Jiao Tong University School of Medicine, Shanghai 200030, China.

[*] These authors contributed equally to this work.

Supplementary Tables

Supplementary Table 1. The qRT-PCR primers. S, sense; AS, antisense

Genes	qRT-PCR Primers
ATF3	S: 5’-TTCTGAGCCCGGACAATACAC-3’
	AS: 5’-AAGAACGAGAAGCAGCATTTGAT-3’
β-actin	S: 5’-CCTGGCACCCAGCACAAT-3’
	AS: 5’-GCCGATCCACACGGAGTACT-3’
β-catenin	S: 5’-ACGGAGGAAGGTCTGAGGAG-3’
	AS: 5’-AGCCGCTTTTCTGTCTGGTT-3’
TCF3	S: 5’-CACGGCCTGCAGAGTAAGAT-3’
	AS: 5’-CCCTAGCCCACTGTAGGAGT-3’
WDR5	S: 5’-AGTGCCTCAAGACTTTGCCAGC-3’
	AS: 5’-CGATGAGCGTCTTCAGGCACTG-3’
HOXA11-AS	S: 5’-CGCGTAAGGCTGTGACATTG-3’
	AS: 5’-AGTTTTCTGGAGATGGCCCG-3’
DKK1	S: 5’-GTCTTTGTCGCGATGGTAGC-3’
	AS: 5’-GGGGGCAGGTTCTTGATAGC-3’
CEMIP	S: 5’-ACCGAGCACATTCCAACTACCG-3’
	AS: 5’-GGCAGAGATGATTGAGAGGAACG-3’
RAC2	S: 5’-CAGAGAGGCCTGAAAACCGT-3’
	AS: 5’-TAAGAAACGCCACAGGGAGG-3’
SMAD4	S: 5’-CCCATCCCGGACATTACTGG-3’
	AS: 5’-GCACACCTTTGCCTATGTGC-3’
TCF7L2	S: 5’-CTCCAAAGAAGTGTCGGGCA-3’
	AS: 5’-TCAGTCTGTGACTTGGCGTC-3’
PSEN1	S: 5’-GCGGGGAAGCGTATACCTAA-3’
	AS: 5’-ACGTACAGTATTGCTCAGGTG-3’
TBL1X	S: 5’-CTCGCGACGAGATTTCCGTG-3’
	AS: 5’-CGAGCTCGGTCATGCTACA-3’
LGR4	S: 5’-ACTCAGTTGCCAGAAGATGC-3’
	AS: 5’-CTCGAATGGCTTCACTGGGT-3’
SKP1	S: 5’-ACATGCAAGACTGTTGCCAAT-3’
	AS: 5’-ACTGTGTGCTACCTACCTGGG-3’
APC	S: 5’-GACTCGGAAATGGGGTCCAA-3’
	AS: 5’-TCTTCAGTGCCTCAACTTGCT-3’
CTBP1	S: 5’-GCATCCATCGAGATGCGAGA-3’
	AS: 5’-CCTATAGGCAGCCCCATTGA-3’
hsa-mir-17-5p	S: 5’-CAAAGTGCTTACAGTGCAGGTAG-3’
hsa-mir-331-3p	S: 5’-GCCCCTGGGCCTATCCTAGAA-3’
hsa-mir-505-5p	S: 5’-GGGAGCCAGGAAGTATTGATGT-3’
hsa-mir-590-5p	S: 5’-GAGCTTATTCATAAAAGTGCAG-3’

Supplementary Table 2. shRNA and siRNA sequences

shRNA	Sequence of oligonucleotides
sh-ATF3-1#	GCAAAGTGCCGAAACAAGA
sh-ATF3-2#	GAGAAACCTCTTTATCCAA
siRNA	
genOFFTM st-h-TCF3_001	CCGGATCACTCAAGCAATA
genOFFTM st-h-TCF3_002	GAACCTGAATCCCAAAGCA
genOFFTM st-h-TCF3_003	AGCCTCTCTTCATCCACAT
genOFFTM st-h-WDR5_001	GCGTGAGGATATGGGATGT
genOFFTM st-h-WDR5_002	GTCGTGATCTCAACAGCTT
genOFFTM st-h-WDR5_003	GGAAGTGCCTCAAGACTTT
genOFFTM st-h-CEMIP_001	GCACCATTCTGAACTTGGA
genOFFTM st-h-CEMIP_002	GGAAGACCATATTGAATAT
genOFFTM st-h-CEMIP_003	GCACCGAGGTTGTCTACAA
genOFFTM st-h-β-catenin_001	GATGTGGATACCTCCCAAG
genOFFTM st-h-β-catenin_002	GGTTAATAAGGCTGCAGTT
genOFFTM st-h-β-catenin_003	CTGATCTTGGACTTGATAT
RiboTM h-HOXA11-AS Smart Silencer	GGTGACTTGATTACACTCT
	CCTCTTCATCCCACCTTCT
	GGCGGAATATCGGAATAAA
	TTCTCACCGAAAGCACGTAA
	TTAGTGCCTTCCGTCCCTAA
	TTCCGAGGCTTCGTCTGACT

Supplementary Table 3. ChIP qPCR primers. S, sense; AS, antisense; TSS, transcription start site

Genes	qPCR Primers
TCF3_TSS	S: 5'-CATCTGCTCCGCGTTAATGC-3'
	AS: 5'-TTTGAAGCCACTTTGCACGG-3'
TCF3_-3000	S: 5'-TGGGGAAAACAATGGTGGCT-3'
	AS: 5'-GGCTCTTTCCTGTGAGGTCC-3'
ATF3_TSS	S: 5'-AAATTACCAAACTGTGACCTTCGG-3'
	AS: 5'-CCCGTGGATGACTATTTGTAGACA-3'
ATF3_+1500	S: 5'-TCCTCCTAAACATTTTCTTTTCCC-3'
	AS: 5'-GCTCTCCTTTTCTTTCTGGGTTCA-3'
H3K27me3	S: 5'-TCCCAGCCTCACCTAGTCTG-3'
	AS: 5'-TCCAGGCATGACCAATGGTG-3'
ATF3-EZH2	S: 5'-CAGAGGCAGGTGGAAAGGAG-3'
	AS: 5'-CTTCTTGACTGCCCGTGGAT-3'
β-catenin-TSS	S: 5'-GACTTATAAGAGCTCCTTGTGCGG-3'
	AS: 5'-GACTGAAGCTGCTCCTCAGAC-3'
H3K4me3-1	S: 5'-CATTTTAAGCCTCTCGGTCTGTG-3'
	AS: 5'-GAAGCTGCTCCTCAGACCTTC-3'

WDR5-TSS	S: 5’-CAGGTGCTTTCCAAATGCCC-3’
	AS: 5’-CCCTGTACACAGACACCAGC-3’
HOXA11-AS TSS	S: 5’-GGGTGGTGGTAGACGTTGG-3’
	AS: 5’-CAATCTGGCCCACTGCTACT-3’
H3K4me3-2	S: 5’-AACTGGTCGAAAGCCTGTGG-3’
	AS: 5’-AAGAGCTCGGCCAACGTCTA-3’
mir-17 TSS	S: 5’-ACTGAAAAAGGCAGGCTCGT-3’
	AS: 5’-CCAATCAAGACCTCGTGGCT-3’
H3K4me3-3	S: 5’-ACTGAAAAAGGCAGGCTCGT-3’
	AS: 5’-CCAATCAAGACCTCGTGGCT-3’

Supplementary Table 4. The clinic-pathological factors of 90 GC patients

Characteristics	Expression of ATF3		*p* value*
	low	high	
Sex			0.172
male	34	28	
female	11	17	
Age			0.829
≤ 65	17	18	
> 65	28	27	
Tumor size			0.006*
≤ 5 cm	15	28	
> 5 cm	30	17	
Histological grade			0.399
Low	24	20	
middle or high	21	25	
Serosal invasion			0.014*
Absent	6	16	
Present	39	29	
Lymphatic invasion			0.598
Absent	35	37	
Present	10	8	
Peritoneal dissemination			0.049*
Absent	39	44	
Present	6	1	
Tumor invasion depth (T)			0.038*
T1/T2	9	18	
T3/T4	36	27	
Lymph node metastasis (N)			0.153
Absent	9	15	
Present	36	30	
TNM stage			0.136
I/II	16	23	

III/IV	29	22	

*chi-square test
* p<0.05

Supplementary Table 5. Univariate and multivariate analysis of clinic-pathologic factors for overall survival in 90 patients with GC

Risk factors	Univariate analysis			Multivariate analysis		
	HR	p value	95% CI	HR	p value	95% CI
Sex (male, female)	0.558	0.057	0.306～1.018	0.722	0.308	0.386～1.350
Age (≤ 65, > 65)	1.231	0.442	0.724～2.094	1.378	0.296	0.755～2.512
Tumor size (≤5 cm, >5 cm)	4.433	<0.001**	2.479～7.925	2.917	0.005**	1.393～6.110
Histological grade (low, middle or high)	0.536	0.018*	0.321～0.897	0.711	0.268	0.388～1.301
Serosal invasion (Absent, Present)	0.374	0.007**	0.183～0.765	2.293	0.238	0.578～9.103
Lymphatic invasion (Absent, Present)	0.689	0.236	0.373～1.275	0.829	0.589	0.420～1.637
Peritoneal dissemination (Absent, Present)	0.359	0.012*	0.161～0.799	0.882	0.808	0.320～2.429
Tumor invasion depth(T1/T2, T3/T4)	2.217	0.012*	1.194～4.115	0.778	0.568	0.329～1.840
Lymph node metastasis (N0, N1)	0.391	0.007**	0.198～0.774	1.736	0.310	0.599～5.033
TNM stage (I/II, III/IV)	3.667	<0.001**	2.046～6.573	1.735	0.237	0.696～4.323
ATF3 expression	0.413	0.001**	0.245～0.696	0.552	0.032*	0.320～0.950

HR hazard ratio
* p<0.05
** p<0.01

Supplementary Table 6. mRNAs increased abundance (FC≥2-fold, p<0.05) in ATF3-knockdown AGS cells

gene_id	gene_name	FPKM.shATF3_1	FPKM.shATF3_2	FPKM.shATF3_3	FPKM.shNC_1	FPKM.shNC_2	FPKM.shNC_3	log2(fc)	P value	regulation	significant
ENSG00000124208	TMEM189-UBE2V1	0	0	0	6.60	0	10.21	-inf	0.00	down	yes
ENSG00000257529	RPL36A-HNRNPH2	0	0	0	50.15	49.07	0	-inf	0.00	down	yes
ENSG00000169059	VCX3A	2.87	5.43	5.23	0	0	0	inf	0.00	up	yes
ENSG00000275896	PRSS2	2.05	1.80	1.71	0	0	0	inf	0.00	up	yes
ENSG00000205642	VCX3B	0.79	1.03	1.12	0	0	0	inf	0.00	up	yes
ENSG00000285238	AC006064	4.00	11.13	0	0	0	0	inf	0.00	up	yes
ENSG00000269711	AC008763	11.57	4.40	0	0	0	0	inf	0.00	up	yes
ENSG00000277502	RF02271	5.76	0.01	6.73	0.01	0.01	0.01	8.74	0.00	up	yes
ENSG00000285990	AL589743	2.09	1.84	0.58	0	0	0.04	6.88	0.00	up	yes
ENSG00000055118	KCNH2	0.23	0.20	0.32	0	0	0.01	6.56	0.00	up	yes
ENSG00000279864	AC124864	3.35	2.74	3.09	0.07	0.04	0	6.35	0.00	up	yes
ENSG00000281181	FP236383	5747.82	5236.37	2494.30	109.58	153.87	153.02	5.02	0.00	up	yes
ENSG00000280800	FP671120	1416.21	1327.57	637.64	28.24	40.21	40.80	4.95	0.00	up	yes
ENSG00000281383	FP671120	789.96	727.38	348.14	15.80	21.53	23.44	4.94	0.00	up	yes
ENSG00000280614	FP236383	3305.68	3043.59	1451.71	63.63	104.64	89.59	4.92	0.00	up	yes
ENSG00000137868	STRA6	0.16	0.11	0.18	0	0.01	0.01	4.81	0.00	up	yes
ENSG00000258659	TRIM34	1.91	2.17	2.01	0	0.01	0.22	4.71	0.00	up	yes
ENSG00000197859	ADAMTSL2	6.47	5.93	6.54	0.29	0.17	0.27	4.68	0.00	up	yes
ENSG00000285269	AL160269	0	1.83	1.93	0	0.15	0	4.67	0.01	up	yes
ENSG00000185479	KRT6B	0.61	0.60	0.53	0	0	0.07	4.65	0.00	up	yes
ENSG00000277209	RPPH1	60.04	51.16	30.58	3.40	1.01	1.29	4.64	0.00	up	yes
ENSG00000142973	CYP4B1	0.26	0.25	0.16	0	0.03	0	4.53	0.00	up	yes
ENSG00000283293	RN7SK	119.09	107.58	53.90	6.70	3.33	3.62	4.36	0.00	up	yes

ENSG00000074047	GLI2	0.37	0.41	0.41	0.03	0	0.04	4.16	0.00	up	yes
ENSG00000185909	KLHDC8B	0.49	0.59	0.90	0.07	0	0.04	4.09	0.00	up	yes
ENSG00000197273	GUCA2A	1.47	1.55	1.40	0	0.11	0.16	4.01	0.00	up	yes
ENSG00000107719	PALD1	6.18	6.18	7.35	0.48	0.32	0.42	4.01	0.00	up	yes
ENSG00000100453	GZMB	1.20	2.04	1.50	0	0.19	0.10	4.01	0.00	up	yes
ENSG00000196954	CASP4	0.55	0.42	0.62	0.02	0.07	0.01	3.97	0.00	up	yes
ENSG00000239322	ATP6V1B1-AS1	0.05	1.44	0.74	0	0.10	0.05	3.92	0.00	up	yes
ENSG00000182583	VCX	0.99	0.54	1.43	0.21	0	0	3.84	0.00	up	yes
ENSG00000278189	RNA5-8SN1	33.61	40.63	18.88	1.91	2.38	2.48	3.78	0.00	up	yes
ENSG00000256542	AC148477	1.13	1.28	0.93	0.08	0.12	0.04	3.78	0.00	up	yes
ENSG00000197249	SERPINA1	1.20	0.86	0.99	0.08	0.10	0.04	3.78	0.00	up	yes
ENSG00000167656	LY6D	5.41	5.31	5.56	0.53	0.29	0.39	3.75	0.00	up	yes
ENSG00000196189	SEMA4A	0.23	0.14	0.06	0.01	0.01	0.01	3.68	0.00	up	yes
ENSG00000263934	SNORD3A	7.97	0.33	4.92	0.46	0.10	0.50	3.64	0.00	up	yes
ENSG00000184357	HIST1H1B	1.44	2.02	0.76	0	0.28	0.07	3.62	0.00	up	yes
ENSG00000277739	RF00002	48.70	53.97	24.85	3.01	3.52	3.85	3.62	0.00	up	yes
ENSG00000207513	RNU1-3	6.21	13.71	33.20	2.24	1.09	1.13	3.57	0.00	up	yes
ENSG00000275215	RNA5-8SN3	144.00	161.88	77.19	11.06	12.18	11.27	3.47	0.00	up	yes
ENSG00000278233	RNA5-8SN2	100.00	110.07	51.01	8.13	8.36	7.88	3.42	0.00	up	yes
ENSG00000182463	TSHZ2	0.30	0.34	0.34	0.01	0.05	0.03	3.40	0.00	up	yes
ENSG00000133135	RNF128	0.66	0.58	0.78	0.08	0	0.11	3.40	0.00	up	yes
ENSG00000183486	MX2	0.22	0.25	0.25	0.03	0.02	0.02	3.38	0.00	up	yes
ENSG00000183647	ZNF530	0.18	0.06	0.17	0.03	0.01	0	3.37	0.00	up	yes
ENSG00000221890	NPTXR	0.29	0.50	0.37	0.07	0.02	0.02	3.29	0.00	up	yes
ENSG00000077984	CST7	1.14	1.20	1.21	0.28	0	0.11	3.22	0.00	up	yes

ENSG00000088002	SULT2B1	6.67	6.17	7.06	0.67	0.76	0.74	3.20	0.00	up	yes
ENSG00000117472	TSPAN1	3.15	3.85	4.01	0.30	0.43	0.47	3.19	0.00	up	yes
ENSG00000145721	LIX1	0.63	0.44	0.27	0.03	0.07	0.05	3.18	0.00	up	yes
ENSG00000255508	AP002990	0.79	0.77	156.58	8.27	7.55	2.18	3.14	0.02	up	yes
ENSG00000240065	PSMB9	0.59	0.54	0.75	0.09	0.02	0.11	3.09	0.00	up	yes
ENSG00000109819	PPARGC1A	0.37	0.41	0.31	0.04	0.04	0.05	3.05	0.00	up	yes
ENSG00000021826	CPS1	0.11	0.11	0.15	0.01	0.01	0.02	3.04	0.00	up	yes
ENSG00000167741	GGT6	1.32	1.19	1.38	0.20	0.19	0.08	3.03	0.00	up	yes
ENSG00000137843	PAK6	0.16	0.21	0.16	0.03	0.02	0.02	2.93	0.00	up	yes
ENSG00000108947	EFNB3	1.58	1.64	1.65	0.10	0.18	0.36	2.92	0.00	up	yes
ENSG00000277027	RMRP	69.39	60.90	34.98	14.33	4.95	3.21	2.88	0.00	up	yes
ENSG00000165125	TRPV6	0.25	0.16	0.29	0.03	0.02	0.04	2.85	0.00	up	yes
ENSG00000258590	NBEAP1	1.16	1.19	1.40	0.14	0.17	0.21	2.85	0.00	up	yes
ENSG00000103888	CEMIP	4.67	3.95	4.53	0.58	0.55	0.69	2.85	0.00	up	yes
ENSG00000105419	MEIS3	2.78	2.62	3.23	0.44	0.37	0.40	2.84	0.00	up	yes
ENSG00000113657	DPYSL3	14.26	13.95	14.31	1.92	2.27	1.79	2.83	0.00	up	yes
ENSG00000134363	FST	0.36	0.33	0.26	0.03	0.07	0.04	2.83	0.00	up	yes
ENSG00000272674	PCDHB16	0.44	0.43	0.52	0.06	0.05	0.08	2.82	0.00	up	yes
ENSG00000129451	KLK10	0.37	0.28	0.31	0.09	0.02	0.03	2.82	0.00	up	yes
ENSG00000244694	PTCHD4	1.07	0.78	0.96	0.17	0.07	0.16	2.81	0.00	up	yes
ENSG00000223764	LINC02593	0.34	0.41	0.26	0.03	0.03	0.08	2.77	0.00	up	yes
ENSG00000136634	IL10	1.38	1.22	1.14	0.26	0.20	0.09	2.76	0.00	up	yes
ENSG00000231431	FAR2P4	0.25	0.42	0.13	0.09	0.01	0.02	2.75	0.00	up	yes
ENSG00000103740	ACSBG1	0.19	0.06	0.17	0.02	0	0.04	2.75	0.00	up	yes
ENSG00000164796	CSMD3	0.04	0.06	0.03	0.02	0	0.00	2.74	0.00	up	yes

ENSG00000188641	DPYD	0.13	0.21	0.14	0.04	0	0.04	2.73	0.00	up	yes
ENSG00000203783	PRR9	48.52	45.49	47.80	7.44	6.74	7.44	2.71	0.00	up	yes
ENSG00000206596	RNU1-27P	13.93	9.05	15.08	1.50	1.88	2.45	2.71	0.00	up	yes
ENSG00000166833	NAV2	6.42	6.31	8.98	1.14	1.12	1.06	2.71	0.00	up	yes
ENSG00000221963	APOL6	1.98	1.79	1.68	0.33	0.23	0.29	2.70	0.00	up	yes
ENSG00000125378	BMP4	0.46	0.40	0.47	0.02	0.06	0.12	2.69	0.00	up	yes
ENSG00000133121	STARD13	0.09	0.11	0.12	0	0.02	0.03	2.67	0.00	up	yes
ENSG00000064195	DLX3	0.52	0.41	0.35	0.04	0.02	0.14	2.66	0.00	up	yes
ENSG00000119698	PPP4R4	0.28	0.46	0.34	0.01	0.09	0.06	2.65	0.00	up	yes
ENSG00000153029	MR1	1.82	1.81	1.89	0.24	0.36	0.31	2.60	0.00	up	yes
ENSG00000165078	CPA6	2.46	3.04	2.81	0.61	0.34	0.43	2.59	0.00	up	yes
ENSG00000145246	ATP10D	1.23	1.28	1.25	0.22	0.19	0.21	2.58	0.00	up	yes
ENSG00000115616	SLC9A2	1.77	1.97	1.73	0.32	0.26	0.33	2.58	0.00	up	yes
ENSG00000006468	ETV1	0.30	0.19	0.25	0.07	0.01	0.04	2.57	0.00	up	yes
ENSG00000081803	CADPS2	2.16	2.13	2.00	0.33	0.42	0.31	2.56	0.00	up	yes
ENSG00000029534	ANK1	4.97	4.89	4.86	0.86	0.82	0.82	2.56	0.00	up	yes
ENSG00000167914	GSDMA	0.54	0.33	0.44	0.06	0.14	0.02	2.55	0.00	up	yes
ENSG00000270885	RASL10B	1.63	1.51	1.41	0.26	0.20	0.32	2.54	0.00	up	yes
ENSG00000261611	AC010547	0.76	1.34	1.12	0.45	0	0.11	2.53	0.01	up	yes
ENSG00000139211	AMIGO2	3.15	3.46	3.42	0.61	0.52	0.61	2.53	0.00	up	yes
ENSG00000133216	EPHB2	4.84	4.75	4.89	0.81	0.83	0.87	2.53	0.00	up	yes
ENSG00000156127	BATF	11.60	11.14	11.97	1.89	2.00	2.22	2.51	0.00	up	yes
ENSG00000136542	GALNT5	3.10	3.37	3.31	0.65	0.46	0.62	2.50	0.00	up	yes
ENSG00000135519	KCNH3	0.25	0.29	0.12	0.07	0.01	0.04	2.48	0.00	up	yes
ENSG00000253771	TPTE2P1	0.09	0.28	0.24	0.01	0.03	0.07	2.48	0.00	up	yes

ENSG00000157502	PWWP3B	0.19	0.31	0.24	0.04	0.06	0.03	2.47	0.00	up	yes
ENSG00000168843	FSTL5	0.33	0.13	0.29	0.01	0.09	0.03	2.46	0.00	up	yes
ENSG00000156486	KCNS2	0.09	0.17	0.17	0.03	0.03	0.02	2.46	0.00	up	yes
ENSG00000140511	HAPLN3	1.18	0.70	0.92	0.14	0.12	0.24	2.46	0.00	up	yes
ENSG00000266992	DHX40P1	0.67	0.39	0.77	0.19	0.02	0.12	2.45	0.00	up	yes
ENSG00000215912	TTC34	0.17	0.18	0.14	0	0.03	0.06	2.44	0.00	up	yes
ENSG00000166143	PPP1R14D	1.13	1.10	1.11	0.08	0.15	0.39	2.44	0.00	up	yes
ENSG00000150551	LYPD1	3.63	4.17	3.04	0.82	0.76	0.44	2.43	0.00	up	yes
ENSG00000172575	RASGRP1	1.23	1.25	1.32	0.17	0.33	0.21	2.43	0.00	up	yes
ENSG00000168453	HR	0.67	0.66	0.66	0.19	0.02	0.16	2.42	0.00	up	yes
ENSG00000163686	ABHD6	5.72	5.49	5.14	0.98	1.09	1.00	2.41	0.00	up	yes
ENSG00000115221	ITGB6	1.41	1.48	1.55	0.39	0.24	0.21	2.41	0.00	up	yes
ENSG00000106123	EPHB6	1.41	1.28	1.07	0.18	0.32	0.23	2.38	0.00	up	yes
ENSG00000230937	MIR205HG	0.31	0.49	0.31	0.07	0.10	0.05	2.37	0.00	up	yes
ENSG00000089356	FXYD3	5.87	7.22	6.27	1.37	1.41	0.98	2.37	0.00	up	yes
ENSG00000185483	ROR1	2.37	2.43	2.32	0.47	0.55	0.37	2.36	0.00	up	yes
ENSG00000120318	ARAP3	1.14	1.28	1.31	0.35	0.15	0.23	2.35	0.00	up	yes
ENSG00000180385	EMC3-AS1	0.65	0.87	0.12	0.13	0.06	0.13	2.35	0.00	up	yes
ENSG00000197837	HIST4H4	0.29	0.41	0.35	0.14	0.06	0.00	2.35	0.00	up	yes
ENSG00000184584	TMEM173	0.72	0.93	0.98	0.15	0.20	0.17	2.35	0.00	up	yes
ENSG00000276168	RN7SL1	241.00	205.19	283.29	59.09	40.63	44.60	2.34	0.00	up	yes
ENSG00000267216	AC020915	0.51	0.31	7.51	0.82	0.38	0.46	2.33	0.01	up	yes
ENSG00000213965	NUDT19	0.44	0.55	0.40	0.05	0.07	0.16	2.32	0.00	up	yes
ENSG00000261801	LOXL1-AS1	0.29	0.89	0.95	0.16	0.10	0.17	2.32	0.00	up	yes
ENSG00000267475	AC008736	3.85	4.71	4.35	0.73	1.35	0.51	2.32	0.00	up	yes

ENSG00000278626	AC023310	1.18	1.48	1.45	0.20	0.25	0.38	2.32	0.00	up	yes
ENSG00000277224	HIST1H2BF	0.77	0.73	0.60	0.10	0.06	0.26	2.31	0.00	up	yes
ENSG00000198626	RYR2	0.08	0.08	0.09	0.00	0.03	0.02	2.31	0.00	up	yes
ENSG00000197261	C6orf141	1.02	1.02	1.26	0.23	0.25	0.19	2.31	0.00	up	yes
ENSG00000175344	CHRNA7	0.06	0.06	0.06	0.01	0.01	0.01	2.30	0.00	up	yes
ENSG00000128340	RAC2	2.77	2.32	3.24	0.44	0.68	0.58	2.29	0.00	up	yes
ENSG00000224586	GPX5	0.91	0.97	0.91	0.16	0.26	0.16	2.29	0.00	up	yes
ENSG00000143127	ITGA10	1.18	1.27	1.25	0.21	0.31	0.24	2.29	0.00	up	yes
ENSG00000163823	CCR1	0.88	0.64	0.99	0.16	0.18	0.18	2.27	0.00	up	yes
ENSG00000213949	ITGA1	0.12	0.15	0.13	0.04	0.01	0.03	2.27	0.00	up	yes
ENSG00000233922	LINC01694	1.07	1.31	1.01	0.24	0.22	0.25	2.25	0.00	up	yes
ENSG00000269881	AC004754	0.16	0.76	0.57	0.10	0.14	0.08	2.25	0.00	up	yes
ENSG00000234745	HLA-B	6.56	6.98	7.07	1.55	1.40	1.41	2.24	0.00	up	yes
ENSG00000198734	F5	1.06	1.28	1.14	0.27	0.23	0.26	2.21	0.00	up	yes
ENSG00000163536	SERPINI1	0.40	0.41	0.73	0.15	0.07	0.12	2.20	0.00	up	yes
ENSG00000241418	MCRIP2P1	0.05	2.49	3.00	1.02	0.07	0.14	2.18	0.04	up	yes
ENSG00000149294	NCAM1	0.62	0.79	0.75	0.15	0.16	0.17	2.17	0.00	up	yes
ENSG00000086696	HSD17B2	0.81	0.88	0.84	0.25	0.17	0.15	2.17	0.00	up	yes
ENSG00000124466	LYPD3	2.15	2.16	2.41	0.46	0.22	0.82	2.17	0.00	up	yes
ENSG00000283189	AC104452	0.44	0.37	0.46	0.01	0.13	0.15	2.16	0.00	up	yes
ENSG00000257588	AC025154	2.30	2.01	2.39	0.36	0.54	0.60	2.16	0.00	up	yes
ENSG00000172789	HOXC5	1.68	1.68	1.88	0.57	0.34	0.27	2.15	0.00	up	yes
ENSG00000161955	TNFSF13	3.59	2.83	4.47	0.87	0.87	0.73	2.14	0.00	up	yes
ENSG00000167601	AXL	1.89	2.01	2.10	0.52	0.50	0.34	2.14	0.00	up	yes
ENSG00000274012	RN7SL2	248.71	197.00	302.57	84.16	42.67	42.98	2.14	0.00	up	yes

ENSG00000140279	DUOX2	0.38	0.36	0.35	0.11	0.07	0.06	2.14	0.00	up	yes
ENSG00000203814	HIST2H2BF	1.11	0.55	0.48	0.14	0.21	0.15	2.13	0.00	up	yes
ENSG00000127124	HIVEP3	0.04	0.08	0.06	0.01	0.01	0.02	2.13	0.00	up	yes
ENSG00000123700	KCNJ2	0.14	0.17	0.17	0.02	0.03	0.05	2.12	0.00	up	yes
ENSG00000196220	SRGAP3	0.38	0.38	0.31	0.05	0.10	0.09	2.12	0.00	up	yes
ENSG00000283761	AC118553	0.40	2.16	7.85	0.45	1.67	0.29	2.11	0.01	up	yes
ENSG00000095321	CRAT	1.01	1.06	1.33	0.17	0.28	0.35	2.10	0.00	up	yes
ENSG00000197191	CYSRT1	1.96	2.29	2.69	0.28	0.78	0.56	2.09	0.00	up	yes
ENSG00000163565	IFI16	0.56	0.60	0.65	0.19	0.07	0.16	2.09	0.00	up	yes
ENSG00000181392	SYNE4	0.77	1.05	1.12	0.27	0.22	0.20	2.07	0.00	up	yes
ENSG00000182667	NTM	6.29	5.97	5.59	1.36	1.31	1.57	2.07	0.00	up	yes
ENSG00000253731	PCDHGA6	0.20	0.20	0.22	0.06	0.05	0.04	2.06	0.00	up	yes
ENSG00000168772	CXXC4	0.15	0.27	0.27	0.09	0.02	0.06	2.06	0.00	up	yes
ENSG00000133020	MYH8	0.15	0.09	0.14	0.01	0.05	0.04	2.06	0.00	up	yes
ENSG00000181577	C6orf223	0.30	0.37	0.32	0.15	0.07	0.03	2.05	0.00	up	yes
ENSG00000105711	SCN1B	0.87	0.82	0.74	0.29	0.19	0.10	2.05	0.00	up	yes
ENSG00000136059	VILL	0.55	0.31	0.38	0.08	0.11	0.11	2.04	0.00	up	yes
ENSG00000156515	HK1	6.19	6.26	6.36	1.57	1.64	1.36	2.04	0.00	up	yes
ENSG00000126460	PRRG2	3.42	4.37	4.34	0.94	0.91	1.11	2.04	0.00	up	yes
ENSG00000144730	IL17RD	3.95	4.03	3.67	0.83	1.13	0.89	2.03	0.00	up	yes
ENSG00000121797	CCRL2	0.36	0.17	0.52	0.05	0.15	0.07	2.03	0.00	up	yes
ENSG00000106003	LFNG	2.37	3.00	2.83	0.71	0.62	0.69	2.03	0.00	up	yes
ENSG00000179477	ALOX12B	0.33	0.47	0.66	0.17	0.03	0.16	2.02	0.00	up	yes
ENSG00000163082	SGPP2	0.62	0.80	0.68	0.12	0.20	0.20	2.01	0.00	up	yes
ENSG00000251364	AC107884	0.08	0.17	0.11	0.01	0.05	0.04	2.00	0.00	up	yes

ENSG00000256943	AC148477	0.34	0.54	1.12	0.23	0.11	0.17	1.99	0.00	up	yes
ENSG00000204802	AL590399	0.13	0.37	0.62	0.10	0.13	0.06	1.99	0.00	up	yes
ENSG00000132746	ALDH3B2	0.77	0.96	0.86	0.18	0.36	0.12	1.98	0.00	up	yes
ENSG00000183508	TENT5C	0.23	0.28	0.34	0.05	0.08	0.09	1.97	0.00	up	yes
ENSG00000148344	PTGES	0.57	1.06	1.02	0.20	0.26	0.21	1.97	0.00	up	yes
ENSG00000172426	RSPH9	0.34	0.77	0.40	0.17	0.13	0.09	1.96	0.00	up	yes
ENSG00000131370	SH3BP5	3.16	3.06	3.08	0.74	0.87	0.79	1.96	0.00	up	yes
ENSG00000234494	SP2-AS1	0.57	0.43	0.65	0.03	0.04	0.35	1.96	0.01	up	yes
ENSG00000174791	RIN1	0.76	0.76	0.80	0.14	0.22	0.24	1.95	0.00	up	yes
ENSG00000185133	INPP5J	0.27	0.38	0.37	0.10	0.06	0.11	1.95	0.00	up	yes
ENSG00000188404	SELL	2.54	2.03	2.05	0.53	0.62	0.57	1.95	0.00	up	yes
ENSG00000085117	CD82	1.78	1.63	1.15	0.36	0.46	0.36	1.95	0.00	up	yes
ENSG00000135374	ELF5	1.26	1.32	1.42	0.33	0.41	0.30	1.94	0.00	up	yes
ENSG00000174640	SLCO2A1	1.73	1.67	1.82	0.43	0.48	0.44	1.94	0.00	up	yes
ENSG00000107731	UNC5B	0.68	0.72	0.83	0.19	0.24	0.16	1.93	0.00	up	yes
ENSG00000162849	KIF26B	0.28	0.30	0.23	0.06	0.05	0.10	1.93	0.00	up	yes
ENSG00000178685	PARP10	0.50	0.47	0.75	0.10	0.17	0.18	1.93	0.00	up	yes
ENSG00000261659	Z92544	0.90	0.78	0.99	0.19	0.42	0.10	1.93	0.00	up	yes
ENSG00000140254	DUOXA1	0.33	0.18	0.40	0.09	0.11	0.04	1.92	0.00	up	yes
ENSG00000285725	AC004967	2.13	3.17	2.60	0.90	0.66	0.53	1.92	0.00	up	yes
ENSG00000188766	SPRED3	0.89	0.74	0.96	0.10	0.48	0.09	1.92	0.00	up	yes
ENSG00000019505	SYT13	7.99	8.11	7.92	2.05	2.17	2.16	1.91	0.00	up	yes
ENSG00000274588	DGKK	0.40	0.36	0.33	0.10	0.08	0.12	1.90	0.00	up	yes
ENSG00000241186	TDGF1	0.64	0.74	0.67	0.24	0.11	0.20	1.90	0.00	up	yes
ENSG00000130303	BST2	1.97	1.70	2.03	0.55	0.30	0.68	1.89	0.00	up	yes

ENSG00000009950	MLXIPL	0.29	0.42	0.40	0.16	0.05	0.09	1.89	0.00	up	yes
ENSG00000233030	AC243772	0.24	0.29	0.17	0	0.11	0.09	1.89	0.01	up	yes
ENSG00000175471	MCTP1	0.43	0.39	0.43	0.13	0.11	0.10	1.88	0.00	up	yes
ENSG00000232931	LINC00342	2.65	3.23	1.46	0.25	0.69	1.05	1.88	0.00	up	yes
ENSG00000114853	ZBTB47	0.84	0.85	0.94	0.24	0.21	0.26	1.88	0.00	up	yes
ENSG00000128872	TMOD2	0.46	0.61	0.62	0.15	0.17	0.15	1.88	0.00	up	yes
ENSG00000064270	ATP2C2	0.43	0.34	0.32	0.10	0.09	0.11	1.87	0.00	up	yes
ENSG00000120217	CD274	0.31	0.35	0.41	0.09	0.11	0.09	1.87	0.00	up	yes
ENSG00000162415	ZSWIM5	1.66	2.02	1.89	0.50	0.47	0.56	1.87	0.00	up	yes
ENSG00000105426	PTPRS	0.14	0.16	0.20	0.08	0.03	0.03	1.87	0.00	up	yes
ENSG00000169851	PCDH7	0.19	0.18	0.29	0.05	0.07	0.06	1.86	0.00	up	yes
ENSG00000171450	CDK5R2	0.28	0.38	0.45	0.19	0.07	0.06	1.84	0.00	up	yes
ENSG00000179104	TMTC2	0.99	1.01	0.92	0.25	0.22	0.35	1.84	0.00	up	yes
ENSG00000170522	ELOVL6	6.85	6.88	6.65	1.72	2.00	1.99	1.83	0.00	up	yes
ENSG00000269113	TRABD2B	2.68	2.75	2.70	0.84	0.68	0.77	1.83	0.00	up	yes
ENSG00000130600	H19	544.32	462.33	515.91	133.95	143.88	151.45	1.83	0.00	up	yes
ENSG00000160207	HSF2BP	2.61	1.89	2.27	0.51	0.67	0.73	1.83	0.00	up	yes
ENSG00000111344	RASAL1	0.87	1.14	1.03	0.29	0.28	0.29	1.82	0.00	up	yes
ENSG00000185269	NOTUM	119.66	119.52	132.66	33.83	36.19	35.03	1.82	0.00	up	yes
ENSG00000204264	PSMB8	5.72	5.49	6.38	1.61	2.04	1.32	1.82	0.00	up	yes
ENSG00000213997	PGAM1P7	2.13	0.34	1.31	0.31	0.34	0.43	1.80	0.00	up	yes
ENSG00000126583	PRKCG	0.60	0.54	0.66	0.19	0.23	0.10	1.80	0.00	up	yes
ENSG00000105137	SYDE1	0.40	0.71	0.83	0.14	0.23	0.18	1.80	0.00	up	yes
ENSG00000163590	PPM1L	0.53	0.52	0.39	0.10	0.16	0.16	1.80	0.00	up	yes
ENSG00000137877	SPTBN5	0.19	0.17	0.12	0.06	0.02	0.06	1.80	0.00	up	yes

ENSG00000182179	UBA7	0.24	0.10	0.22	0.06	0.08	0.02	1.80	0.00	up	yes
ENSG00000163586	FABP1	0.44	0.42	0.51	0.08	0.16	0.15	1.79	0.00	up	yes
ENSG00000188488	SERPINA5	1.03	1.22	1.08	0.33	0.33	0.29	1.79	0.00	up	yes
ENSG00000142875	PRKACB	2.58	2.42	2.20	0.59	0.73	0.78	1.78	0.00	up	yes
ENSG00000250510	GPR162	0.19	0.29	0.32	0.02	0.08	0.13	1.78	0.00	up	yes
ENSG00000168071	CCDC88B	0.17	0.36	0.20	0.07	0.04	0.10	1.78	0.00	up	yes
ENSG00000132141	CCT6B	0.26	0.28	0.18	0.03	0.05	0.13	1.78	0.00	up	yes
ENSG00000107984	DKK1	21.95	21.05	23.46	6.78	6.34	6.32	1.77	0.00	up	yes
ENSG00000161905	ALOX15	0.21	0.53	0.57	0.25	0.05	0.09	1.77	0.00	up	yes
ENSG00000165801	ARHGEF40	1.70	1.93	1.80	0.43	0.51	0.65	1.77	0.00	up	yes
ENSG00000184828	ZBTB7C	0.74	0.57	0.78	0.21	0.16	0.24	1.77	0.00	up	yes
ENSG00000157214	STEAP2	0.17	0.17	0.09	0.02	0.08	0.03	1.75	0.00	up	yes
ENSG00000147231	RADX	0.29	0.23	0.23	0.14	0.05	0.03	1.75	0.00	up	yes
ENSG00000128203	ASPHD2	0.22	0.43	0.61	0.12	0.09	0.16	1.75	0.00	up	yes
ENSG00000263155	MYZAP	1.93	2.02	2.72	0.73	0.65	0.61	1.74	0.00	up	yes
ENSG00000171992	SYNPO	24.62	23.09	22.67	7.00	6.76	7.32	1.74	0.00	up	yes
ENSG00000116962	NID1	5.13	5.74	5.53	1.34	1.74	1.84	1.74	0.00	up	yes
ENSG00000267924	AC139769	2.04	1.56	1.69	0.55	0.65	0.38	1.74	0.00	up	yes
ENSG00000177839	PCDHB9	0.24	0.15	0.29	0.05	0.12	0.03	1.74	0.00	up	yes
ENSG00000132535	DLG4	0.77	0.87	0.83	0.24	0.27	0.23	1.74	0.00	up	yes
ENSG00000182489	XKRX	0.41	0.54	0.34	0.09	0.13	0.17	1.73	0.00	up	yes
ENSG00000102879	CORO1A	0.26	0.12	0.25	0.10	0.07	0.02	1.73	0.00	up	yes
ENSG00000175920	DOK7	0.29	0.51	0.26	0.11	0.20	0.01	1.72	0.01	up	yes
ENSG00000139531	SUOX	1.92	5.36	4.55	0.81	0.86	1.91	1.72	0.00	up	yes
ENSG00000053747	LAMA3	21.42	20.24	20.95	6.57	6.01	6.41	1.72	0.00	up	yes

ENSG00000257556	LINC02298	1.02	1.24	1.13	0.45	0.12	0.45	1.72	0.00	up	yes
ENSG00000280693	SH3PXD2A-AS1	0.89	0.79	0.72	0.21	0.23	0.29	1.71	0.00	up	yes
ENSG00000198753	PLXNB3	1.54	1.58	1.53	0.51	0.56	0.35	1.71	0.00	up	yes
ENSG00000205413	SAMD9	0.56	0.70	0.52	0.22	0.13	0.20	1.71	0.00	up	yes
ENSG00000205420	KRT6A	0.92	0.92	0.74	0.19	0.20	0.40	1.71	0.00	up	yes
ENSG00000164342	TLR3	0.42	0.59	0.53	0.16	0.16	0.16	1.70	0.00	up	yes
ENSG00000151726	ACSL1	6.43	5.27	6.73	1.93	2.01	1.77	1.69	0.00	up	yes
ENSG00000175274	TP53I11	3.91	3.68	2.61	1.30	1.20	0.66	1.69	0.00	up	yes
ENSG00000103269	RHBDL1	0.53	0.21	0.48	0.11	0.06	0.21	1.69	0.00	up	yes
ENSG00000266714	MYO15B	0.60	0.73	0.63	0.17	0.23	0.21	1.69	0.00	up	yes
ENSG00000159403	C1R	1.03	0.98	1.25	0.38	0.29	0.35	1.69	0.00	up	yes
ENSG00000197054	ZNF763	0.50	0.69	1.05	0.42	0.04	0.23	1.68	0.01	up	yes
ENSG00000069535	MAOB	1.92	1.53	1.29	0.52	0.42	0.54	1.68	0.00	up	yes
ENSG00000164136	IL15	0.31	0.28	0.31	0.07	0.08	0.13	1.68	0.00	up	yes
ENSG00000157870	PRXL2B	3.46	9.89	2.28	1.61	1.54	1.74	1.68	0.00	up	yes
ENSG00000185499	MUC1	3.64	4.09	3.55	1.06	1.48	0.99	1.67	0.00	up	yes
ENSG00000198794	SCAMP5	2.58	0.63	2.69	0.64	0.57	0.65	1.67	0.00	up	yes
ENSG00000283782	AC116366	0.15	0.15	0.16	0.03	0.04	0.07	1.67	0.00	up	yes
ENSG00000188818	ZDHHC11	0.16	0.11	0.12	0.07	0.01	0.05	1.67	0.00	up	yes
ENSG00000010319	SEMA3G	0.49	0.39	0.47	0.11	0.18	0.13	1.66	0.00	up	yes
ENSG00000156587	UBE2L6	17.51	19.06	18.49	5.01	5.33	7.06	1.66	0.00	up	yes
ENSG00000179023	KLHDC7A	1.50	1.73	1.44	0.50	0.54	0.44	1.66	0.00	up	yes
ENSG00000250786	SNHG18	3.93	4.15	3.70	1.21	1.41	1.11	1.66	0.00	up	yes
ENSG00000151623	NR3C2	0.17	0.25	0.17	0.08	0.01	0.10	1.65	0.01	up	yes
ENSG00000084636	COL16A1	0.51	0.48	0.44	0.18	0.15	0.12	1.65	0.00	up	yes

ENSG00000188687	SLC4A5	0.11	0.12	0.11	0.02	0.05	0.03	1.65	0.00	up	yes
ENSG00000026950	BTN3A1	1.00	1.75	0.95	0.35	0.40	0.42	1.65	0.00	up	yes
ENSG00000112902	SEMA5A	1.15	1.53	1.29	0.40	0.47	0.40	1.65	0.00	up	yes
ENSG00000009724	MASP2	0.33	0.24	0.46	0.10	0.09	0.14	1.65	0.00	up	yes
ENSG00000111674	ENO2	0.21	0.05	0.19	0.09	0.04	0.02	1.65	0.02	up	yes
ENSG00000049192	ADAMTS6	0.19	0.26	0.17	0.07	0.08	0.05	1.65	0.00	up	yes
ENSG00000158163	DZIP1L	0.28	0.32	0.27	0.09	0.08	0.11	1.64	0.00	up	yes
ENSG00000155016	CYP2U1	0.81	1.04	0.96	0.26	0.38	0.27	1.63	0.00	up	yes
ENSG00000106723	SPIN1	6.14	23.62	23.23	4.91	6.18	6.00	1.63	0.00	up	yes
ENSG00000117226	GBP3	4.42	4.56	4.35	1.49	1.56	1.25	1.63	0.00	up	yes
ENSG00000133106	EPSTI1	5.35	5.56	4.11	1.72	1.63	1.50	1.63	0.00	up	yes
ENSG00000161405	IKZF3	0.74	0.88	0.14	0.16	0.24	0.17	1.63	0.00	up	yes
ENSG00000135472	FAIM2	0.19	0.15	0.15	0.04	0.04	0.07	1.62	0.00	up	yes
ENSG00000159753	CARMIL2	0.53	0.48	0.56	0.18	0.17	0.16	1.62	0.00	up	yes
ENSG00000125207	PIWIL1	0.54	0.53	0.50	0.15	0.08	0.27	1.62	0.00	up	yes
ENSG00000114854	TNNC1	7.84	9.10	8.18	3.01	2.82	2.36	1.62	0.00	up	yes
ENSG00000185432	METTL7A	2.21	2.68	2.69	0.86	0.84	0.77	1.62	0.00	up	yes
ENSG00000117707	PROX1	1.40	2.18	1.84	0.55	0.60	0.61	1.62	0.00	up	yes
ENSG00000198959	TGM2	18.98	19.18	20.91	6.46	6.35	6.47	1.61	0.00	up	yes
ENSG00000167880	EVPL	1.97	5.91	6.20	0.84	1.89	1.87	1.61	0.00	up	yes
ENSG00000060709	RIMBP2	0.11	0.15	0.13	0.06	0.03	0.04	1.61	0.00	up	yes
ENSG00000263740	RN7SL4P	22.15	22.46	31.78	10.86	6.40	7.75	1.61	0.00	up	yes
ENSG00000175294	CATSPER1	0.36	0.18	0.37	0.15	0.04	0.11	1.60	0.00	up	yes
ENSG00000082014	SMARCD3	2.36	2.42	2.25	0.86	0.73	0.72	1.60	0.00	up	yes
ENSG00000162576	MXRA8	1.13	1.21	0.90	0.37	0.31	0.39	1.60	0.00	up	yes

ENSG00000117643	MAN1C1	0.11	0.22	0.16	0.06	0.02	0.08	1.60	0.00	up	yes
ENSG00000091428	RAPGEF4	0.17	0.14	0.19	0.07	0.06	0.04	1.60	0.00	up	yes
ENSG00000188158	NHS	4.40	4.78	4.63	1.59	1.53	1.46	1.59	0.00	up	yes
ENSG00000237330	RNF223	1.17	0.92	1.19	0.26	0.46	0.37	1.59	0.00	up	yes
ENSG00000102362	SYTL4	0.89	0.89	0.96	0.42	0.13	0.36	1.59	0.00	up	yes
ENSG00000171747	LGALS4	2.69	2.68	3.78	0.87	1.06	1.12	1.58	0.00	up	yes
ENSG00000170271	FAXDC2	0.81	0.83	0.75	0.32	0.28	0.20	1.58	0.00	up	yes
ENSG00000286112	AL441992	0.23	1.10	0.16	0.21	0.11	0.18	1.58	0.02	up	yes
ENSG00000136367	ZFHX2	0.35	0.42	0.41	0.12	0.12	0.15	1.57	0.00	up	yes
ENSG00000160050	CCDC28B	1.14	1.34	1.51	0.59	0.51	0.24	1.57	0.00	up	yes
ENSG00000088543	C3orf18	1.02	1.28	1.20	0.47	0.28	0.42	1.57	0.00	up	yes
ENSG00000197506	SLC28A3	0.26	0.19	0.15	0.07	0.09	0.04	1.57	0.00	up	yes
ENSG00000072422	RHOBTB1	0.67	0.60	0.58	0.20	0.25	0.17	1.57	0.00	up	yes
ENSG00000135362	PRR5L	0.22	0.21	0.23	0.09	0.11	0.02	1.57	0.00	up	yes
ENSG00000286019	AC239811	5.48	5.11	6.94	2.50	3.07	0.34	1.57	0.01	up	yes
ENSG00000204956	PCDHGA1	0.27	0.24	0.17	0.02	0.13	0.07	1.57	0.01	up	yes
ENSG00000249572	AC034231	1.14	2.54	1.61	0.66	0.81	0.32	1.57	0.00	up	yes
ENSG00000181444	ZNF467	2.18	2.16	2.85	0.84	1.20	0.40	1.56	0.00	up	yes
ENSG00000100003	SEC14L2	1.43	1.19	1.26	0.38	0.42	0.51	1.56	0.00	up	yes
ENSG00000152527	PLEKHH2	0.67	1.22	0.69	0.20	0.27	0.41	1.56	0.00	up	yes
ENSG00000246465	AC138904	0.51	0.52	0.45	0.15	0.15	0.21	1.56	0.00	up	yes
ENSG00000143147	GPR161	0.53	0.67	1.22	0.27	0.32	0.24	1.55	0.00	up	yes
ENSG00000144452	ABCA12	0.16	0.25	0.26	0.05	0.12	0.06	1.55	0.00	up	yes
ENSG00000152076	CCDC74B	0.27	0.40	0.20	0.10	0.13	0.07	1.55	0.00	up	yes
ENSG00000234857	HNRNPUL2-BSCL2	2.05	1.62	1.36	0.75	0.48	0.49	1.54	0.00	up	yes

ENSG00000197385	ZNF860	3.63	1.90	2.24	0.37	1.21	1.09	1.54	0.00	up	yes
ENSG00000134107	BHLHE40	7.95	8.72	9.87	3.17	2.99	2.97	1.54	0.00	up	yes
ENSG00000181773	GPR3	0.66	0.77	0.64	0.09	0.36	0.26	1.54	0.00	up	yes
ENSG00000163704	PRRT3	0.38	0.42	0.34	0.12	0.14	0.14	1.53	0.00	up	yes
ENSG00000115902	SLC1A4	2.72	2.68	2.63	0.88	1.01	0.88	1.53	0.00	up	yes
ENSG00000116852	KIF21B	2.08	2.26	2.04	0.63	0.85	0.73	1.53	0.00	up	yes
ENSG00000088340	FER1L4	0.57	0.37	0.58	0.20	0.12	0.21	1.53	0.00	up	yes
ENSG00000115361	ACADL	0.36	0.53	0.33	0.11	0.12	0.19	1.53	0.00	up	yes
ENSG00000108679	LGALS3BP	35.85	34.86	36.63	12.86	12.12	12.14	1.53	0.00	up	yes
ENSG00000008441	NFIX	4.32	2.34	6.33	1.11	1.57	1.81	1.53	0.00	up	yes
ENSG00000205041	AC118344	0.63	0.88	0.63	0.17	0.34	0.23	1.53	0.00	up	yes
ENSG00000166974	MAPRE2	3.20	3.57	3.66	0.97	1.61	1.04	1.53	0.00	up	yes
ENSG00000248712	CCDC153	0.63	0.52	0.83	0.25	0.17	0.27	1.53	0.00	up	yes
ENSG00000159216	RUNX1	8.61	8.73	8.49	3.07	3.01	2.93	1.52	0.00	up	yes
ENSG00000120328	PCDHB12	0.50	0.39	0.35	0.15	0.18	0.11	1.52	0.00	up	yes
ENSG00000128422	KRT17	1.30	1.41	1.18	0.42	0.48	0.46	1.52	0.00	up	yes
ENSG00000108602	ALDH3A1	3.14	3.40	3.18	0.91	1.41	1.08	1.52	0.00	up	yes
ENSG00000186854	TRABD2A	2.57	2.27	2.13	0.86	0.75	0.83	1.52	0.00	up	yes
ENSG00000128604	IRF5	1.01	0.92	0.91	0.29	0.48	0.22	1.52	0.00	up	yes
ENSG00000123080	CDKN2C	2.03	1.71	2.95	0.63	1.07	0.65	1.51	0.00	up	yes
ENSG00000001617	SEMA3F	2.14	1.91	2.08	0.69	0.75	0.71	1.51	0.00	up	yes
ENSG00000260293	AC106820	0.27	0.31	0.17	0.10	0.08	0.08	1.51	0.00	up	yes
ENSG00000178977	LINC00324	0.52	0.40	0.56	0.12	0.21	0.18	1.50	0.00	up	yes
ENSG00000123612	ACVR1C	0.22	0.23	0.21	0.09	0.03	0.12	1.50	0.00	up	yes
ENSG00000183044	ABAT	2.10	2.11	2.13	0.71	0.82	0.71	1.50	0.00	up	yes

ENSG00000162572	SCNN1D	0.29	0.15	0.39	0.07	0.13	0.09	1.50	0.00	up	yes
ENSG00000106479	ZNF862	0.80	0.79	0.63	0.24	0.30	0.25	1.50	0.00	up	yes
ENSG00000177707	NECTIN3	0.92	0.74	0.70	0.28	0.23	0.33	1.50	0.00	up	yes
ENSG00000165152	TMEM246	4.02	3.98	4.60	1.60	1.47	1.40	1.49	0.00	up	yes
ENSG00000135439	AGAP2	0.44	0.31	0.31	0.11	0.11	0.15	1.49	0.00	up	yes
ENSG00000152503	TRIM36	1.07	1.16	1.13	0.33	0.42	0.45	1.49	0.00	up	yes
ENSG00000270641	TSIX	2.00	1.20	2.54	0.73	0.44	0.88	1.49	0.00	up	yes
ENSG00000204282	TNRC6C-AS1	0.64	0.71	0.99	0.16	0.29	0.39	1.49	0.00	up	yes
ENSG00000132170	PPARG	1.54	2.78	3.24	0.96	0.71	1.03	1.49	0.00	up	yes
ENSG00000112297	CRYBG1	0.16	0.14	0.17	0.04	0.07	0.06	1.48	0.00	up	yes
ENSG00000144455	SUMF1	4.46	4.65	4.88	1.77	1.49	1.75	1.48	0.00	up	yes
ENSG00000225871	AC245100	0.53	0.86	0.37	0.29	0.20	0.15	1.48	0.01	up	yes
ENSG00000158373	HIST1H2BD	9.74	8.92	8.97	3.03	3.64	3.26	1.48	0.00	up	yes
ENSG00000106069	CHN2	3.03	3.02	2.24	0.89	1.15	0.95	1.47	0.00	up	yes
ENSG00000226950	DANCR	9.69	9.37	3.04	2.58	2.78	2.63	1.47	0.00	up	yes
ENSG00000272668	AL590560	0.26	0.21	0.23	0.09	0.07	0.09	1.47	0.00	up	yes
ENSG00000182379	NXPH4	4.42	4.09	3.74	1.69	1.66	1.08	1.47	0.00	up	yes
ENSG00000070182	SPTB	0.42	0.43	0.37	0.15	0.11	0.19	1.46	0.00	up	yes
ENSG00000136235	GPNMB	0.19	0.16	0.17	0.07	0.07	0.05	1.46	0.00	up	yes
ENSG00000235934	AC007405	2.03	1.95	2.12	0.77	0.59	0.85	1.46	0.00	up	yes
ENSG00000197483	ZNF628	1.54	1.68	1.71	0.90	0.50	0.38	1.46	0.00	up	yes
ENSG00000139269	INHBE	0.53	0.24	0.37	0.10	0.10	0.22	1.46	0.01	up	yes
ENSG00000171522	PTGER4	0.51	0.44	0.33	0.19	0.14	0.14	1.46	0.00	up	yes
ENSG00000261324	AC010168	0.95	0.90	1.02	0.43	0.34	0.28	1.46	0.00	up	yes
ENSG00000027075	PRKCH	0.11	0.11	0.07	0.05	0.03	0.04	1.46	0.01	up	yes

ENSG00000262587	AC133552	1.18	0.61	0.77	0.42	0.06	0.45	1.45	0.02	up	yes
ENSG00000159433	STARD9	0.22	0.24	0.20	0.10	0.05	0.08	1.45	0.00	up	yes
ENSG00000225526	MKRN2OS	0.64	0.61	1.14	0.29	0.38	0.20	1.45	0.00	up	yes
ENSG00000141756	FKBP10	3.16	4.35	4.92	1.46	1.61	1.47	1.45	0.00	up	yes
ENSG00000280027	AC007342	1.83	2.31	1.34	0.54	0.83	0.64	1.45	0.00	up	yes
ENSG00000169403	PTAFR	1.05	1.28	1.08	0.44	0.43	0.38	1.45	0.00	up	yes
ENSG00000227619	AL391056	0.27	0.38	0.41	0.08	0.13	0.18	1.45	0.01	up	yes
ENSG00000168026	TTC21A	0.28	0.21	0.30	0.05	0.17	0.07	1.45	0.00	up	yes
ENSG00000012779	ALOX5	1.63	1.47	1.89	0.54	0.64	0.65	1.45	0.00	up	yes
ENSG00000204618	RNF39	2.87	2.69	2.89	0.96	1.04	1.10	1.44	0.00	up	yes
ENSG00000225138	SLC9A3-AS1	2.01	1.93	1.96	0.82	0.72	0.62	1.44	0.00	up	yes
ENSG00000172037	LAMB2	9.59	9.45	9.95	4.02	3.38	3.34	1.43	0.00	up	yes
ENSG00000156299	TIAM1	2.68	3.00	2.70	0.92	1.09	1.10	1.43	0.00	up	yes
ENSG00000171051	FPR1	1.58	1.29	1.37	0.30	0.85	0.43	1.43	0.00	up	yes
ENSG00000187013	C17orf82	0.44	0.80	0.64	0.17	0.33	0.20	1.43	0.01	up	yes
ENSG00000114857	NKTR	4.26	5.63	4.27	1.09	1.32	2.86	1.43	0.00	up	yes
ENSG00000016402	IL20RA	1.54	1.46	1.40	0.35	0.67	0.62	1.43	0.00	up	yes
ENSG00000064692	SNCAIP	1.61	2.07	2.20	0.63	0.65	0.92	1.42	0.00	up	yes
ENSG00000136379	ABHD17C	17.55	18.50	18.39	6.54	7.05	6.75	1.42	0.00	up	yes
ENSG00000239282	CASTOR1	1.31	1.48	1.06	0.63	0.23	0.58	1.42	0.00	up	yes
ENSG00000135114	OASL	9.30	9.16	7.35	3.31	2.99	3.40	1.41	0.00	up	yes
ENSG00000160323	ADAMTS13	0.31	0.37	0.40	0.09	0.12	0.20	1.41	0.00	up	yes
ENSG00000169282	KCNAB1	0.19	0.20	0.23	0.06	0.10	0.08	1.41	0.00	up	yes
ENSG00000072818	ACAP1	0.15	0.10	0.19	0.04	0.09	0.04	1.41	0.01	up	yes
ENSG00000167680	SEMA6B	1.38	1.00	1.39	0.44	0.46	0.51	1.41	0.00	up	yes

ENSG00000172215	CXCR6	0.50	0.78	0.47	0.31	0.16	0.19	1.41	0.00	up	yes
ENSG00000254004	ZNF260	2.31	12.76	4.86	2.35	3.01	2.16	1.41	0.00	up	yes
ENSG00000134321	RSAD2	3.69	3.50	3.81	1.24	1.51	1.41	1.40	0.00	up	yes
ENSG00000226067	LINC00623	0.86	0.76	1.14	0.39	0.17	0.49	1.40	0.00	up	yes
ENSG00000137965	IFI44	1.46	2.03	1.56	0.64	0.78	0.50	1.40	0.00	up	yes
ENSG00000127329	PTPRB	3.85	3.59	3.79	1.15	1.54	1.57	1.40	0.00	up	yes
ENSG00000116883	AL591845	0.40	0.62	0.51	0.14	0.19	0.25	1.40	0.01	up	yes
ENSG00000103089	FA2H	1.90	2.28	2.14	0.71	0.83	0.87	1.39	0.00	up	yes
ENSG00000148734	NPFFR1	0.15	0.15	0.08	0.06	0.02	0.06	1.39	0.01	up	yes
ENSG00000126945	HNRNPH2	101.06	102.86	99.06	8.63	14.18	92.90	1.39	0.02	up	yes
ENSG00000143502	SUSD4	5.67	5.96	5.69	2.17	2.20	2.24	1.39	0.00	up	yes
ENSG00000280213	UCKL1-AS1	0.26	0.29	0.20	0.05	0.12	0.12	1.38	0.01	up	yes
ENSG00000270164	LINC01480	0.81	0.50	0.57	0.34	0.22	0.16	1.38	0.01	up	yes
ENSG00000285244	DINOL	0.68	0.94	0.88	0.45	0.35	0.16	1.38	0.01	up	yes
ENSG00000164039	BDH2	4.16	4.36	3.90	1.68	1.72	1.38	1.38	0.00	up	yes
ENSG00000171174	RBKS	0.46	0.40	0.67	0.18	0.15	0.26	1.38	0.00	up	yes
ENSG00000267922	AC007785	3.52	4.93	5.09	1.70	1.85	1.66	1.38	0.00	up	yes
ENSG00000163121	NEURL3	5.03	4.83	4.89	1.54	2.02	2.13	1.38	0.00	up	yes
ENSG00000130844	ZNF331	0.43	0.60	0.15	0.13	0.22	0.09	1.37	0.01	up	yes
ENSG00000168394	TAP1	8.21	7.51	7.32	2.93	3.13	2.83	1.37	0.00	up	yes
ENSG00000254027	AC009902	1.24	1.08	1.41	0.73	0.27	0.44	1.37	0.01	up	yes
ENSG00000188042	ARL4C	4.52	4.72	4.63	1.90	2.01	1.46	1.37	0.00	up	yes
ENSG00000157388	CACNA1D	0.14	0.06	0.12	0.05	0.03	0.04	1.37	0.00	up	yes
ENSG00000119699	TGFB3	1.21	1.60	1.49	0.54	0.51	0.62	1.37	0.00	up	yes
ENSG00000165646	SLC18A2	0.17	0.41	0.31	0.13	0.10	0.11	1.37	0.01	up	yes

ENSG00000223612	AC241585	0.87	4.05	2.26	1.28	1.27	0.23	1.37	0.05	up	yes
ENSG00000173210	ABLIM3	1.82	2.06	2.02	0.82	0.80	0.67	1.37	0.00	up	yes
ENSG00000111331	OAS3	14.09	14.42	14.00	5.43	5.62	5.45	1.37	0.00	up	yes
ENSG00000272425	AC009902	0.44	0.84	0.37	0.26	0.22	0.17	1.36	0.01	up	yes
ENSG00000103056	SMPD3	0.09	0.14	0.12	0.05	0.05	0.03	1.36	0.01	up	yes
ENSG00000152078	TMEM56	1.59	1.87	1.71	0.56	0.66	0.79	1.36	0.00	up	yes
ENSG00000153246	PLA2R1	0.20	0.24	0.26	0.05	0.12	0.11	1.36	0.00	up	yes
ENSG00000153714	LURAP1L	1.36	1.36	1.53	0.41	0.59	0.66	1.36	0.00	up	yes
ENSG00000198133	TMEM229B	2.70	2.22	2.35	0.52	1.77	0.55	1.35	0.00	up	yes
ENSG00000180891	CUEDC1	2.77	2.91	3.08	1.43	1.00	1.00	1.35	0.00	up	yes
ENSG00000204174	NPY4R	0.86	0.96	0.64	0.39	0.19	0.38	1.35	0.00	up	yes
ENSG00000147642	SYBU	0.46	0.49	0.38	0.16	0.15	0.21	1.34	0.00	up	yes
ENSG00000050438	SLC4A8	1.13	1.09	1.23	0.52	0.41	0.44	1.34	0.00	up	yes
ENSG00000115423	DNAH6	0.24	0.26	0.28	0.08	0.12	0.11	1.34	0.00	up	yes
ENSG00000206127	GOLGA8O	0.42	0.64	0.50	0.24	0.25	0.13	1.34	0.01	up	yes
ENSG00000145476	CYP4V2	0.18	0.25	0.19	0.08	0.09	0.08	1.34	0.00	up	yes
ENSG00000253710	ALG11	7.91	2.34	6.82	2.15	1.68	2.92	1.34	0.00	up	yes
ENSG00000111801	BTN3A3	0.13	0.49	0.15	0.12	0.15	0.04	1.34	0.04	up	yes
ENSG00000075826	SEC31B	0.20	0.26	0.18	0.07	0.10	0.09	1.34	0.00	up	yes
ENSG00000064309	CDON	0.27	0.33	0.22	0.07	0.15	0.11	1.34	0.00	up	yes
ENSG00000086619	ERO1B	1.61	1.98	1.69	0.61	0.62	0.87	1.33	0.00	up	yes
ENSG00000204257	HLA-DMA	1.13	1.60	1.36	0.50	0.64	0.49	1.33	0.00	up	yes
ENSG00000144566	RAB5A	30.59	30.92	29.79	10.55	11.27	14.42	1.33	0.00	up	yes
ENSG00000165915	SLC39A13	3.45	1.88	3.69	1.06	1.38	1.14	1.33	0.00	up	yes
ENSG00000069812	HES2	4.54	4.91	4.48	3.90	0.73	0.91	1.33	0.01	up	yes

ENSG00000030419	IKZF2	0.35	0.90	0.81	0.18	0.45	0.19	1.33	0.00	up	yes
ENSG00000128655	PDE11A	0.43	0.46	0.39	0.20	0.12	0.19	1.33	0.00	up	yes
ENSG00000021300	PLEKHB1	3.48	4.15	3.66	1.40	1.57	1.53	1.33	0.00	up	yes
ENSG00000163629	PTPN13	0.62	0.47	0.60	0.20	0.18	0.29	1.33	0.00	up	yes
ENSG00000036672	USP2	0.34	0.44	0.43	0.18	0.08	0.22	1.33	0.01	up	yes
ENSG00000113448	PDE4D	0.46	0.49	0.40	0.19	0.21	0.14	1.33	0.00	up	yes
ENSG00000197467	COL13A1	0.35	0.37	0.42	0.14	0.10	0.21	1.32	0.00	up	yes
ENSG00000153294	ADGRF4	5.33	4.89	5.48	2.07	2.10	2.11	1.32	0.00	up	yes
ENSG00000275874	PICSAR	1.43	1.45	0.92	0.64	0.72	0.17	1.32	0.03	up	yes
ENSG00000109846	CRYAB	0.37	0.28	0.45	0.21	0.10	0.13	1.32	0.01	up	yes
ENSG00000134326	CMPK2	0.73	1.07	0.89	0.40	0.33	0.34	1.32	0.00	up	yes
ENSG00000187486	KCNJ11	0.96	1.44	1.08	0.37	0.55	0.48	1.32	0.00	up	yes
ENSG00000095932	SMIM24	7.21	8.28	7.09	3.36	2.77	2.95	1.32	0.00	up	yes
ENSG00000150764	DIXDC1	0.60	1.29	1.37	0.53	0.32	0.46	1.31	0.00	up	yes
ENSG00000140807	NKD1	25.15	25.58	25.37	10.62	9.86	10.14	1.31	0.00	up	yes
ENSG00000120322	PCDHB8	0.22	0.50	0.41	0.12	0.24	0.09	1.31	0.02	up	yes
ENSG00000079150	FKBP7	1.13	1.27	0.83	0.52	0.30	0.47	1.31	0.00	up	yes
ENSG00000269086	AC008555	0.26	0.70	0.50	0.17	0.24	0.18	1.31	0.00	up	yes
ENSG00000002726	AOC1	0.68	0.56	0.63	0.31	0.28	0.16	1.31	0.00	up	yes
ENSG00000204261	PSMB8-AS1	0.55	0.32	0.70	0.10	0.46	0.08	1.31	0.04	up	yes
ENSG00000159921	GNE	6.03	5.52	3.39	2.07	1.89	2.07	1.31	0.00	up	yes
ENSG00000135740	SLC9A5	0.73	0.78	0.74	0.32	0.24	0.35	1.31	0.00	up	yes
ENSG00000186862	PDZD7	0.24	0.17	0.30	0.06	0.13	0.10	1.31	0.01	up	yes
ENSG00000129038	LOXL1	0.24	0.18	0.31	0.15	0.07	0.08	1.31	0.01	up	yes
ENSG00000137393	RNF144B	1.12	1.29	1.21	0.48	0.46	0.53	1.30	0.00	up	yes

ENSG00000142611	PRDM16	0.16	0.12	0.17	0.05	0.09	0.05	1.30	0.01	up	yes
ENSG00000105696	TMEM59L	0.46	0.43	0.50	0.06	0.18	0.32	1.30	0.02	up	yes
ENSG00000128596	CCDC136	0.76	1.13	0.88	0.38	0.46	0.29	1.30	0.00	up	yes
ENSG00000196136	SERPINA3	0.84	0.68	0.62	0.26	0.30	0.30	1.30	0.00	up	yes
ENSG00000142920	AZIN2	0.12	0.13	0.15	0.05	0.07	0.04	1.30	0.00	up	yes
ENSG00000116117	PARD3B	0.90	0.85	0.93	0.34	0.28	0.47	1.30	0.00	up	yes
ENSG00000188322	SBK1	0.74	1.05	0.95	0.40	0.36	0.36	1.29	0.00	up	yes
ENSG00000114480	GBE1	4.33	4.16	3.92	1.82	1.67	1.58	1.29	0.00	up	yes
ENSG00000143479	DYRK3	1.01	1.09	1.28	0.33	0.52	0.53	1.29	0.00	up	yes
ENSG00000089225	TBX5	0.30	0.16	0.23	0.09	0.07	0.12	1.29	0.01	up	yes
ENSG00000196139	AKR1C3	0.88	0.96	0.64	0.41	0.39	0.21	1.29	0.00	up	yes
ENSG00000165591	FAAH2	2.38	2.05	2.09	1.01	0.68	0.98	1.29	0.00	up	yes
ENSG00000111424	VDR	1.44	1.75	1.11	0.50	0.70	0.56	1.29	0.00	up	yes
ENSG00000184260	HIST2H2AC	2.24	2.42	2.09	0.74	1.43	0.60	1.29	0.01	up	yes
ENSG00000234432	AC092171	1.17	1.15	1.18	0.36	0.66	0.42	1.29	0.00	up	yes
ENSG00000111962	UST	2.14	2.53	2.57	1.11	0.82	1.03	1.29	0.00	up	yes
ENSG00000286214	AUXG01000058	0.08	0.36	0.17	0.08	0.08	0.08	1.29	0.02	up	yes
ENSG00000164308	ERAP2	16.32	16.24	15.47	6.51	6.39	6.85	1.28	0.00	up	yes
ENSG00000142449	FBN3	0.29	0.40	0.25	0.14	0.09	0.16	1.28	0.00	up	yes
ENSG00000184441	AP001062	0.33	0.61	0.35	0.35	0.09	0.09	1.28	0.03	up	yes
ENSG00000279530	AC092881	0.44	0.50	0.55	0.16	0.25	0.21	1.28	0.00	up	yes
ENSG00000173898	SPTBN2	8.17	8.20	9.11	3.51	3.54	3.45	1.28	0.00	up	yes
ENSG00000189120	SP6	1.91	2.37	2.13	0.86	0.78	1.00	1.28	0.00	up	yes
ENSG00000170017	ALCAM	7.44	6.71	6.92	3.10	2.80	2.78	1.28	0.00	up	yes
ENSG00000146555	SDK1	0.54	0.58	0.55	0.21	0.25	0.22	1.28	0.00	up	yes

ENSG00000204362	AL590644	0.47	0.60	0.53	0.14	0.39	0.12	1.28	0.01	up	yes
ENSG00000178826	TMEM139	2.10	4.15	2.32	0.54	1.41	1.58	1.28	0.00	up	yes
ENSG00000143126	CELSR2	3.06	3.25	3.13	1.22	1.35	1.32	1.28	0.00	up	yes
ENSG00000184678	HIST2H2BE	2.52	2.29	2.20	1.06	1.06	0.78	1.28	0.00	up	yes
ENSG00000122417	ODF2L	1.12	1.15	1.01	0.43	0.48	0.45	1.27	0.00	up	yes
ENSG00000182580	EPHB3	3.86	3.12	4.33	1.55	1.56	1.57	1.27	0.00	up	yes
ENSG00000253361	AC069120	0.77	0.63	0.71	0.35	0.21	0.31	1.27	0.00	up	yes
ENSG00000079101	CLUL1	0.33	0.19	0.29	0.08	0.12	0.14	1.27	0.01	up	yes
ENSG00000169169	CPT1C	2.30	2.17	2.58	1.19	0.93	0.80	1.27	0.00	up	yes
ENSG00000071575	TRIB2	8.86	9.21	8.85	3.70	3.78	3.68	1.27	0.00	up	yes
ENSG00000100342	APOL1	3.48	0.99	3.01	1.62	1.08	0.39	1.27	0.01	up	yes
ENSG00000086548	CEACAM6	7.62	7.91	7.82	3.63	3.14	2.92	1.27	0.00	up	yes
ENSG00000170786	SDR16C5	0.71	0.63	0.78	0.27	0.37	0.24	1.27	0.00	up	yes
ENSG00000213033	AURKAP1	1.35	1.04	0.96	0.62	0.29	0.48	1.26	0.01	up	yes
ENSG00000145362	ANK2	0.21	0.18	0.20	0.10	0.10	0.05	1.26	0.00	up	yes
ENSG00000178252	WDR6	17.12	6.78	18.01	4.07	3.24	10.19	1.26	0.00	up	yes
ENSG00000214376	VSTM5	0.31	0.32	0.24	0.15	0.04	0.18	1.26	0.03	up	yes
ENSG00000139597	N4BP2L1	0.60	0.56	0.63	0.33	0.19	0.23	1.26	0.00	up	yes
ENSG00000179148	ALOXE3	0.45	0.65	0.50	0.27	0.19	0.21	1.26	0.00	up	yes
ENSG00000259488	AC023355	0.19	0.32	0.26	0.11	0.14	0.07	1.26	0.01	up	yes
ENSG00000065320	NTN1	3.58	3.67	4.10	1.62	1.64	1.49	1.26	0.00	up	yes
ENSG00000185730	ZNF696	2.36	0.39	0.53	0.48	0.54	0.35	1.26	0.04	up	yes
ENSG00000198908	BHLHB9	4.27	4.86	0.92	0.78	2.24	1.19	1.25	0.02	up	yes
ENSG00000083814	ZNF671	0.37	0.36	0.25	0.17	0.16	0.09	1.25	0.01	up	yes
ENSG00000091622	PITPNM3	0.46	0.60	0.51	0.16	0.23	0.26	1.25	0.00	up	yes

ENSG00000123560	PLP1	0.99	1.38	1.79	0.52	0.76	0.47	1.25	0.00	up	yes
ENSG00000197415	VEPH1	0.20	0.19	0.29	0.12	0.04	0.12	1.25	0.01	up	yes
ENSG00000225205	AC078883	0.99	1.28	1.08	0.51	0.38	0.52	1.25	0.00	up	yes
ENSG00000164850	GPER1	2.28	4.40	4.03	1.93	1.71	0.88	1.25	0.00	up	yes
ENSG00000261857	MIA	2.40	3.18	4.65	1.70	0.72	1.90	1.24	0.01	up	yes
ENSG00000279569	AC020763	0.41	0.61	0.87	0.32	0.29	0.19	1.24	0.00	up	yes
ENSG00000104888	SLC17A7	0.30	0.21	0.19	0.10	0.13	0.06	1.24	0.01	up	yes
ENSG00000069011	PITX1	18.06	19.41	19.56	8.61	7.01	8.53	1.24	0.00	up	yes
ENSG00000168016	TRANK1	1.49	1.58	1.56	0.64	0.65	0.68	1.24	0.00	up	yes
ENSG00000272899	ATP6V1FNB	0.98	1.16	1.01	0.42	0.57	0.34	1.24	0.00	up	yes
ENSG00000185386	MAPK11	1.31	1.54	1.25	0.48	0.46	0.80	1.24	0.00	up	yes
ENSG00000167535	CACNB3	1.51	1.85	1.55	0.75	0.62	0.72	1.23	0.00	up	yes
ENSG00000089335	ZNF302	5.77	14.87	5.50	3.48	3.74	3.90	1.23	0.00	up	yes
ENSG00000133131	MORC4	27.49	28.19	26.35	10.91	11.67	12.36	1.23	0.00	up	yes
ENSG00000079337	RAPGEF3	0.39	0.40	0.50	0.22	0.14	0.20	1.23	0.00	up	yes
ENSG00000101115	SALL4	1.42	1.40	1.37	0.56	0.46	0.76	1.23	0.00	up	yes
ENSG00000188761	BCL2L15	3.27	2.73	4.24	1.43	1.28	1.66	1.23	0.00	up	yes
ENSG00000143590	EFNA3	8.96	7.83	8.54	3.70	3.33	3.77	1.23	0.00	up	yes
ENSG00000161381	PLXDC1	0.08	0.18	0.19	0.06	0.04	0.10	1.23	0.02	up	yes
ENSG00000134864	GGACT	0.36	0.28	0.23	0.08	0.19	0.11	1.23	0.02	up	yes
ENSG00000145103	ILDR1	0.54	0.51	0.73	0.16	0.21	0.39	1.23	0.01	up	yes
ENSG00000243566	UPK3B	0.63	0.79	1.03	0.28	0.41	0.35	1.23	0.00	up	yes
ENSG00000153551	CMTM7	5.95	6.11	3.51	2.11	2.12	2.42	1.23	0.00	up	yes
ENSG00000142623	PADI1	7.04	7.08	6.95	3.12	3.07	2.81	1.23	0.00	up	yes
ENSG00000187474	FPR3	1.16	1.28	1.13	0.61	0.47	0.45	1.23	0.00	up	yes

ENSG00000177432	NAP1L5	5.29	4.77	4.81	1.95	1.99	2.42	1.22	0.00	up	yes
ENSG00000010310	GIPR	0.69	1.24	0.70	0.40	0.38	0.35	1.22	0.00	up	yes
ENSG00000136153	LMO7	2.83	3.07	2.59	1.22	1.34	1.08	1.22	0.00	up	yes
ENSG00000205336	ADGRG1	20.34	20.03	20.57	9.23	8.56	8.34	1.22	0.00	up	yes
ENSG00000160932	LY6E	37.92	35.08	63.06	14.76	23.20	20.42	1.22	0.00	up	yes
ENSG00000204947	ZNF425	0.43	0.64	0.53	0.20	0.25	0.24	1.22	0.00	up	yes
ENSG00000158457	TSPAN33	0.99	0.83	1.09	0.30	0.50	0.44	1.22	0.00	up	yes
ENSG00000263528	IKBKE	4.23	4.48	4.92	1.85	2.22	1.78	1.22	0.00	up	yes
ENSG00000104081	BMF	8.30	9.16	9.28	3.84	3.62	4.03	1.22	0.00	up	yes
ENSG00000120324	PCDHB10	0.65	0.25	0.57	0.15	0.27	0.21	1.22	0.01	up	yes
ENSG00000168907	PLA2G4F	0.44	0.54	0.55	0.23	0.25	0.17	1.22	0.00	up	yes
ENSG00000213420	GPC2	0.26	0.20	0.30	0.11	0.05	0.16	1.22	0.02	up	yes
ENSG00000149043	SYT8	0.18	0.14	0.26	0.08	0.08	0.10	1.22	0.02	up	yes
ENSG00000128512	DOCK4	0.41	0.51	0.45	0.24	0.16	0.18	1.22	0.00	up	yes
ENSG00000114737	CISH	1.19	1.41	1.14	0.63	0.55	0.44	1.22	0.00	up	yes
ENSG00000187837	HIST1H1C	14.53	13.89	15.35	6.48	6.19	6.19	1.21	0.00	up	yes
ENSG00000065621	GSTO2	1.15	1.02	1.07	0.46	0.34	0.60	1.21	0.00	up	yes
ENSG00000064393	HIPK2	5.11	5.20	4.37	2.20	2.16	1.98	1.21	0.00	up	yes
ENSG00000214021	TTLL3	0.45	0.37	0.47	0.18	0.15	0.23	1.21	0.00	up	yes
ENSG00000277806	AC006213	1.57	1.41	1.33	0.83	0.31	0.72	1.21	0.02	up	yes
ENSG00000123191	ATP7B	3.57	2.65	3.48	1.38	1.44	1.37	1.21	0.00	up	yes
ENSG00000214944	ARHGEF28	1.04	0.98	0.93	0.42	0.41	0.45	1.21	0.00	up	yes
ENSG00000167755	KLK6	15.56	16.13	16.42	7.07	6.10	7.68	1.21	0.00	up	yes
ENSG00000074370	ATP2A3	0.52	0.57	0.69	0.26	0.21	0.30	1.20	0.00	up	yes
ENSG00000228492	RAB11FIP1P1	0.82	1.20	0.73	0.61	0.26	0.33	1.20	0.01	up	yes

ENSG00000165995	CACNB2	0.11	0.11	0.11	0.03	0.04	0.07	1.20	0.01	up	yes
ENSG00000010361	FUZ	0.54	0.64	0.56	0.36	0.20	0.20	1.20	0.01	up	yes
ENSG00000272419	LINC01145	0.72	1.07	0.81	0.33	0.42	0.37	1.20	0.00	up	yes
ENSG00000142961	MOB3C	1.22	0.70	0.92	0.32	0.47	0.45	1.20	0.00	up	yes
ENSG00000163328	GPR155	0.38	0.48	0.26	0.17	0.18	0.13	1.20	0.00	up	yes
ENSG00000227124	ZNF717	0.97	0.92	0.92	0.37	0.38	0.48	1.20	0.00	up	yes
ENSG00000063180	CA11	11.38	9.40	12.55	5.02	4.85	4.64	1.20	0.00	up	yes
ENSG00000088992	TESC	46.01	42.01	45.94	22.02	17.88	18.50	1.20	0.00	up	yes
ENSG00000212127	TAS2R14	0.69	0.46	0.25	0.15	0.25	0.22	1.19	0.03	up	yes
ENSG00000109061	MYH1	0.38	0.23	0.46	0.17	0.15	0.14	1.19	0.01	up	yes
ENSG00000184979	USP18	5.91	6.98	5.85	2.72	2.39	3.12	1.19	0.00	up	yes
ENSG00000169239	CA5B	1.26	0.95	1.08	0.39	0.54	0.52	1.19	0.00	up	yes
ENSG00000213468	FIRRE	1.35	1.25	1.25	0.70	0.45	0.55	1.19	0.00	up	yes
ENSG00000108821	COL1A1	1.40	1.61	1.65	0.68	0.74	0.63	1.19	0.00	up	yes
ENSG00000140853	NLRC5	1.14	1.44	1.19	0.59	0.55	0.52	1.19	0.00	up	yes
ENSG00000135697	BCO1	0.34	0.44	0.33	0.24	0.07	0.17	1.18	0.03	up	yes
ENSG00000197535	MYO5A	1.02	1.00	0.96	0.44	0.46	0.42	1.18	0.00	up	yes
ENSG00000174721	FGFBP3	0.65	0.61	0.47	0.29	0.36	0.11	1.18	0.02	up	yes
ENSG00000151090	THRB	1.46	1.55	1.34	0.76	0.68	0.47	1.18	0.00	up	yes
ENSG00000168874	ATOH8	0.12	0.08	0.12	0.05	0.05	0.04	1.18	0.02	up	yes
ENSG00000197479	PCDHB11	0.62	0.56	0.59	0.19	0.33	0.26	1.18	0.00	up	yes
ENSG00000125148	MT2A	15.69	16.39	16.59	7.54	6.73	7.29	1.17	0.00	up	yes
ENSG00000131620	ANO1	6.18	5.57	6.30	2.88	2.75	2.38	1.17	0.00	up	yes
ENSG00000134569	LRP4	3.69	3.77	3.44	1.87	1.35	1.62	1.17	0.00	up	yes
ENSG00000128683	GAD1	9.97	10.87	11.47	4.34	4.86	5.14	1.17	0.00	up	yes

ENSG00000101333	PLCB4	7.84	9.11	7.36	3.53	3.94	3.33	1.17	0.00	up	yes
ENSG00000115267	IFIH1	6.01	5.60	6.38	2.68	2.51	2.81	1.17	0.00	up	yes
ENSG00000134138	MEIS2	0.79	0.85	0.76	0.34	0.38	0.36	1.17	0.00	up	yes
ENSG00000011677	GABRA3	3.33	3.62	3.57	1.64	1.52	1.52	1.17	0.00	up	yes
ENSG00000162458	FBLIM1	5.84	4.68	10.52	3.23	3.31	2.83	1.17	0.00	up	yes
ENSG00000197774	EME2	1.13	1.58	1.01	0.42	0.54	0.70	1.17	0.00	up	yes
ENSG00000205426	KRT81	1.21	1.59	0.83	0.60	0.55	0.46	1.17	0.01	up	yes
ENSG00000101276	SLC52A3	6.06	6.45	5.43	2.72	2.52	2.77	1.16	0.00	up	yes
ENSG00000169696	ASPSCR1	3.31	3.31	3.45	1.60	1.46	1.43	1.16	0.00	up	yes
ENSG00000076770	MBNL3	7.41	7.75	7.56	3.27	3.25	3.62	1.16	0.00	up	yes
ENSG00000165886	UBTD1	8.78	7.63	9.31	3.86	3.86	3.76	1.16	0.00	up	yes
ENSG00000005471	ABCB4	0.14	0.11	0.15	0.02	0.08	0.08	1.16	0.03	up	yes
ENSG00000130768	SMPDL3B	9.66	10.09	10.83	5.25	4.29	4.16	1.16	0.00	up	yes
ENSG00000281189	GHET1	0.97	0.81	0.62	0.39	0.29	0.40	1.16	0.01	up	yes
ENSG00000104808	DHDH	1.57	1.55	1.47	0.83	0.41	0.81	1.16	0.01	up	yes
ENSG00000071242	RPS6KA2	0.63	0.71	0.57	0.18	0.37	0.31	1.16	0.00	up	yes
ENSG00000187210	GCNT1	0.89	0.71	0.91	0.48	0.53	0.11	1.16	0.01	up	yes
ENSG00000188760	TMEM198	0.41	0.47	0.36	0.16	0.19	0.21	1.16	0.01	up	yes
ENSG00000162591	MEGF6	0.40	0.39	0.44	0.17	0.16	0.23	1.15	0.00	up	yes
ENSG00000104213	PDGFRL	2.45	2.51	2.26	1.11	1.28	0.86	1.15	0.00	up	yes
ENSG00000135407	AVIL	0.29	0.38	0.27	0.18	0.07	0.18	1.15	0.02	up	yes
ENSG00000168389	MFSD2A	2.18	1.93	2.26	1.13	0.71	1.02	1.15	0.00	up	yes
ENSG00000187994	RINL	0.67	0.54	0.41	0.21	0.29	0.24	1.15	0.01	up	yes
ENSG00000118762	PKD2	7.44	7.50	7.58	3.34	3.60	3.23	1.15	0.00	up	yes
ENSG00000100311	PDGFB	4.84	4.75	5.08	1.22	2.55	2.84	1.15	0.00	up	yes

ENSG00000224660	SH3BP5-AS1	1.79	2.15	1.40	0.92	0.50	0.99	1.15	0.00	up	yes
ENSG00000140398	NEIL1	0.79	1.14	0.59	0.44	0.31	0.39	1.14	0.00	up	yes
ENSG00000163947	ARHGEF3	4.06	5.21	5.55	2.36	2.17	2.17	1.14	0.00	up	yes
ENSG00000176428	VPS37D	0.94	1.33	1.46	0.58	0.50	0.62	1.14	0.01	up	yes
ENSG00000144711	IQSEC1	2.07	2.94	2.26	1.15	0.98	1.16	1.14	0.00	up	yes
ENSG00000168234	TTC39C	1.59	1.83	1.80	0.96	0.43	0.99	1.14	0.00	up	yes
ENSG00000136603	SKIL	4.51	4.07	5.87	2.31	2.17	2.08	1.14	0.00	up	yes
ENSG00000280109	PLAC4	0.07	0.09	0.10	0.07	0.02	0.03	1.14	0.04	up	yes
ENSG00000067191	CACNB1	0.86	1.05	1.08	0.43	0.45	0.48	1.14	0.00	up	yes
ENSG00000100968	NFATC4	0.50	0.30	0.42	0.23	0.18	0.14	1.14	0.00	up	yes
ENSG00000174808	BTC	1.96	1.44	1.46	0.94	0.76	0.52	1.13	0.00	up	yes
ENSG00000173531	MST1	0.23	0.31	0.34	0.10	0.09	0.22	1.13	0.03	up	yes
ENSG00000198417	MT1F	0.72	0.64	0.85	0.38	0.29	0.34	1.13	0.01	up	yes
ENSG00000183742	MACC1	3.72	4.27	3.88	1.81	1.90	1.70	1.13	0.00	up	yes
ENSG00000149260	CAPN5	0.57	0.95	0.92	0.29	0.43	0.39	1.13	0.00	up	yes
ENSG00000243696	AC006254	0.18	0.27	0.13	0.07	0.08	0.12	1.13	0.03	up	yes
ENSG00000149809	TM7SF2	10.01	11.46	11.28	4.83	4.77	5.37	1.13	0.00	up	yes
ENSG00000204175	GPRIN2	5.31	4.63	5.23	3.12	2.41	1.41	1.13	0.00	up	yes
ENSG00000154127	UBASH3B	2.33	2.50	2.21	0.97	1.20	1.05	1.13	0.00	up	yes
ENSG00000137936	BCAR3	1.14	1.20	1.42	0.64	0.52	0.57	1.13	0.00	up	yes
ENSG00000010219	DYRK4	0.38	0.37	0.36	0.16	0.15	0.20	1.12	0.00	up	yes
ENSG00000164855	TMEM184A	2.04	3.43	3.28	1.43	1.50	1.10	1.12	0.00	up	yes
ENSG00000254739	AC137894	3.18	3.73	4.44	1.55	1.60	2.06	1.12	0.01	up	yes
ENSG00000124097	HMGB1P1	3.22	1.15	1.37	1.05	0.73	0.86	1.12	0.04	up	yes
ENSG00000131669	NINJ1	13.33	12.91	13.20	4.30	7.16	6.69	1.12	0.00	up	yes

ENSG00000196268	ZNF493	0.48	0.63	1.12	0.18	0.47	0.37	1.12	0.01	up	yes
ENSG00000223572	CKMT1A	4.29	3.18	2.78	1.57	1.80	1.36	1.12	0.00	up	yes
ENSG00000279400	AC008957	1.74	2.88	1.95	1.01	0.97	1.05	1.12	0.00	up	yes
ENSG00000123407	HOXC12	0.74	0.71	0.62	0.44	0.30	0.22	1.12	0.01	up	yes
ENSG00000137460	FHDC1	2.46	2.35	2.58	1.17	0.98	1.26	1.11	0.00	up	yes
ENSG00000142619	PADI3	2.35	2.48	2.48	1.25	1.06	1.07	1.11	0.00	up	yes
ENSG00000164638	SLC29A4	0.57	0.38	0.60	0.19	0.35	0.19	1.11	0.01	up	yes
ENSG00000041880	PARP3	1.37	1.72	2.10	1.07	0.59	0.74	1.11	0.00	up	yes
ENSG00000167723	TRPV3	0.21	0.21	0.21	0.07	0.15	0.07	1.11	0.02	up	yes
ENSG00000168297	PXK	2.28	1.99	2.19	1.27	0.88	0.85	1.11	0.00	up	yes
ENSG00000173083	HPSE	3.06	3.13	2.96	1.39	1.33	1.53	1.11	0.00	up	yes
ENSG00000178537	SLC25A20	9.47	9.36	10.27	4.84	3.64	5.05	1.10	0.00	up	yes
ENSG00000171574	ZNF584	1.69	1.99	5.45	1.48	1.37	1.40	1.10	0.01	up	yes
ENSG00000259623	AC125257	0.94	1.94	2.41	0.52	1.17	0.78	1.10	0.01	up	yes
ENSG00000124098	FAM210B	18.52	17.14	17.63	7.82	8.10	8.96	1.10	0.00	up	yes
ENSG00000204604	ZNF468	15.45	16.80	15.40	1.90	13.08	7.27	1.10	0.02	up	yes
ENSG00000159640	ACE	0.32	0.34	0.53	0.18	0.25	0.13	1.10	0.01	up	yes
ENSG00000215018	COL28A1	0.61	0.60	0.54	0.22	0.25	0.35	1.10	0.00	up	yes
ENSG00000214425	LRRC37A4P	2.88	3.36	2.66	1.59	1.19	1.38	1.10	0.00	up	yes
ENSG00000205464	ATP6AP1L	0.19	0.20	0.15	0.10	0.06	0.09	1.09	0.01	up	yes
ENSG00000138735	PDE5A	0.91	1.03	1.02	0.53	0.44	0.41	1.09	0.00	up	yes
ENSG00000129437	KLK14	0.46	0.73	0.98	0.27	0.45	0.30	1.09	0.03	up	yes
ENSG00000261754	AC008555	0.54	0.40	0.56	0.36	0.25	0.10	1.09	0.04	up	yes
ENSG00000142156	COL6A1	15.96	16.18	17.47	7.87	7.80	7.59	1.09	0.00	up	yes
ENSG00000171223	JUNB	15.68	15.64	17.41	7.92	7.74	7.20	1.09	0.00	up	yes

ENSG00000232300	FAM215B	0.56	0.75	0.46	0.30	0.31	0.22	1.09	0.02	up	yes
ENSG00000104325	DECR1	1.92	1.99	2.33	1.06	1.01	0.86	1.09	0.00	up	yes
ENSG00000197182	MIRLET7BHG	1.82	1.93	1.92	0.90	0.91	0.86	1.09	0.00	up	yes
ENSG00000233822	HIST1H2BN	0.14	0.25	0.28	0.11	0.08	0.12	1.08	0.02	up	yes
ENSG00000154146	NRGN	5.28	4.30	5.50	2.69	2.34	2.09	1.08	0.00	up	yes
ENSG00000189410	SH2D5	1.06	1.07	0.99	0.60	0.51	0.37	1.08	0.00	up	yes
ENSG00000187193	MT1X	2.73	1.97	2.63	1.09	1.16	1.22	1.08	0.00	up	yes
ENSG00000127152	BCL11B	0.55	0.64	0.61	0.21	0.25	0.40	1.08	0.00	up	yes
ENSG00000101017	CD40	4.53	4.64	4.45	2.17	2.45	1.83	1.08	0.00	up	yes
ENSG00000227212	PFN1P6	1.72	3.75	2.47	1.47	1.38	0.92	1.08	0.03	up	yes
ENSG00000204267	TAP2	6.65	6.76	6.95	3.24	3.40	3.03	1.07	0.00	up	yes
ENSG00000111859	NEDD9	8.35	8.81	8.44	4.10	4.29	3.78	1.07	0.00	up	yes
ENSG00000162407	PLPP3	5.10	4.81	4.75	2.10	2.34	2.54	1.07	0.00	up	yes
ENSG00000103044	HAS3	2.08	1.34	2.63	0.68	0.54	1.66	1.07	0.01	up	yes
ENSG00000158286	RNF207	0.23	0.29	0.19	0.12	0.12	0.10	1.07	0.01	up	yes
ENSG00000279806	AC018629	14.43	12.56	12.71	5.62	7.30	6.03	1.07	0.00	up	yes
ENSG00000100360	IFT27	1.27	1.39	2.38	0.83	0.78	0.80	1.07	0.00	up	yes
ENSG00000164663	USP49	1.30	1.20	1.05	1.04	0.35	0.30	1.07	0.01	up	yes
ENSG00000105929	ATP6V0A4	0.43	0.37	0.46	0.15	0.26	0.18	1.06	0.01	up	yes
ENSG00000108984	MAP2K6	0.61	0.67	0.60	0.33	0.29	0.28	1.06	0.00	up	yes
ENSG00000230316	FEZF1-AS1	0.33	0.34	0.27	0.14	0.12	0.19	1.06	0.03	up	yes
ENSG00000099953	MMP11	0.26	0.29	0.23	0.20	0.06	0.12	1.06	0.04	up	yes
ENSG00000108960	MMD	13.83	13.13	13.99	8.65	7.79	3.17	1.06	0.00	up	yes
ENSG00000273802	HIST1H2BG	1.74	1.21	1.45	0.67	0.86	0.58	1.06	0.01	up	yes
ENSG00000179630	LACC1	2.75	2.61	2.41	0.70	1.43	1.58	1.06	0.00	up	yes

ENSG00000091262	ABCC6	0.40	0.44	0.43	0.22	0.20	0.19	1.06	0.00	up	yes
ENSG00000173762	CD7	0.43	0.31	0.40	0.12	0.26	0.17	1.06	0.03	up	yes
ENSG00000105755	ETHE1	16.32	14.62	15.98	7.85	7.15	7.50	1.06	0.00	up	yes
ENSG00000157322	CLEC18A	0.11	0.15	0.14	0.08	0.06	0.05	1.06	0.03	up	yes
ENSG00000245025	AC107959	0.60	0.91	0.42	0.27	0.39	0.26	1.06	0.03	up	yes
ENSG00000071282	LMCD1	0.35	0.40	0.44	0.16	0.19	0.22	1.06	0.00	up	yes
ENSG00000280332	AC020917	0.45	0.65	0.32	0.28	0.25	0.16	1.06	0.05	up	yes
ENSG00000135373	EHF	8.92	9.53	6.41	3.77	4.11	4.08	1.06	0.00	up	yes
ENSG00000075643	MOCOS	1.46	1.55	1.43	0.83	0.56	0.75	1.05	0.00	up	yes
ENSG00000110693	SOX6	0.09	0.08	0.08	0.05	0.05	0.02	1.05	0.02	up	yes
ENSG00000054219	LY75	0.30	0.25	0.28	0.12	0.10	0.18	1.05	0.01	up	yes
ENSG00000126603	GLIS2	2.13	1.83	1.94	1.06	1.01	0.77	1.05	0.00	up	yes
ENSG00000130433	CACNG6	2.85	2.23	2.80	1.17	1.28	1.36	1.05	0.00	up	yes
ENSG00000138758	SEPT11	12.15	17.30	13.25	4.11	4.85	11.65	1.05	0.00	up	yes
ENSG00000229891	LINC01315	1.99	2.34	2.99	1.25	1.19	1.10	1.05	0.01	up	yes
ENSG00000180596	HIST1H2BC	1.39	1.97	1.24	1.20	0.51	0.51	1.05	0.04	up	yes
ENSG00000203811	HIST2H3C	1.10	0.84	0.51	0.43	0.40	0.34	1.05	0.03	up	yes
ENSG00000231439	WASIR2	1.75	1.19	1.08	0.73	0.53	0.68	1.05	0.02	up	yes
ENSG00000172296	SPTLC3	1.45	1.46	1.59	0.67	0.77	0.74	1.05	0.00	up	yes
ENSG00000270276	HIST2H4B	4.43	4.51	4.77	2.25	2.22	2.15	1.05	0.00	up	yes
ENSG00000254231	AC103760	0.50	0.43	0.58	0.21	0.17	0.35	1.05	0.02	up	yes
ENSG00000178201	VN1R1	1.36	1.97	1.55	0.71	0.76	0.88	1.05	0.00	up	yes
ENSG00000206337	HCP5	2.78	2.90	3.15	1.65	1.24	1.39	1.05	0.00	up	yes
ENSG00000166839	ANKDD1A	0.31	0.49	0.30	0.16	0.13	0.25	1.04	0.02	up	yes
ENSG00000203812	HIST2H2AA3	5.20	6.22	4.64	3.06	2.04	2.68	1.04	0.01	up	yes

ENSG00000182866	LCK	1.58	1.15	1.47	0.53	0.71	0.80	1.04	0.00	up	yes
ENSG00000188738	FSIP2	0.05	0.07	0.04	0.03	0.02	0.03	1.04	0.02	up	yes
ENSG00000103037	SETD6	5.84	5.94	6.15	2.88	2.84	3.00	1.04	0.00	up	yes
ENSG00000184697	CLDN6	12.00	12.80	57.36	14.25	13.12	12.63	1.04	0.03	up	yes
ENSG00000273706	LHX1	0.52	0.60	0.34	0.22	0.33	0.16	1.04	0.02	up	yes
ENSG00000197565	COL4A6	0.79	0.66	0.82	0.39	0.33	0.38	1.04	0.00	up	yes
ENSG00000164171	ITGA2	19.34	13.06	25.44	7.38	13.26	7.58	1.04	0.00	up	yes
ENSG00000153982	GDPD1	1.10	0.94	0.89	0.36	0.59	0.48	1.04	0.01	up	yes
ENSG00000167562	ZNF701	0.67	0.61	0.73	0.47	0.30	0.21	1.04	0.01	up	yes
ENSG00000143847	PPFIA4	0.46	0.53	0.29	0.21	0.15	0.27	1.03	0.01	up	yes
ENSG00000130032	PRRG3	0.16	0.17	0.19	0.11	0.06	0.08	1.03	0.03	up	yes
ENSG00000125848	FLRT3	3.25	3.30	2.82	1.53	1.52	1.54	1.03	0.00	up	yes
ENSG00000107821	KAZALD1	3.23	3.47	4.13	2.05	1.46	1.80	1.03	0.00	up	yes
ENSG00000118496	FBXO30	13.19	14.25	13.58	6.50	6.55	7.05	1.03	0.00	up	yes
ENSG00000053918	KCNQ1	4.51	4.37	4.51	2.29	2.18	2.11	1.03	0.00	up	yes
ENSG00000100918	REC8	6.46	6.15	7.28	3.23	3.64	2.91	1.02	0.00	up	yes
ENSG00000051128	HOMER3	1.80	1.83	1.79	0.81	0.96	0.90	1.02	0.00	up	yes
ENSG00000062282	DGAT2	2.34	2.30	1.83	1.19	0.87	1.13	1.02	0.00	up	yes
ENSG00000233276	GPX1	18.37	46.75	21.30	15.26	11.52	15.76	1.02	0.00	up	yes
ENSG00000185669	SNAI3	1.11	0.66	0.97	0.35	0.46	0.54	1.02	0.03	up	yes
ENSG00000118518	RNF146	3.68	7.65	7.12	4.24	3.00	1.84	1.02	0.00	up	yes
ENSG00000151376	ME3	3.82	4.59	3.78	2.22	1.93	1.86	1.02	0.00	up	yes
ENSG00000271895	AL109811	2.13	2.28	1.83	1.16	0.52	1.39	1.02	0.03	up	yes
ENSG00000260793	AC003102	0.69	1.23	0.86	0.47	0.28	0.62	1.02	0.03	up	yes
ENSG00000271383	NBPF19	1.97	1.50	2.03	1.14	1.09	0.48	1.02	0.00	up	yes

ENSG00000188167	TMPPE	1.21	1.74	1.19	0.75	0.71	0.59	1.02	0.00	up	yes
ENSG00000186493	C5orf38	0.83	1.16	0.83	0.39	0.52	0.49	1.02	0.02	up	yes
ENSG00000267288	AC138150	1.30	0.66	0.84	0.54	0.44	0.40	1.02	0.02	up	yes
ENSG00000243742	RPLP0P2	0.59	0.72	0.74	0.33	0.38	0.30	1.02	0.01	up	yes
ENSG00000035664	DAPK2	0.11	0.11	0.13	0.07	0.04	0.06	1.02	0.03	up	yes
ENSG00000198353	HOXC4	2.38	2.21	2.54	1.27	1.06	1.19	1.02	0.00	up	yes
ENSG00000263345	AC006435	0.78	0.55	0.72	0.28	0.30	0.43	1.02	0.05	up	yes
ENSG00000271601	LIX1L	0.32	0.23	0.22	0.09	0.15	0.13	1.02	0.04	up	yes
ENSG00000175105	ZNF654	2.94	3.01	2.93	1.31	1.56	1.52	1.01	0.00	up	yes
ENSG00000125898	FAM110A	6.91	6.55	5.35	3.75	1.12	4.45	1.01	0.01	up	yes
ENSG00000197093	GAL3ST4	1.37	3.30	2.88	1.30	1.55	0.89	1.01	0.01	up	yes
ENSG00000160796	NBEAL2	7.23	7.87	7.34	3.68	3.79	3.67	1.01	0.00	up	yes
ENSG00000106780	MEGF9	1.39	1.69	1.55	0.85	0.82	0.63	1.01	0.00	up	yes
ENSG00000102312	PORCN	2.15	2.40	2.79	1.26	1.17	1.21	1.01	0.00	up	yes
ENSG00000178078	STAP2	8.64	9.74	9.12	4.33	4.46	4.88	1.01	0.00	up	yes
ENSG00000143819	EPHX1	16.64	18.59	19.75	9.40	9.07	8.86	1.01	0.00	up	yes
ENSG00000273151	AC073957	1.05	1.76	1.14	0.69	0.68	0.60	1.01	0.00	up	yes
ENSG00000116711	PLA2G4A	7.85	7.57	8.22	3.57	4.16	4.04	1.01	0.00	up	yes
ENSG00000146054	TRIM7	0.73	0.65	0.65	0.34	0.34	0.32	1.01	0.00	up	yes
ENSG00000138744	NAAA	1.73	1.58	1.54	0.69	0.83	0.89	1.01	0.00	up	yes
ENSG00000104783	KCNN4	3.60	3.38	3.96	2.03	1.72	1.71	1.00	0.00	up	yes
ENSG00000168427	KLHL30	0.53	0.73	0.76	0.43	0.29	0.28	1.00	0.01	up	yes
ENSG00000144746	ARL6IP5	49.68	45.31	47.27	22.11	21.63	27.18	1.00	0.00	up	yes
ENSG00000173482	PTPRM	0.26	0.34	0.31	0.17	0.17	0.11	1.00	0.01	up	yes
ENSG00000131979	GCH1	16.90	16.30	10.92	3.81	2.86	15.36	1.00	0.04	up	yes

ENSG00000177989	ODF3B	0.54	0.63	1.01	0.44	0.46	0.19	1.00	0.04	up	yes
ENSG00000115339	GALNT3	5.28	6.63	4.91	2.96	2.81	2.64	1.00	0.00	up	yes

Supplementary Table 7. mRNAs increased or downregulated abundance (FC≥2-fold or ≤-2 fold, $p<0.05$) in ATF3-low GC tissues

Gene_ID	Gene Symbol	adjacent _2	adjacent _3	adjacent _5	adjacent _9	GC tissue_2	GC tissue_3	GC tissue_5	GC tissue_9	P value	Fold change	Regulation
9254	CACNA2D2	6.66559144	5.141969829	6.870919804	6.04009417	4.29562964	2.28986205	4.8599579	4.726044344	0.0289127	0.250360421	down
26085	KLK13	4.675624928	3.660439226	3.778277502	4.707273465	3.38119403	2.43463583	3.3727203	1.788782426	0.0254407	0.37976953	down
26658	OR7C2	5.897763338	4.386977481	5.16772547	5.972221232	4.42375757	3.88758339	4.26565846	4.265912321	0.0482689	0.41689695	down
23362	PSD3	9.7368545	9.172536582	8.988751353	9.358758245	7.96861777	7.23911087	8.50789526	8.937404572	0.0436764	0.483972124	down
NA	ASB15	5.352341603	4.81077771	4.023079441	5.019941658	3.19107458	4.01116049	3.52332995	4.150739596	0.0251914	0.46349729	down
80328	ULBP2	5.306574359	5.553446215	5.455692081	4.438995395	6.01145189	6.8122094	6.5170616	5.727464164	0.0224575	2.113237087	up
375323	LHFPL4	5.788037553	6.010437695	6.076512626	5.619211567	3.1906941	1.7194587	4.39843871	4.479453863	0.0313603	0.236167909	down
8367	HIST1H4E	10.5778232	10.99468331	10.99354037	10.63875933	11.726413	12.7427069	11.8682896	11.07309955	0.0491363	2.240377822	up
2615	LRRC32	10.52928925	9.387995214	10.71987306	10.26980933	10.5682707	11.6096235	11.7862877	12.31297107	0.0302608	2.616623049	up
7070	THY1	6.336344741	5.884195831	6.733756941	6.328662542	7.98065714	7.77631524	8.72948454	8.952125168	0.0017409	4.259855223	up
269	AMHR2	7.641818026	5.441263146	7.036776918	5.677233659	2.98307541	3.64434649	6.05011836	4.531112704	0.046704	0.257949138	down
762	CA4	8.925729704	7.902731297	8.260174831	7.542268885	4.54486022	2.67338716	7.2688201	5.475950103	0.0400404	0.187463577	down
83743	GRWD1	10.16999151	10.30045305	10.37372054	10.10711809	10.992776	12.000612	11.0448991	10.67941467	0.0427576	2.041808047	up
161582	DYX1C1	4.044459913	4.038870058	4.079178399	2.971321879	4.53304381	5.05003778	5.54914019	5.098971793	0.0109273	2.381574982	up
6752	SSTR2	5.42620541	4.7951838	5.608139808	5.049533191	1.50315286	2.75158615	3.27818409	4.084341336	0.0182836	0.237352476	down
154091	SLC2A12	6.446688333	5.276832781	5.751904253	7.249117483	2.57872148	2.2738097	4.93021947	5.250467978	0.0439017	0.239048054	down
10392	NOD1	9.234099137	9.39699668	9.899006611	9.041683105	10.5351947	10.2834007	9.78798414	11.1070431	0.024185	2.110091724	up
NA	RAET1L	1.242477055	1.792090605	2.826722998	2.321685666	3.12346404	2.69929265	3.23096619	3.666932914	0.0362886	2.081134873	up
4760	NEUROD1	5.000744092	5.658479999	5.377621109	4.73423562	4.02533084	4.06358674	4.57183947	3.299494459	0.0121933	0.442106665	down
51059	FAM135B	5.782440281	6.288089047	5.331982287	6.302436155	3.50234033	3.4671639	5.04564195	5.202902571	0.0332727	0.365510187	down

92949	ADAMTSL1	7.960037347	7.953688878	8.773762893	7.394065953	6.01113465	5.48038821	7.34063448	7.415151051	0.0496751	0.400710145	down
122618	PLD4	6.515793965	5.199526056	6.546754099	6.453380921	4.4941346	4.0603824	5.32530555	4.482772791	0.0100004	0.328637145	down
10424	PGRMC2	10.96505554	11.19844842	10.48040819	10.1239317	9.58523129	8.9546754	10.1596645	9.938856051	0.0278522	0.491719186	down
133121	ENPP6	4.691867373	5.930103506	4.043325474	3.8099431	2.66751364	2.12083227	4.21512758	2.325048873	0.0375145	0.292217685	down
51602	NOP58	6.864196717	7.112845372	6.994292093	7.469742233	8.35606748	8.85245698	7.73881241	7.513927704	0.0374153	2.119584333	up
353288	KRT26	6.944789852	5.569779068	7.104044173	6.895324709	4.89210471	4.70255497	5.48057874	4.691655734	0.0105224	0.295395501	down
127707	KLHDC7A	10.21500075	9.993553244	9.034601627	8.755079974	7.4057799	6.9169711	9.05617964	7.625929339	0.0256559	0.322415377	down
79981	FRMD1	10.34401404	7.956658062	9.479246656	9.765362376	6.96133432	4.77918892	7.45247611	7.562125821	0.0185171	0.164808016	down
85377	MICALL1	12.98776623	11.64249829	12.16258161	12.50408656	10.1438574	11.2808969	11.4654786	11.56819817	0.0324891	0.436776162	down
4929	NR4A2	8.157650539	7.383105277	6.94600727	7.115146717	6.41910119	4.94921659	5.97034171	5.866194798	0.0081478	0.3324303	down
84823	LMNB2	5.224663811	5.515633615	4.440838694	4.414756766	6.13250788	7.2052829	6.31650775	5.329512569	0.0329883	2.675702319	up
89894	TMEM116	11.4301153	10.4270786	10.84608671	11.26304761	8.79445141	9.40990083	10.1013872	10.14170282	0.0148555	0.397472356	down
253152	EPHX4	2.56898341	3.177844699	2.894683962	2.866867065	6.16742575	3.60811783	4.56133698	5.583600202	0.0307635	5.23756055	up
84549	MAK16	5.692413807	5.683440381	5.581901644	5.749244426	6.86670247	7.74875363	6.75584906	6.500453524	0.016742	2.589957793	up
200403	VWA3B	5.555362635	5.842117199	4.888620889	4.383650385	4.44710646	2.51271117	4.40053212	3.097456428	0.0424025	0.369291969	down
55748	CNDP2	5.642275068	4.689695288	5.242671429	5.822192064	3.58510277	4.15820785	3.92610121	4.223318959	0.0056947	0.374488327	down
80221	ACSF2	9.532205072	10.28919854	9.340115335	8.911077797	8.47711943	8.46221105	8.77982074	7.84004097	0.0212426	0.4413923	down
NA	ACSM6	8.892730674	7.671618905	6.790456903	6.917710171	4.8854557	4.10145878	5.10481536	6.133536709	0.0080009	0.166163698	down
151176	FAM132B	3.778087501	4.949967968	4.522779474	4.81291525	6.23331431	6.65634404	6.59886759	5.022096505	0.0157511	3.191757232	up
64220	STRA6	2.684342098	2.71386182	4.342652253	4.825964554	8.30606374	8.64832576	4.20265965	6.291117688	0.0436019	13.21291745	up
90355	C5orf30	11.46677539	10.4134361	11.00306072	12.31112853	8.64311955	7.34895007	9.9281511	10.40366294	0.039963	0.255260695	down
136	ADORA2B	2.854300392	3.314358286	2.918119974	1.749529655	5.324388	4.37265388	3.86409723	3.182171948	0.0423081	3.004321659	up
9533	POLR1C	9.880840274	10.02991898	9.845187916	9.65392641	11.0521907	11.4353674	10.6467784	10.08458016	0.041541	2.041617857	up
23082	PPRC1	8.169033311	8.133216722	8.047032411	7.491509614	9.12341301	9.4668998	8.73614893	8.556588868	0.0087399	2.041729222	up
3045	HBD	4.579892222	6.530289356	4.731533009	4.534305995	0.45861325	1.32962472	4.73280407	2.449592143	0.0457439	0.216396696	down

5083	PAX9	3.186763532	2.911032489	3.124369724	0.350832847	5.00610028	7.11212281	3.76639303	4.387941057	0.0366079	7.701103405	up
4501	MT1X	11.94862475	11.07309955	12.79034414	12.31367784	10.8702532	8.77955674	10.8046912	10.71782741	0.0348892	0.316387591	down
6274	S100A3	5.205314601	6.582231759	5.687502467	5.465001174	9.62432278	8.06463176	7.67646815	7.200210066	0.0115804	6.16674397	up
10799	RPP40	8.523184375	8.824119363	8.779938574	8.55369634	10.3654057	11.2106086	9.86215802	9.201441475	0.0368049	3.182739356	up
5799	PTPRN2	11.98445807	11.84126546	11.79654067	11.37883353	9.28941612	7.97062131	10.6507938	10.44617754	0.0365519	0.278262187	down
23160	WDR43	8.656084753	8.91185929	9.005377109	8.190383865	9.61902193	10.4091116	9.76088342	9.055341112	0.0266302	2.096702085	up
3207	HOXA11	0.284220501	1.578846354	0.281291568	0.350832847	8.02077991	3.83253933	6.22938926	5.517113735	0.0054469	58.96151193	up
80155	NAA15	6.646446494	6.564625005	6.860812132	6.385332431	7.62624574	8.45599775	7.17472412	7.448060125	0.0248451	2.198953506	up
5319	PLA2G1B	10.4793447	7.262366324	8.812335047	9.768264006	6.52078136	4.7226107	7.45446391	7.436360298	0.0362329	0.160825824	down
4014	LOR	8.593473107	6.757049907	8.20743895	8.723561292	6.54058075	6.288041	6.73499056	7.304153472	0.0497369	0.359347797	down
9221	NOLC1	9.134309161	9.226246117	9.266230785	9.421780879	10.6676219	10.8131452	10.3824683	9.632199883	0.0213363	2.255500672	up
1208	CLPS	5.37207739	5.72839803	5.596299725	5.723062038	1.4774815	3.26831632	4.62112717	3.85205516	0.0401935	0.263092815	down
6524	SLC5A2	8.176011164	6.680539757	7.997493889	8.549385441	5.97153421	5.7240078	6.78756545	7.40207546	0.0489288	0.386714668	down
51635	DHRS7	13.18781404	13.14835612	11.97860134	13.28727833	11.2099462	10.633663	12.3179733	12.1179802	0.0397688	0.416379551	down
875	CBS	7.731075874	6.714616981	8.612331348	7.981746359	4.69967364	5.45586511	6.74526922	7.05355904	0.0439404	0.322371555	down
4485	MST1	9.093198568	8.463113218	8.188355486	7.326417465	6.6134284	4.83544708	7.39511637	6.368124173	0.0271152	0.279287985	down
4494	MT1F	13.36588867	12.21098458	13.60394496	13.66332344	12.2104024	10.0614037	11.7353395	12.24862222	0.0425029	0.345840094	down
8187	ZNF239	8.700138273	8.222034722	8.907223805	8.178819195	9.49174176	10.6223999	9.57925894	9.134309161	0.0238088	2.440888382	up
55507	GPRC5D	4.118359103	4.873102193	5.014833118	4.61945703	3.40501707	2.80714188	4.29499755	2.552742955	0.0281065	0.415981234	down
2940	GSTA3	9.575258717	5.380157385	7.599030967	8.304537166	4.45259659	4.53026212	5.50589287	5.406736313	0.046901	0.101072905	down
286262	TPRN	13.51966871	13.78995242	13.2021603	13.6005484	12.7335229	10.7365814	12.6651483	12.24080367	0.0497894	0.415709045	down
10057	ABCC5	12.11727812	11.94862475	10.87932957	12.00849677	10.4986055	8.64284036	10.4273281	10.60863924	0.0276097	0.332297856	down
NA	TMPRSS7	6.499659321	6.38244765	6.243824883	6.572525691	4.87068158	4.76194848	6.02592892	4.838813074	0.0199238	0.435422842	down
56605	ERO1LB	6.653333041	4.867992929	5.993985474	6.377407027	3.5673182	5.07417711	4.95914817	4.660478089	0.0362656	0.36836758	down
54209	TREM2	6.433445525	8.131239311	6.663344619	5.699925562	7.53007396	8.87067507	8.35775135	9.377044963	0.0334122	3.164280395	up

NA	OPN1LW	13.02031426	13.92526353	14.07291828	14.5162091	12.5059825	11.7150122	13.0903278	12.11609342	0.0120918	0.346176512	down
5412	UBL3	12.13205103	11.65114741	11.6590284	11.81898359	9.7440145	9.03679222	10.9816545	11.0349676	0.0421455	0.37817735	down
124936	CYB5D2	10.73321874	10.23607225	10.45961499	10.81691974	9.57459965	8.65062784	10.0691057	9.140741628	0.02099	0.457992956	down
5827	PXMP2	13.93004402	12.59781941	13.21643406	13.47213378	11.5863129	11.6723082	12.3633134	12.25250763	0.0095923	0.386213276	down
283358	B4GALNT3	5.893707734	5.269672275	5.002254534	5.449636767	4.25842451	2.71032252	4.51004159	3.776483751	0.020124	0.357758007	down
90874	ZNF697	6.952908407	6.250842465	5.860150371	4.990809109	4.67327713	3.1863797	5.50392668	4.48273698	0.0499245	0.35303611	down
NA	AMTN	9.2380485	9.138653033	6.835846106	7.889971528	2.40451502	0.28985969	4.32077402	5.72836973	0.015387	0.052350882	down
117154	DACH2	3.333452788	3.774208324	3.153915803	3.575327671	0.25688596	0.259434	1.51811618	0.281671399	0.0009767	0.145160108	down
1442	CSH1	7.567694124	6.642863003	7.547560588	8.057908894	6.18707103	6.17218903	6.46539874	6.8002308	0.0293224	0.463911005	down
7076	TIMP1	12.3101591	13.34590135	13.42175877	12.36633017	14.153067	13.565939	14.4671936	14.95380024	0.0146431	2.669183732	up
2191	FAP	6.525430153	6.13535425	7.475251	6.976100811	9.94371858	9.33132552	9.22295054	10.81719124	0.0007498	8.645171406	up
3209	HOXA13	0.474500032	5.713700248	0.426839839	0.350832847	9.42641484	5.36138443	7.42952558	6.2908017	0.0182525	17.34601745	up
79747	ADGB	2.712099666	3.221678078	3.615083748	3.725552765	1.69498563	1.6671681	2.8752955	1.809705475	0.0133226	0.417206764	down
174	AFP	4.247309775	3.047014143	2.921037349	3.944828467	0.6009826	0.36678129	3.45014489	0.381563715	0.0449431	0.299388031	down
6328	SCN3A	4.903388293	6.303035445	5.981922703	6.08677371	3.44090612	3.33679577	5.28099514	5.011507268	0.0490652	0.383661074	down
6822	SULT2A1	12.34711667	10.4122839	10.71673642	7.740452361	6.50669417	0.45194642	8.10680134	5.624627366	0.0454162	0.049270817	down
4329	ALDH6A1	11.47120398	10.39834007	11.17559243	10.97018428	9.06074885	8.48838019	10.117707	9.922705291	0.0156458	0.349650813	down
54820	NDE1	5.130121546	6.068117012	5.864649219	5.81427281	7.06666634	8.86266695	6.7989582	6.530440282	0.0495244	3.708025644	up
3206	HOXA10	3.847648839	7.406757738	3.993216938	3.36171831	10.8799403	10.7147238	10.1046446	8.024635708	0.0046192	23.42689225	up
1139	CHRNA7	8.721991794	9.468628854	7.206758347	8.476463104	7.79577953	5.32641609	7.0694002	6.700306887	0.0472785	0.306259773	down
51617	HMP19	5.46893112	4.514042862	5.958012336	4.795355522	2.59824516	1.47705913	3.96697627	2.566826355	0.0082569	0.193556586	down
134526	ACOT12	6.52438539	6.670647115	6.842545848	7.241206792	5.01951501	4.98727607	5.45170777	6.052595573	0.0043314	0.378916195	down
336	APOA2	4.847583074	5.422269757	5.462641444	6.215665736	3.81690268	4.32517015	5.26125568	4.47922474	0.0480733	0.498682006	down
22852	ANKRD26	14.28610107	14.89429869	15.34620262	15.66700618	13.9639827	13.0647383	14.5564811	13.38461915	0.0263745	0.411746236	down
256076	COL6A5	6.877981935	3.159485208	7.303373677	3.739219959	1.18130455	0.29517552	4.46692145	0.585853931	0.0445249	0.091015637	down

347741	OTOP3	8.237607992	9.518880679	7.960138301	8.168037619	7.25375863	8.16152224	6.79270811	7.074009339	0.0482154	0.435437418	down
148066	ZNRF4	2.279311379	1.666379624	2.287522975	2.763925828	4.12835754	3.42489307	3.46632431	3.089288901	0.0064804	2.42559316	up
26586	CKAP2	6.522383228	7.347962238	6.298161639	6.626020322	8.47793202	8.57106848	7.60827044	7.093725799	0.0315048	2.471836592	up
10381	TUBB3	7.755485496	7.053771574	6.844019525	6.914298284	9.26546523	9.22876868	8.46061654	9.004438505	0.0006115	3.561089663	up
54757	FAM20A	8.986050608	7.330931999	7.833058714	7.867208611	4.97006792	6.12435582	7.22852784	6.160668307	0.0192137	0.285602244	down
54498	SMOX	7.885643148	7.541530617	7.602679382	7.751233562	6.07288574	6.17929484	6.55181366	6.679497764	0.0007253	0.403671294	down
133	ADM	10.98856105	10.97277108	10.53688879	10.48173018	7.95903076	8.25360914	10.1818895	9.780621229	0.0498409	0.373023966	down
9026	HIP1R	8.685469174	8.0478046	8.697219237	8.347560748	7.32458595	6.73264533	8.04969665	6.708398671	0.0210522	0.448925708	down
375775	PNPLA7	12.67405392	11.30229941	12.43247806	12.83173652	10.9161873	10.4536696	10.2731732	11.73118573	0.0215296	0.364370409	down
1748	DLX4	3.187211133	3.741640773	4.442831061	4.383686928	4.42358758	5.7161973	4.93701317	4.776924156	0.0442194	2.02862101	up
51373	MRPS17	8.225894572	8.16554205	8.283957587	8.490677572	9.10288619	9.86864398	9.41065648	8.91236982	0.0114985	2.104401051	up
162466	PHOSPHO1	12.00776656	10.59404299	12.01226015	12.28965222	10.786443	10.1072118	10.6741088	10.64824668	0.0477664	0.412660452	down
NA	TRIM73	8.978690298	6.186911378	8.487378267	8.523541227	5.48120694	5.47545986	6.92851001	6.138876325	0.0392975	0.21579054	down
79924	ADM2	5.055588786	5.159279256	5.128412913	5.901551895	3.60372841	4.51867762	4.45182268	4.285919647	0.0089978	0.466997239	down
93109	TMEM44	7.460938386	8.119742765	7.941093176	7.495197672	9.39653853	9.06904896	9.04614857	8.351083665	0.0054106	2.3466351	up
57214	CEMIP	1.791383282	3.703135939	4.044252794	0.350832847	7.9845257	7.6887258	7.74060878	4.054486564	0.0139008	20.14262004	up
6528	SLC5A5	10.26313524	11.74564744	9.711113555	8.38632193	6.08508965	4.98528666	8.50995582	5.422737587	0.0116956	0.086881149	down
63935	PCIF1	13.82145816	13.18593021	14.11645616	13.76826442	12.9388058	12.2242498	12.9640412	12.79605462	0.0090913	0.499714072	down
3014	H2AFX	9.466758482	10.62169522	10.02250011	9.431733316	11.4635114	11.4263062	11.7634293	9.966898931	0.0460793	2.50463177	up
9688	NUP93	11.12838226	11.32876243	11.24286721	11.38360366	11.9082071	13.0402356	11.8842047	12.10118129	0.0361432	2.063470958	up
124930	ANKRD13B	7.92954973	7.371964019	7.986579813	7.28544395	9.15969335	8.97158982	7.7965297	9.283184105	0.0337826	2.343254073	up
85019	TMEM241	5.404606801	5.510417998	6.027667518	5.376633684	6.25491558	6.75574061	6.84425585	7.142293625	0.0030123	2.262666989	up
84239	ATP13A4	9.714882117	9.270609998	9.084034093	9.133291484	6.47920343	5.05019056	8.78045668	6.404145128	0.0404694	0.252417891	down
3425	IDUA	8.569692442	8.329593988	9.004871288	8.095748027	7.84073788	6.67406171	8.11161236	7.020768921	0.0402691	0.495518148	down
53905	DUOX1	7.802228282	7.601004583	7.461033985	7.614219447	5.61650213	5.26644456	7.03864592	6.492890482	0.0312244	0.391512316	down

151230	KLHL23	6.615874387	7.052311929	6.620495249	6.442353508	8.11245143	8.35449734	7.7114241	9.193556931	0.0080274	3.362044265	up
153396	TMEM161B	9.47721675	8.856266543	9.391922479	9.848154458	7.38516752	8.01742005	8.69200574	8.666588404	0.0218455	0.449943305	down
768239	PSAPL1	10.02430361	11.44628958	7.78061801	5.775598627	2.58647464	0.26473293	7.84556618	3.167099925	0.0417243	0.059953859	down
341	APOC1	8.071740215	9.230139684	9.61532594	8.119052449	9.91503414	12.9448616	11.330534	10.24703404	0.0322859	6.543007859	up
79734	KCTD17	8.909108349	9.024356632	9.517195588	8.871312102	9.76481946	10.7831043	9.71723974	10.33063965	0.0157287	2.161882959	up
1718	DHCR24	9.568084349	8.550092279	8.870675074	8.906012915	8.009442	6.17283283	8.42964447	7.157538061	0.0469321	0.392659609	down
11078	TRIOBP	14.29565272	13.45026785	14.59652858	15.08086628	11.7972499	11.5040924	13.3969741	12.93346118	0.01545	0.276335493	down
9159	PCSK7	11.07413393	12.81536916	11.25842514	12.24862222	10.3806872	9.97411241	10.415088	11.23798138	0.0394704	0.366886245	down
80310	PDGFD	11.16610823	10.06654082	10.87650496	11.50517403	6.76361683	6.89900935	10.0075403	8.768047246	0.0301383	0.207593117	down
54976	C20orf27	12.17622213	11.26304761	11.67518637	10.4083043	12.0698197	13.1674135	12.1058212	13.63558788	0.0453525	2.614906964	up
653	BMP5	5.159912549	6.428670162	4.801687603	3.949320731	1.55806965	1.25166063	4.51444667	2.606312078	0.0312849	0.20740682	down
9258	MFHAS1	8.598913765	8.838939204	8.612163652	9.00186324	10.08271	10.6442254	9.53919641	9.353793825	0.023911	2.33373464	up
285848	PNPLA1	6.21601926	4.532981029	5.620946893	6.057041505	3.9529936	2.08579009	4.37020736	1.85091821	0.0197423	0.206394113	down
319101	KRT73	12.76594176	12.07012875	13.32721568	13.90049615	10.5579926	10.211802	11.7069781	12.33670369	0.0299966	0.304312437	down
4016	LOXL1	10.53860017	9.804920777	10.66454095	9.67540099	11.0904454	10.8357788	10.9930927	12.40829733	0.0435502	2.380875163	up
63979	FIGNL1	6.812781442	7.612982913	7.147300911	6.616744412	8.26705234	9.15491837	8.78114993	7.190908396	0.0472936	2.67137623	up
2853	GPR31	4.217574285	3.777602138	4.859392342	4.859901175	2.78264186	3.62418133	3.24209141	3.385481669	0.0132249	0.432854165	down
83732	RIOK1	8.400609172	8.41582103	8.387685804	8.303449255	9.54026053	10.0671111	9.1957627	8.913136474	0.0234334	2.169289993	up
54855	FAM46C	12.57944376	12.81420106	13.52817064	14.01760607	10.3329875	10.4958811	12.6679703	12.05906861	0.0411099	0.323788391	down
79828	METTL8	4.919114733	5.407367028	5.808898906	5.23542618	6.23164461	7.38404149	6.11665659	6.986264269	0.0133971	2.633954393	up
9374	PPT2	7.630250377	7.080763735	7.680351875	8.295368494	6.98772777	6.47404806	7.04763018	6.215987019	0.02239	0.495373378	down
254065	BRWD3	11.06250087	11.66058031	12.39404442	12.306467	11.0155456	9.81275569	11.3753859	10.45217651	0.0420459	0.444084339	down
7188	TRAF5	8.845454963	8.107651127	9.189939457	8.996807466	10.0707792	10.6608253	8.91066604	10.2109028	0.0437029	2.382029776	up
53345	TM6SF2	7.874769537	8.931052671	8.150799801	7.698422176	7.83390461	6.69260839	7.33481975	6.820316794	0.0389298	0.498909152	down
NA	FAM53A	8.917022292	6.78701787	8.446071693	9.213579854	6.10060153	6.37783106	6.92528656	6.713825546	0.0381585	0.246241344	down

9717	SEC14L5	6.825484139	5.756664095	6.486675281	6.743967759	5.78414997	5.04789981	5.11324516	5.444434091	0.0119532	0.456557189	down
3930	LBR	11.44232773	11.85811197	11.50798029	11.1763707	12.52004	13.2227567	11.9659351	12.76352769	0.0151051	2.251124991	up
960	CD44	1.430542606	3.945597052	2.799641839	4.382543363	6.20479291	6.76025936	5.58088259	4.580059912	0.0199171	5.529698263	up
3354	HTR1E	8.837789932	5.116805186	8.436912319	8.871157868	2.97481594	3.10935724	5.99444546	6.283854306	0.0445749	0.121006306	down
81539	SLC38A1	10.74874246	10.64398107	11.34459496	9.995266536	11.5505468	12.7401494	10.9915265	12.30018548	0.0481418	2.437454288	up
27033	ZBTB32	9.162531783	9.989090459	10.43773578	10.89887258	7.99683669	7.17091476	9.50762098	8.055168263	0.0210215	0.285206579	down
10940	POP1	8.29125423	8.681555208	8.410632816	8.227055422	9.65419167	10.1960617	9.37087778	8.803650688	0.0259603	2.26410439	up
636	BICD1	1.4259037	2.110549707	3.179813491	2.242827162	2.6534275	4.07474122	3.88858241	4.10798826	0.027995	2.65362545	up
55226	NAT10	6.217775866	6.20388213	6.235868408	6.843233512	7.54257796	7.81179179	7.66102628	6.776709635	0.0107871	2.137471163	up
347732	CATSPER3	7.743952566	6.298608213	8.618775262	9.826807847	5.39079621	4.72887592	6.29888823	6.998286459	0.0499934	0.172517273	down
50632	CALY	10.06161112	8.796729755	9.837568667	10.31706853	8.68835689	7.95149447	8.61546697	9.226246117	0.0389043	0.445783384	down
3801	KIFC3	11.26472693	11.53514668	11.19211771	11.39442404	10.0246722	8.84245783	10.4293856	10.16701553	0.02181	0.385220643	down
9963	SLC23A1	3.551481519	4.974253217	3.245751922	4.82477568	0.407652	2.10267451	3.18082011	3.020674983	0.0483236	0.281600287	down
357	SHROOM2	8.639771644	7.456107955	8.352501439	8.323176838	6.45041721	6.78278806	7.06710994	7.33574942	0.0081256	0.403731085	down
23344	ESYT1	4.64069597	4.944922733	5.036516576	5.305338976	6.07640659	5.98998295	6.35549545	6.339120055	0.0006023	2.293974246	up
204801	NLRP11	4.755511199	5.330600257	5.8227052	6.070903894	3.85657829	4.23947022	4.16405242	4.560866628	0.0136651	0.39201301	down
151050	KANSL1L	10.2183854	10.05051057	8.46209011	8.48152383	6.10149644	6.14141258	7.77204655	6.839994896	0.0063938	0.159492084	down
50808	AK3	10.00264873	11.04489909	11.87464041	11.81932296	9.55580523	8.92958032	10.6405764	8.963600918	0.0311322	0.317470905	down
158405	KIAA1958	9.256244814	7.389050488	9.097061887	9.295428478	6.79679146	7.0219125	7.9557025	6.565773504	0.0272359	0.298770893	down
NA	ILDR2	2.673469673	2.965874975	3.592378128	1.340090859	4.13521748	9.02310721	6.37112316	4.725748245	0.044339	22.47867472	up
6550	SLC9A3	8.134504165	6.631978666	8.126690665	8.359943169	5.55512098	5.0429443	6.91994324	6.2258915	0.0164846	0.279207196	down
54443	ANLN	1.952394936	5.147740073	3.844657689	3.191501379	5.48987641	6.26418594	6.57794977	4.582359546	0.0388579	3.840797612	up
6691	SPINK2	7.190980475	7.86681226	8.299284273	7.822365525	6.74507963	6.32282981	7.17998396	6.573672557	0.0105226	0.464303441	down
2243	FGA	6.626152942	4.099467114	6.208794352	6.369708788	0.96770703	0.25560753	4.55021031	3.790206472	0.0380078	0.148259424	down
3920	LAMP2	9.945070202	9.320512009	9.57692153	10.36525986	7.71105759	8.0471445	8.63358601	9.135973692	0.0126307	0.386691938	down

56624	ASAH2	7.739301657	9.279145518	6.755492005	8.010297942	6.24480971	4.19163164	6.74003376	6.531100712	0.0426232	0.244395755	down
389840	MAP3K15	4.443849207	3.002219614	4.451822685	3.351362304	2.42535732	2.01082732	2.05855449	2.507246851	0.0196619	0.311199463	down
84632	AFAP1L2	6.836766111	6.989031985	7.092317356	6.220523702	7.66830593	7.80234446	7.54478993	9.109921293	0.0326437	2.577348167	up
114132	SIGLEC11	9.403315901	7.550913936	9.165844908	10.17306484	6.90828702	6.54779181	8.28777095	7.495197672	0.0435086	0.272447228	down
374286	CDRT1	4.506561481	4.996061821	4.283444282	3.907576915	6.12974942	6.40532764	6.04820983	4.525605685	0.041153	2.737656628	up
113622	ADPRHL1	8.792683715	7.847341628	8.479829262	8.145137171	7.52891781	6.28563846	7.76759414	6.954292904	0.0286289	0.460180126	down
283554	GPR137C	11.23723394	10.0694346	11.19540558	11.58996989	10.1288974	9.45275394	10.1603585	10.35343534	0.049199	0.479247544	down
1056	CEL	10.86013692	9.865650542	11.28183928	11.36941495	9.77270172	8.83687746	9.82027499	10.27755157	0.044331	0.438650547	down
2892	GRIA3	4.198611427	5.023748089	4.905149896	5.567991316	3.34780127	2.87454671	3.88020834	4.090445075	0.0127805	0.384075438	down
2805	GOT1	12.30018548	11.52493386	11.78132488	12.16258161	10.9978191	10.1515782	11.1464207	10.62558899	0.0058748	0.43672572	down
51660	MPC1	13.40447545	12.66418895	12.66418895	12.83107619	12.1136237	10.8536896	12.1289022	12.08615157	0.0309292	0.485639927	down
84708	LNX1	6.91564605	6.494605536	6.352935281	6.315740969	5.11926847	4.11257994	6.06243439	5.085536353	0.0311767	0.410535526	down
3624	INHBA	3.630752692	2.937714836	4.080688465	3.755483826	6.6880069	4.75556521	6.40540568	7.559978327	0.0123527	7.995845702	up
57139	RGL3	7.453138604	7.711126614	6.66010911	6.741832468	5.76318448	5.33530902	5.84660087	6.429781187	0.0096399	0.402128599	down
80333	KCNIP4	6.808988573	4.905031748	6.483595346	6.20176286	4.4947384	4.18561247	4.82780988	5.64220426	0.0486498	0.390208996	down
NA	POTEJ	10.28827009	10.10244578	9.209336812	8.676959384	11.3031895	11.6216217	9.8183954	11.23723394	0.0417389	2.68419537	up
3000	GUCY2D	5.811493701	4.736715671	5.920471482	5.065831205	4.94961909	3.86821622	4.69285468	3.582793432	0.0437694	0.471215458	down
79444	BIRC7	3.382892993	3.399572599	4.338476652	4.725385755	4.72726272	6.66660779	5.83671318	5.160151475	0.0248424	3.265695412	up
5802	PTPRS	7.455597341	6.238023402	7.399099406	7.287175366	6.29704492	5.47091884	6.39052464	6.103846259	0.0304102	0.478572555	down
90527	DUOXA1	8.280792573	6.618316358	7.035106943	7.125201494	5.7218493	4.83264505	6.08205327	5.950262721	0.0129478	0.310069065	down
2620	GAS2	6.242521695	4.813488647	6.416720357	6.435775339	4.27946303	4.16867172	5.39359905	4.562633807	0.0317523	0.373673852	down
7448	VTN	4.356938465	6.000207992	6.024531398	4.368840622	2.20127381	1.89163533	3.72764155	3.876916426	0.0178635	0.212901481	down
84624	FNDC1	5.720354436	4.15872207	4.898844048	4.029779288	7.02210693	5.18327882	6.85343287	7.786466249	0.0276766	4.30599227	up
169436	STKLD1	3.899084371	3.202398365	3.566626709	3.605417423	1.16953494	0.29382232	2.550602	2.718947409	0.0433692	0.330634609	down
51475	CABP2	10.41410964	10.25957699	9.262748874	9.560436327	8.62473095	7.77305058	8.62767208	8.319573229	0.0050759	0.335505909	down

11069	RAPGEF4	7.294268532	6.605932998	7.178524184	6.886288855	4.05250491	4.19074769	6.30040363	5.856458895	0.0412441	0.331594145	down
140883	ZNF280B	3.411634262	3.423353901	4.331636075	3.449068348	5.05202559	5.74774991	3.94286622	5.009160765	0.0323961	2.565343131	up
25807	RHBDD3	11.35575776	10.5116924	10.82239128	11.18998466	10.0288212	9.39732905	10.0079961	10.31064781	0.0086452	0.488437518	down
2057	EPOR	9.054985425	7.508004787	8.776662455	8.059264144	7.13512685	7.46051313	7.75949418	6.471683424	0.044852	0.438368059	down
254187	TSGA10IP	8.148179878	6.321336446	8.140156825	8.506346264	5.83747379	5.76989704	6.5649174	5.46403838	0.0239447	0.248225937	down
79957	PAQR6	6.936453086	7.387857396	8.141271393	8.462327728	5.90133223	5.51483033	6.94742589	5.365147514	0.0112178	0.292026827	down
1114	CHGB	7.770938862	12.13337749	8.880501642	7.518442981	5.20640269	2.78327374	7.30226272	5.534346542	0.0342229	0.046215863	down
163590	TOR1AIP2	8.16123123	8.310243938	7.636137538	8.157966517	6.69564724	5.5337645	7.19451716	7.523932677	0.0492649	0.441541143	down
80852	GRIP2	10.34205973	8.057659161	9.250400013	9.800635193	6.0705048	3.21276032	6.97357245	7.524479128	0.0289285	0.126013664	down
29106	SCG3	5.092089595	6.169044639	5.738757807	5.220600096	3.00779296	0.27827415	4.60326566	0.314533108	0.0424696	0.176903526	down
4982	TNFRSF11B	2.550102007	4.67489568	3.733032614	3.034658014	4.89285673	5.97275686	7.36498831	4.750547753	0.0276299	5.374702724	up
NA	LCN9	4.948105946	5.227133167	4.813668464	6.198520158	4.03741781	4.76086243	4.36856033	4.062265616	0.0422641	0.476162603	down
284611	FAM102B	8.069512334	9.166197078	8.209762586	7.409525228	9.75429619	10.0982056	9.18844463	9.272785038	0.0237672	2.414997602	up
6947	TCN1	14.30161301	11.36783147	11.47762086	12.90024047	9.97209484	8.4014004	11.0446595	10.4498935	0.0305017	0.145610787	down
126433	FBXO27	8.685791787	7.645306091	8.629222773	8.151299531	6.44191813	6.49953924	7.45457721	7.174439752	0.0073568	0.384811174	down
8909	ENDOU	3.523528476	2.922878471	3.182435811	2.388924167	4.00417585	4.3641703	4.78902455	4.297915243	0.0046325	2.51373052	up
NA	SLC26A5	5.757834301	6.071546616	4.149405099	5.482793116	3.59312864	1.70627979	4.29320318	1.944057856	0.020659	0.210984186	down
28966	SNX24	8.055168263	7.999928638	7.383105277	7.509525331	6.81313419	6.22003448	7.18183219	6.670777466	0.0085481	0.498293625	down
84706	GPT2	12.70457257	10.99291949	13.72073656	13.01857774	9.36780865	9.64047856	11.5273263	11.65746539	0.0492849	0.250421766	down
84915	FAM222A	9.170415366	7.844898268	8.37229418	8.995457878	6.96737711	5.23111872	7.45216998	7.829852175	0.0494764	0.343071842	down
3625	INHBB	3.747415947	4.500837897	3.09724717	2.862706878	5.42866292	4.48472705	4.87864191	4.531621708	0.0312796	2.273646129	up
1773	DNASE1	10.07811065	11.55467776	10.13860918	8.951896128	7.30660858	6.8501505	9.28154789	7.123570268	0.0164245	0.181290489	down
222008	VSTM2A	7.392026063	6.822405633	5.959809241	4.022513629	2.99524225	4.63519528	4.42777871	1.284360957	0.0443207	0.157889277	down
57819	LSM2	11.99910839	12.27694558	12.25408887	11.9608571	12.8214952	13.9274128	12.9358106	12.63429155	0.0406049	2.064533747	up
NA	DGKK	5.464585937	6.359281937	5.274618097	5.718464618	4.35211107	4.63800547	5.24677385	4.454954209	0.0164928	0.483519436	down

79017	GGCT	10.64502959	11.00546657	10.38905486	10.5262934	11.9706394	11.9470055	11.7843711	10.90688994	0.0192313	2.071599146	up
152189	CMTM8	9.655309584	9.111649425	9.477653197	9.351048926	8.13208151	7.73775452	9.03290774	8.182462576	0.0186733	0.480016945	down
7021	TFAP2B	6.893495455	3.505184061	7.140235843	8.067863878	0.24889076	0.2813395	4.16221517	3.445560905	0.022778	0.057820584	down
NA	INSC	4.833741586	5.474962072	4.921260946	4.608112412	3.59642956	2.93014741	4.165054	4.579618627	0.0412402	0.482201431	down
221830	TWISTNB	8.298032946	8.33028035	8.405111726	7.812519243	8.62630949	9.99600643	9.15893448	8.976973139	0.0352018	2.075858	up
29899	GPSM2	8.470182015	9.90968915	8.622607187	8.003915178	10.2936406	9.98223443	10.2161313	10.02467215	0.0408704	2.286214239	up
59285	CACNG6	5.476161151	5.138803873	6.303374819	6.291737465	4.6630207	4.14437069	5.05699633	4.81187763	0.0222314	0.44036569	down
92086	GGTLC1	7.219433434	8.110352215	6.757224361	7.622395151	6.61302512	6.6262545	5.37755019	6.280340542	0.0264583	0.432483473	down
270	AMPD1	7.741450469	10.27373879	8.91281372	8.257989724	5.8513107	3.59431115	8.13587868	5.374564133	0.0383981	0.175254842	down
672	BRCA1	6.473173802	6.953981984	6.552259898	6.097524523	7.85624857	10.0405207	7.97779522	7.40883709	0.0492047	4.549976211	up
8607	RUVBL1	10.37326791	10.68132265	10.61450914	9.841461351	11.677806	13.266157	11.4510692	11.03255082	0.0492677	3.304621014	up
6580	SLC22A1	6.824823038	7.587963677	8.237729333	7.759548686	6.11426274	5.66324039	6.78827324	5.935383928	0.0086292	0.353838156	down
388564	TMEM238	15.32099837	16.30073323	16.49153313	16.61702497	14.8841311	14.2384485	15.9989756	14.34947985	0.0421181	0.432475597	down
2081	ERN1	5.471567719	5.8501854	6.307781277	5.228456913	4.58102798	4.71650774	5.1109461	4.207575763	0.0134622	0.472003253	down
10871	CD300C	7.922398201	5.964196013	8.019734852	8.176657312	4.58039448	5.89014919	5.78736135	6.754642657	0.0430265	0.288609257	down
128864	C20orf144	4.090907404	5.506804905	3.882709681	5.336422205	2.17887766	2.51901124	3.42568117	4.135712056	0.0361447	0.327914342	down
4036	LRP2	3.478570382	3.432876216	3.953017123	4.176079178	1.08976711	0.48235732	2.68696027	3.093410148	0.0494584	0.333271466	down
2172	FABP6	5.274192787	6.7066226	4.521373909	4.885049425	7.90763099	9.99781423	7.60939522	6.110058021	0.0414528	7.804006079	up
9509	ADAMTS2	7.604274763	7.581538898	8.155274764	7.437763202	8.24849558	8.71211008	9.76226168	10.62502945	0.049095	3.754660515	up
9933	KIAA0020	9.302292058	9.548610954	9.481113206	9.595799789	10.4253977	11.2675575	10.3854989	9.908908074	0.0336786	2.140174687	up
348013	TMEM255B	3.990685918	4.182198372	4.192263418	3.85205516	4.64427696	5.25694648	5.04524102	5.320348336	0.0027442	2.040086575	up
285175	UNC80	4.405246916	3.570729814	5.428742761	3.499185476	1.49608923	1.33853217	2.77383462	1.315363274	0.0054087	0.167835043	down
5570	PKIB	8.060472013	7.631020134	6.174876385	5.660432701	1.61801142	0.2741197	6.11683912	3.825975083	0.0471189	0.149417697	down
57130	ATP13A1	9.417476342	7.508847542	9.711528074	9.888720802	7.11495605	7.63625529	7.97010287	6.909862205	0.0438634	0.267144277	down
7004	TEAD4	8.507694461	9.561838399	8.725054677	8.557090582	10.3563658	10.0184931	10.1112092	9.683965685	0.0089587	2.227963933	up

148281	SYT6	6.278256412	4.814726712	6.517013177	6.211122952	4.49999437	4.90666317	4.54013486	5.23733326	0.049639	0.418566605	down
201294	UNC13D	7.787437444	7.203892184	8.253058159	8.764834155	6.60072448	6.48748896	6.87780737	7.416967749	0.0316708	0.428788989	down
15	AANAT	6.028777237	5.795538327	6.514861827	6.609256413	4.98111303	4.86245846	5.06836642	5.758292549	0.00878	0.478702184	down
7058	THBS2	8.100049918	6.838005507	9.404241573	6.658008152	10.5241488	8.45945985	10.1317001	11.45657914	0.0366972	4.931609621	up
51200	CPA4	5.118958977	2.660730285	4.573287113	3.987140631	2.5291152	0.24500408	2.62499791	1.734561686	0.0235057	0.203792269	down
4807	NHLH1	1.961929753	3.548707367	2.565133313	2.508663515	4.26490109	5.27220925	3.60930334	3.333988983	0.0372252	2.946220996	up
5191	PEX7	10.64422538	9.74119576	10.31683056	10.57408684	9.2689515	8.12505637	9.95901258	9.324025184	0.0491857	0.481784357	down
54769	DIRAS2	4.022086016	3.353885684	3.91879432	3.577431562	0.23117632	0.26577113	3.32877749	0.288430326	0.0366186	0.254890801	down
NA	LRRC26	10.73809532	10.10138717	10.80943947	11.21977506	9.25025456	8.52350589	9.56107047	9.391434844	0.0032369	0.34410121	down
285141	ERICH2	7.466251165	5.40880988	7.253122292	7.775599911	4.71375059	3.18082011	5.78617685	5.808427238	0.0428919	0.247948657	down
79365	BHLHE41	5.931744212	5.718156134	6.308726208	6.620372849	7.70189399	7.90645013	6.66130001	7.78182417	0.0098493	2.640966094	up
283248	RCOR2	6.361716959	6.403370863	5.836316487	5.565966789	7.84922454	7.50429921	7.16211472	6.778041127	0.0060627	2.450803504	up
3483	IGFALS	10.1033845	8.7833466	9.813537455	9.79304622	8.39384132	8.34983116	9.11887679	8.644873472	0.0323695	0.486482825	down
1775	DNASE1L2	6.899570048	8.070997769	6.747752777	5.827424452	5.14860775	5.96969146	5.76653838	4.320837907	0.0380869	0.312300812	down
8927	BSN	4.865237628	5.119200475	5.585033845	4.403368455	4.11207904	3.29399408	4.11534543	4.328508525	0.0222456	0.484900819	down
7036	TFR2	11.68768367	9.090775774	10.89846148	11.27240271	7.31730418	4.08256433	9.49899387	8.128208324	0.0483584	0.143415986	down
5149	PDE6H	8.41724367	8.862298416	9.598920485	9.954885044	7.55223805	7.0211476	8.46638904	7.538331448	0.0149883	0.332860944	down
84667	HES7	12.47114598	11.82140987	12.64170223	13.08198566	11.224252	11.2257725	11.5636098	11.81379321	0.0191932	0.468594658	down
64137	ABCG4	8.614994399	7.965147122	8.789763928	9.210451544	7.74251475	7.13584047	7.92345838	7.839139291	0.0237654	0.492892677	down

Supplementary Figures

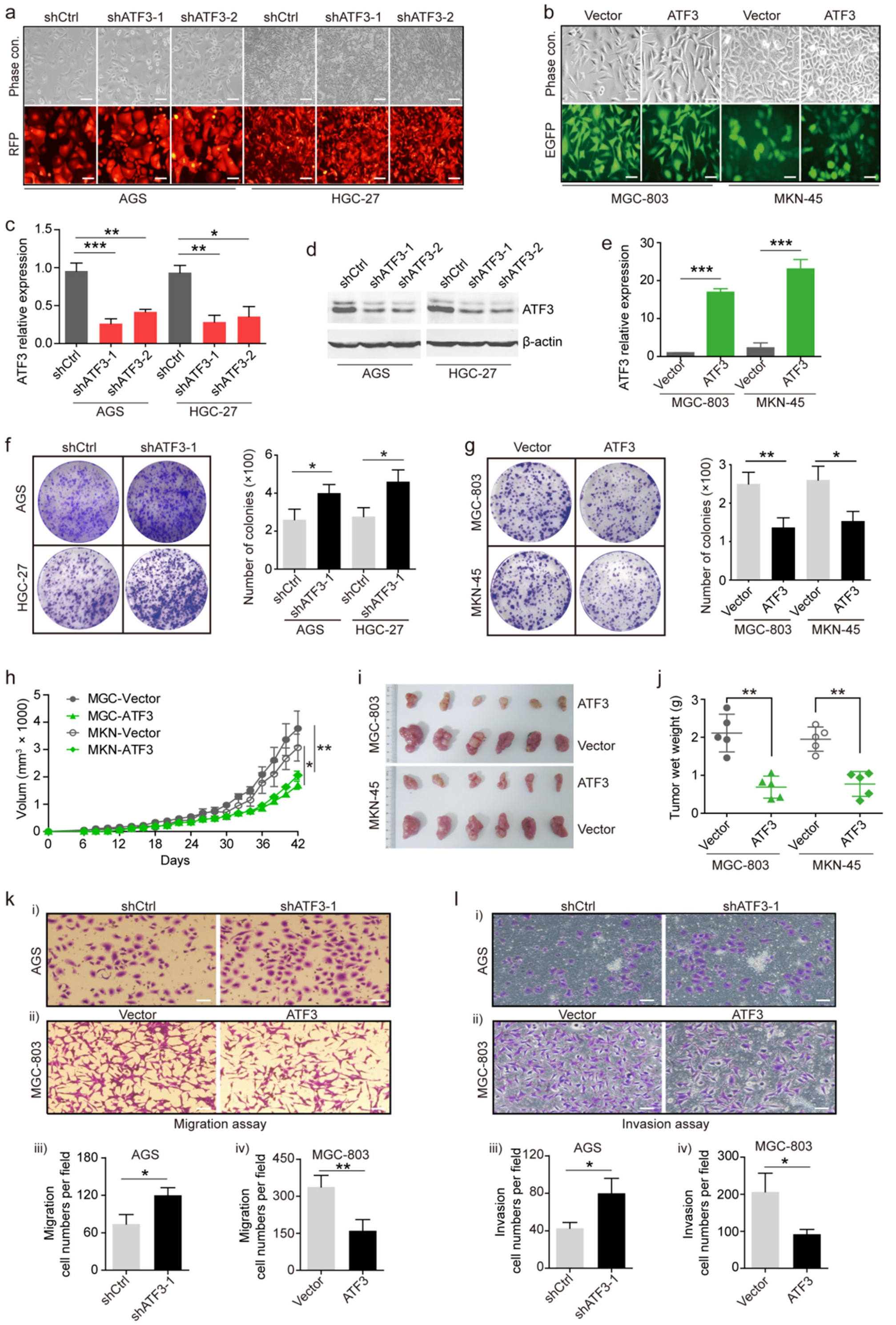

Supplementary Fig. 1 The effect of knockdown or overexpression of ATF3 on the proliferation of GC cells. a AGS and HGC-27 cells were infected with shATF3-RFP (Red Fluorecent Protein) lentivirus (shATF3-1 and shATF3-2) or scramble shRNA lentivirus (shCtrl), respectively. The infected cells were screened for 1 week by

puromycin and more than 90% of the lentivirus-infected cells expressed high RFP protein as compared with mock control cells (scale bar, 100um). **b** MGC-803 and MKN-45 were infected with ATF3-EGFP (Enhanced Green Fluorecence Protein) lentivirus or EGFP-control lentivirus, respectively. Compared with mock control cells, more than 90% of the lentiviral-infected cells expressed high GFP protein after 1 week of screening with puromycin (scale bar, 100um). **c** and **d** The mRNA and protein levels of ATF3 were detected by qRT-PCR and western blot in ATF3 knockdown AGS and HGC-27 cells. Lentivirus containing scramble shRNA was used as negative control. **e** ATF3 mRNA levels were detected by qRT-PCR in ATF3 stable overexpressed MGC-803 and MKN-45 cells. **f, g and h** Tumor growth curve **(f)** of ATF3 stable overexpressing MGC-803 and MKN-45 cells (or negative control cells) in the xenograft model was presented, followed by the collection of tumor nodules **(g)** and tumor weight records **(h)** respectively. **i** and **j** The proliferation capacities of ATF3 knockdown **(i)** or overexpression GC cell lines **(j)** were detected by colony formation assay, with bar charts showing colony numbers. Results were expressed as mean ± s.d. of 3 independent experiments. **k** and **l** Representative images of transwell migration assays (k) and invasion assays (l) of ATF3 knockdown AGS cells (k-i, l-i) and ATF3 overexpressed MGC-803 cells (k-ii, l-ii), the control lentivirus infected cells were used as the corresponding control. Relevant quantification of the results is shown in the bar graphs (k-iii, iv; l-iii, iv; lower panels). Results were expressed as mean ± s.d. of 3 independent experiments, Scale bar: 50 µm. *$p < 0.05$, **$p < 0.01$ and ***$p < 0.001$ by student's t-test compared with paired normal or control group.

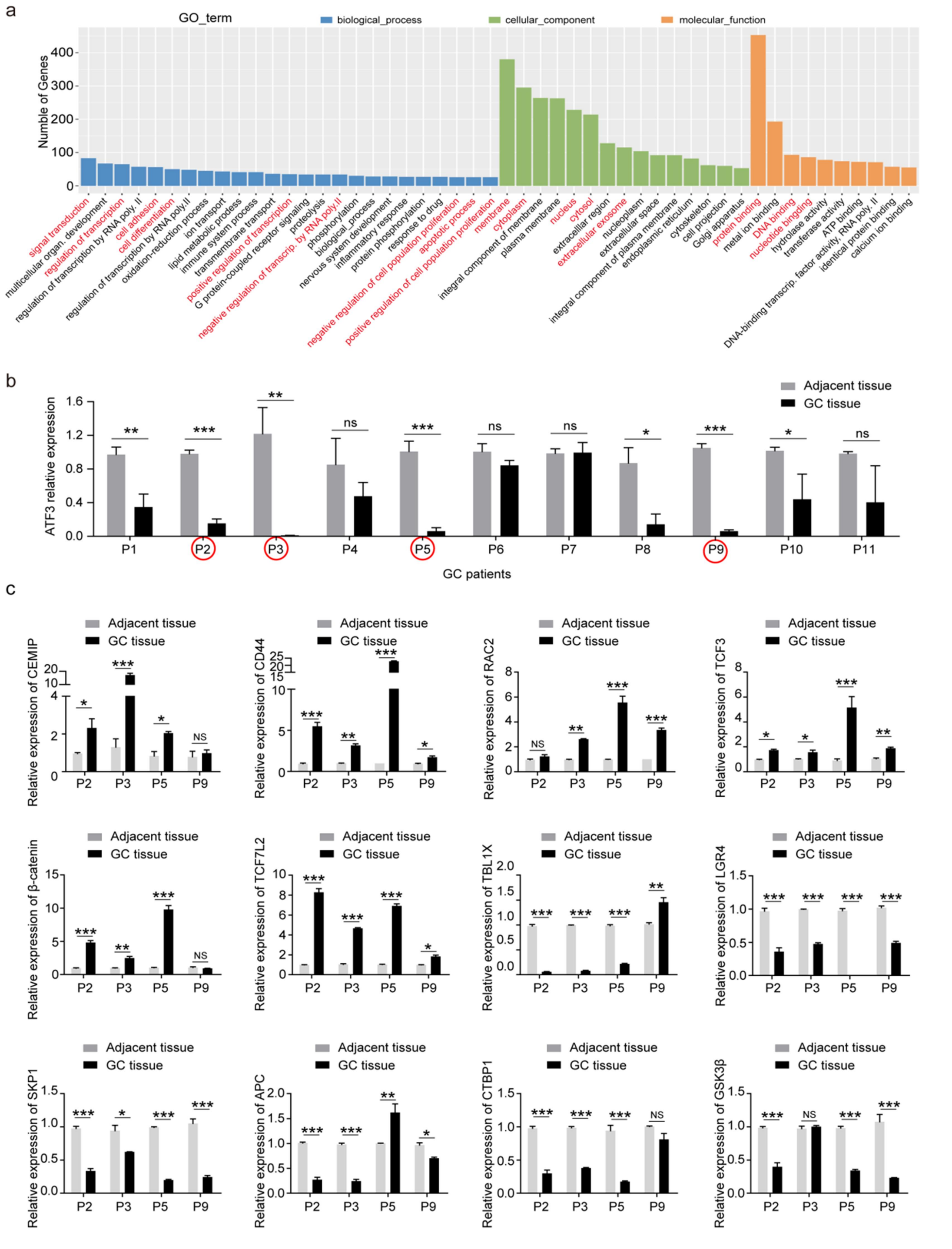

Supplementary Fig. 2

Supplementary Fig. 2 Transcriptome sequencing and qRT-PCR analysis. a Gene ontology (GO) enrichment analysis of the differentially expressed genes between ATF3-knockdown AGS cells and control cells. **b** qRT-PCR analysis of ATF3 expression in GC tissues. **c** Altered mRNA expression of genes involved in Wnt signal pathway were selectively confirmed by qRT-PCR in GC tissues with low- (ATF3^{low}) or high-ATF3 expression (ATF3^{high}). *$p < 0.05$; **$p < 0.01$, ***$p < 0.001$ and ns (not significant) by the Student t test compared with the control group.

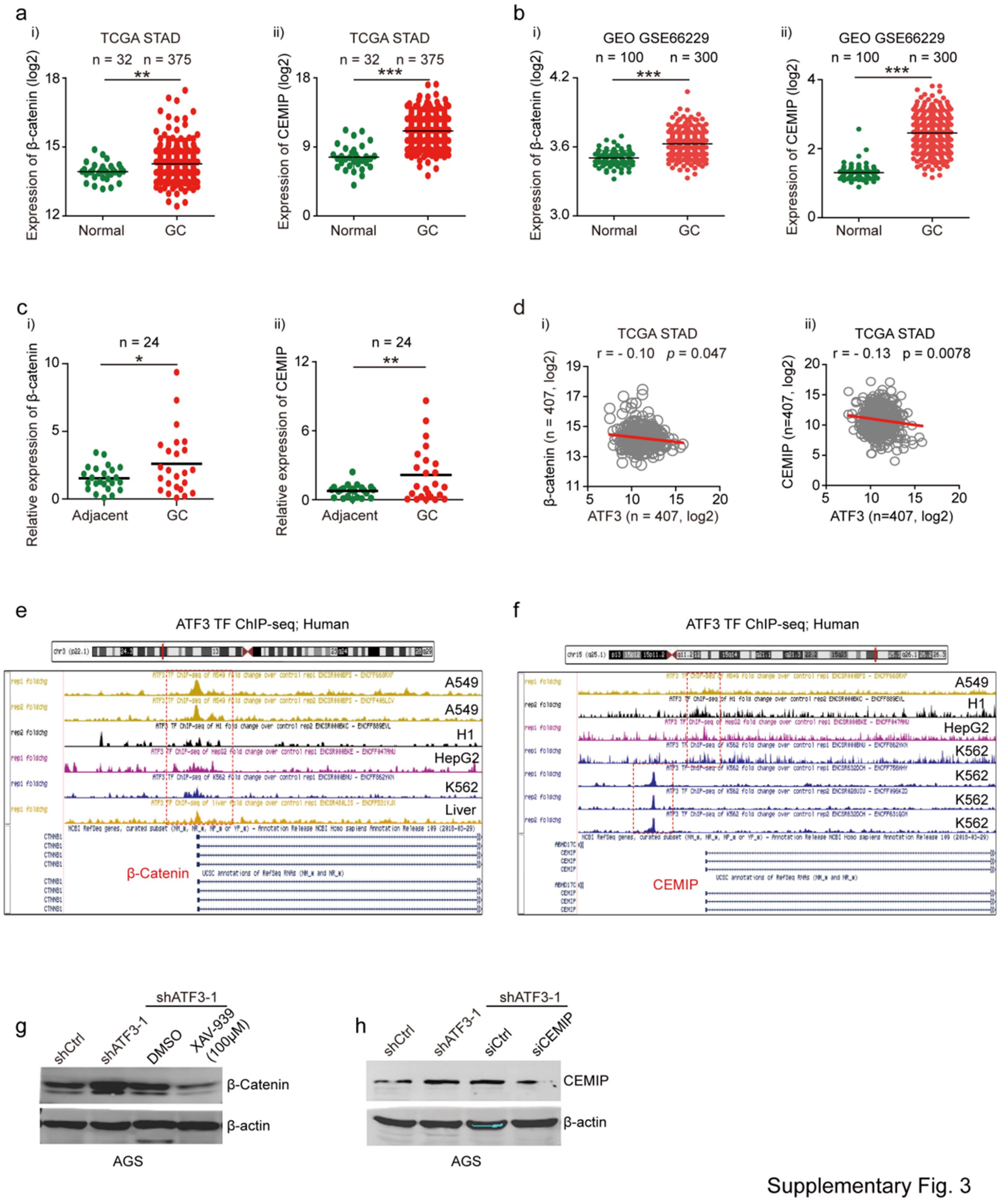

Supplementary Fig. 3 β-catenin and CEMIP were highly expressed in GC tissues and were the direct targets of ATF3. **a** The mRNA levels of β-catenin (i) and CEMIP (ii) were derived from the TCGA STAD samples measuring GC and normal tissues. **b** The mRNA levels of β-catenin (i) and CEMIP (ii) were derived from the GEO GSE66229 datasets measuring GC and normal tissues. **c** qRT-PCR analysis of β-catenin (i) and CEMIP expression levels (ii) in 24 pairs of GC and adjacent normal gastric tissues. **d** Correlations between the expression levels of ATF3 and β-catenin (i) or CEMIP (ii) in the TCGA STAD specimens. **e** and **f** Published ATF3 ChIP data was

retrieved from in Cistrome database (http://cistrome.org). **(e)** The binding of ATF3 to the promoter locus of β-catenin in A549 (Epithelium; Lung), H1 (Embryonic stem cell; Embryo), HepG2 (Epithelium; Liver), K562 (Erythroblast; Bone marrow) and Liver cells. **(f)** The binding of ATF3 to the promoter locus of CEMIP in A549, H1, HepG2 and K562 cells. **g** and **h** The β-catenin and CEMIP expression were detected by western blot in XAV-939 (β-catenin inhibitor, 100μM) **(g)** or CEMIP siRNA treated AGS cells **(h)** respectively. $*p < 0.05$, $**p < 0.01$ and $***p < 0.001$ by student's t-test compared with paired normal or control group.

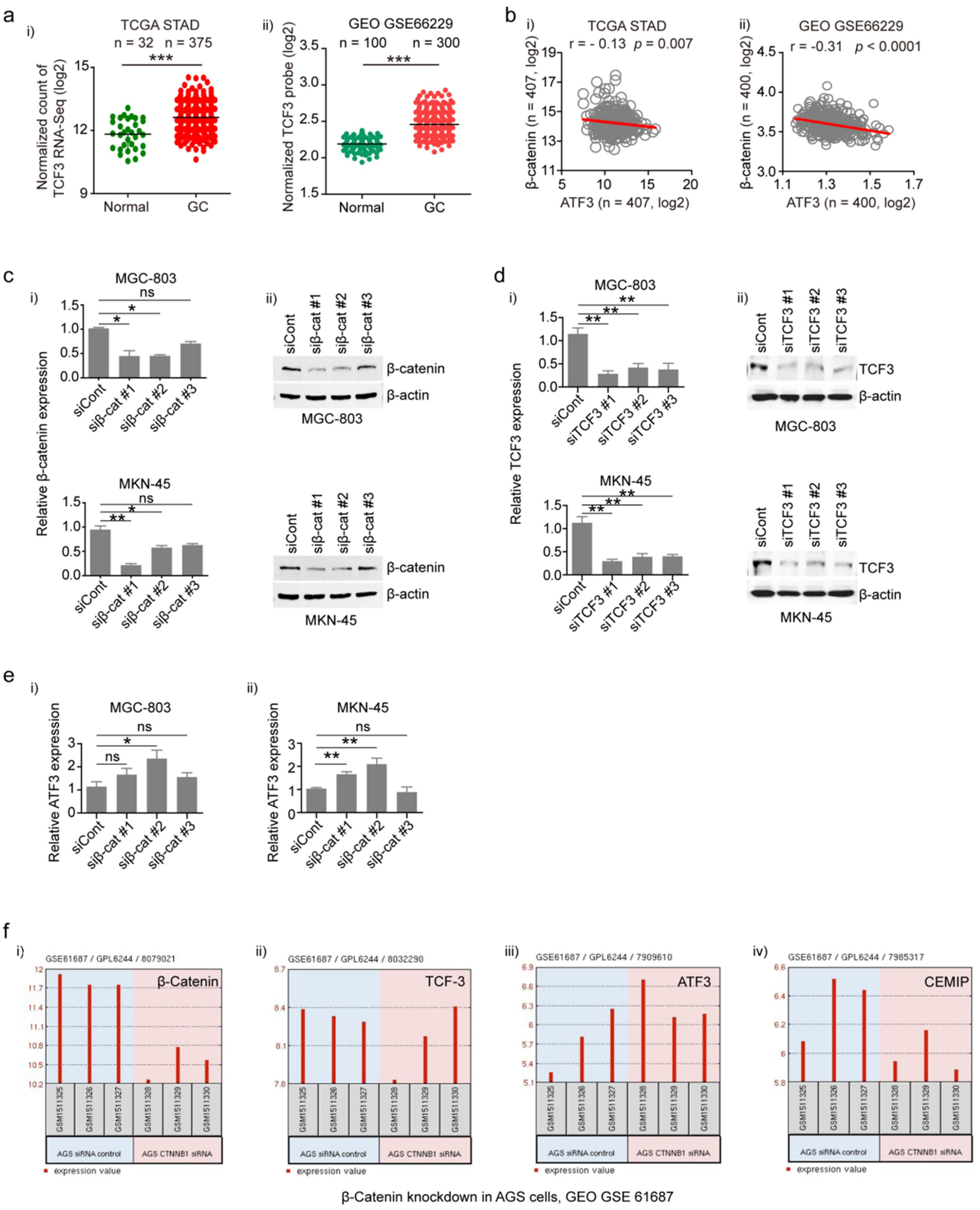

Supplementary Fig. 4

Supplementary Fig. 4 TCF3 was overexpressed in GC tissues and β-catenin / TCF3 axis repressed ATF3 expression in GC cells. a TCF3 expression data measured by RNA-seq or normalized probe were retrieved from TCGA STAD samples (i) and GEO GSE66229 datasets (ii) respectively. **b** Correlation between the mRNA levels of β-catenin and ATF3 in TCGA STAD specimens (i) and GEO GSE66229 dataset (ii). **c** qRT-PCR (i) and western blot analysis (ii) of β-catenin mRNA and protein levels in MGC-803 and MKN-45 cells transfected with β-catenin siRNA or negative control siRNA. **d** qRT-PCR (i) and western blot analysis (ii) of

TCF3 mRNA and protein levels in MGC-803 and MKN-45 cells transfected with TCF3 siRNA or negative control siRNA. **e** qRT-PCR analysis of ATF3 mRNA levels in MGC-803 (i) and MKN-45 cells (ii) transfected with β-catenin siRNA or negative control siRNA. **f** The expression levels of β-catenin (i), TCF3 (ii), ATF3 (iii), CEMIP (iv) in β-catenin knockdown AGS cells, the data retrieved from GEO GSE61687. *$p < 0.05$; **$p < 0.01$, ***$p < 0.001$ and ns (not significant) by the Student t test compared with the control group.

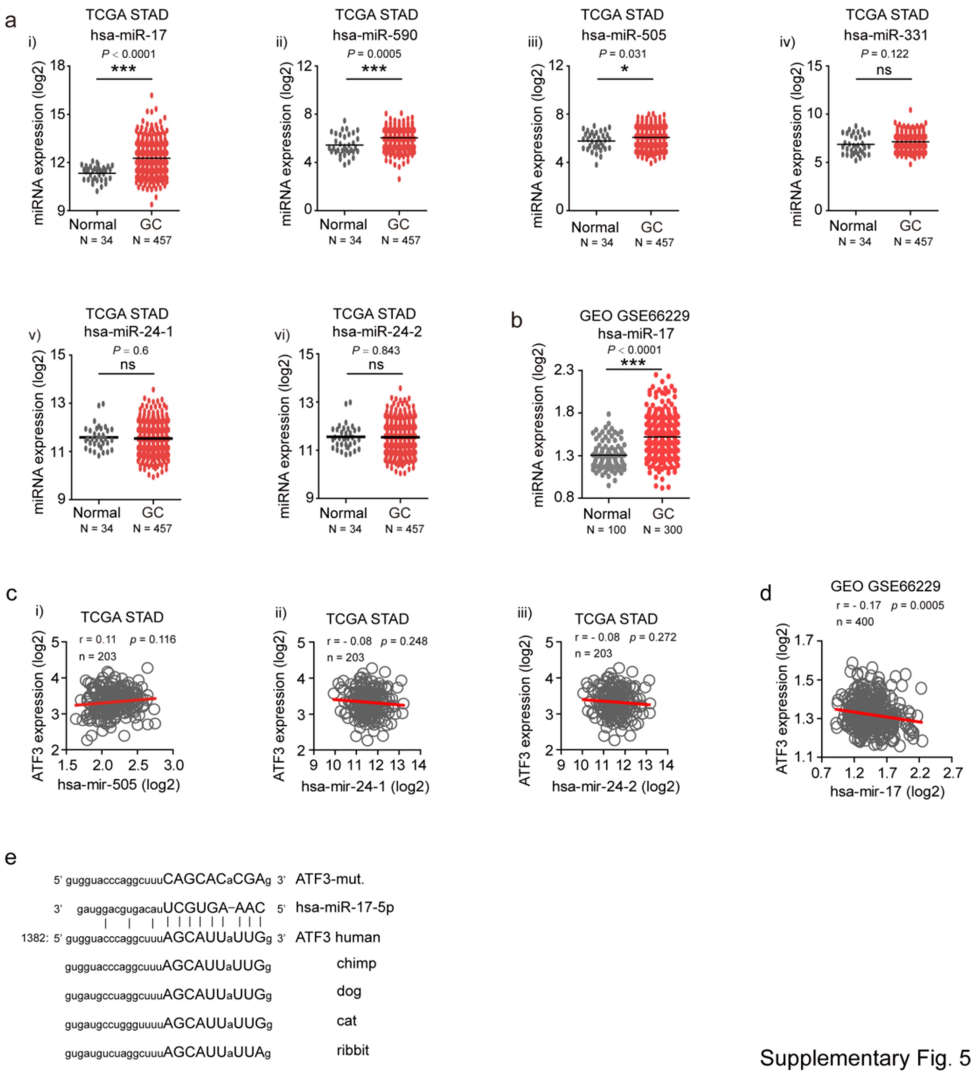

Supplementary Fig. 5

Supplementary Fig. 5 Analysis of ATF3-targeted miRNA expression and its correlation with ATF3 expression. a The miRNA levels of hsa-miR-17 (i), hsa-miR-590 (ii), hsa-miR-505 (iii), hsa-miR-331 (iv), hsa-miR-24-1 (v) and hsa-miR-24-2 (vi) were derived from the TCGA STAD datasets measuring GC tissues and normal tissues. **b** The miRNA expression level of hsa-miR-17 was derived from the GEO GSE66229 datasets measuring GC tissues and normal tissues. **c** Correlation between the expression levels of ATF3 and hsa-miR-505 (i), hsa-miR-24-1 (ii) or hsa-miR-24-2 (iii) in the TCGA STAD specimens. **d** Correlation between the expression levels of ATF3 and hsa-miR-17 retrieved from GEO GSE66229 datasets. **e** Sequence alignment of predicted miR-17-5p binding site in the 3'UTR of ATF3 of various mammals. *p < 0.05; ***p < 0.001 and ns (not significant) by the Student t test compared with the control group.

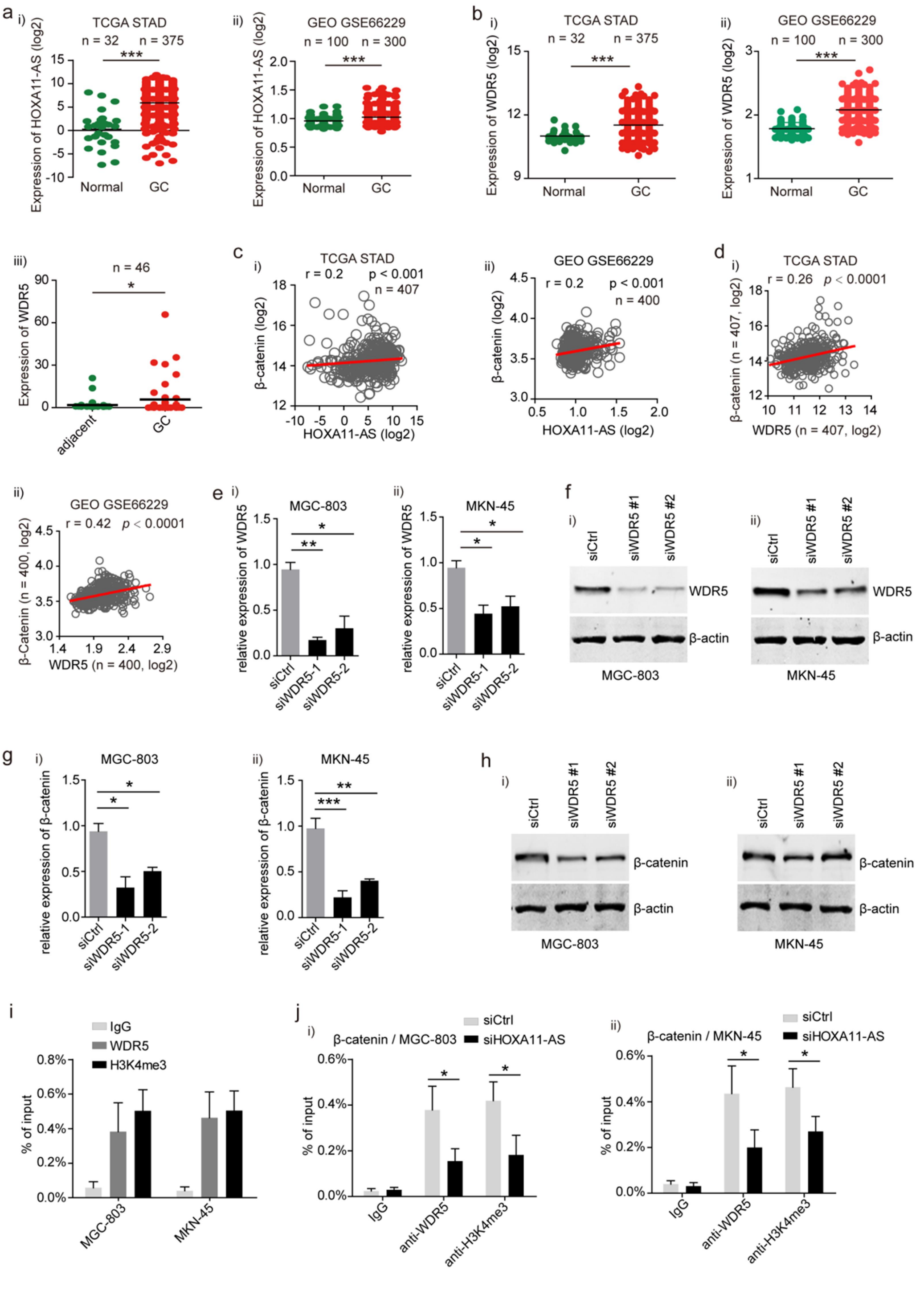

Supplementary Fig. 6

Supplementary Fig. 6 HOXA11-AS and WDR5 were overexpressed in GC tissues and promoted β-catenin expression. a HOXA11-AS expression data measured by RNA-seq or normalized probe were retrieved from TCGA STAD samples (i) and GEO database (ii) respectively. **b** WDR5 expression data were retrieved from TCGA STAD samples (i) and GEO datasets (ii) respectively, and were detected in our GC

samples (iii). **c** Correlation between the expression level of HOXA11-AS and β-catenin in the TCGA STAD specimens (i) and GEO datasets (ii) respectively. **d** Correlation between the expression levels of WDR5 and β-catenin in the TCGA STAD specimens (i) and GEO datasets (ii) respectively. **e and f** qRT-PCR and western blot analysis of WDR5 mRNA **(e)** and protein level **(f)** in MGC-803 (i) and MKN-45 cells (ii) transfected with WDR5 siRNA or negative control siRNA (shCtrl). **g** and **h** qRT-PCR and western blot analysis of β-catenin mRNA **(g)** and protein level **(h)** in MGC-803 (i) and MKN-45 cells (ii) transfected with WDR5 siRNA or control siRNA (shCtrl)**. i** ChIP-qPCR analysis of WDR5 and H3K4me3 occupancy in the β-catenin promoter in MGC-803 and MKN-45 cells**. j** ChIP-qPCR analysis of WDR5 and H3K4me3 occupancy in the β-catenin promoter in MGC-803 (i) and MKN-45 cells (ii) transfected with HOXA11-AS Smart Silencer (siHOXA11-AS) or control siRNA (siCtrl). **p* < 0.05, ***p* < 0.01 and ****p* < 0.001 by student's t-test compared with paired normal or control group.

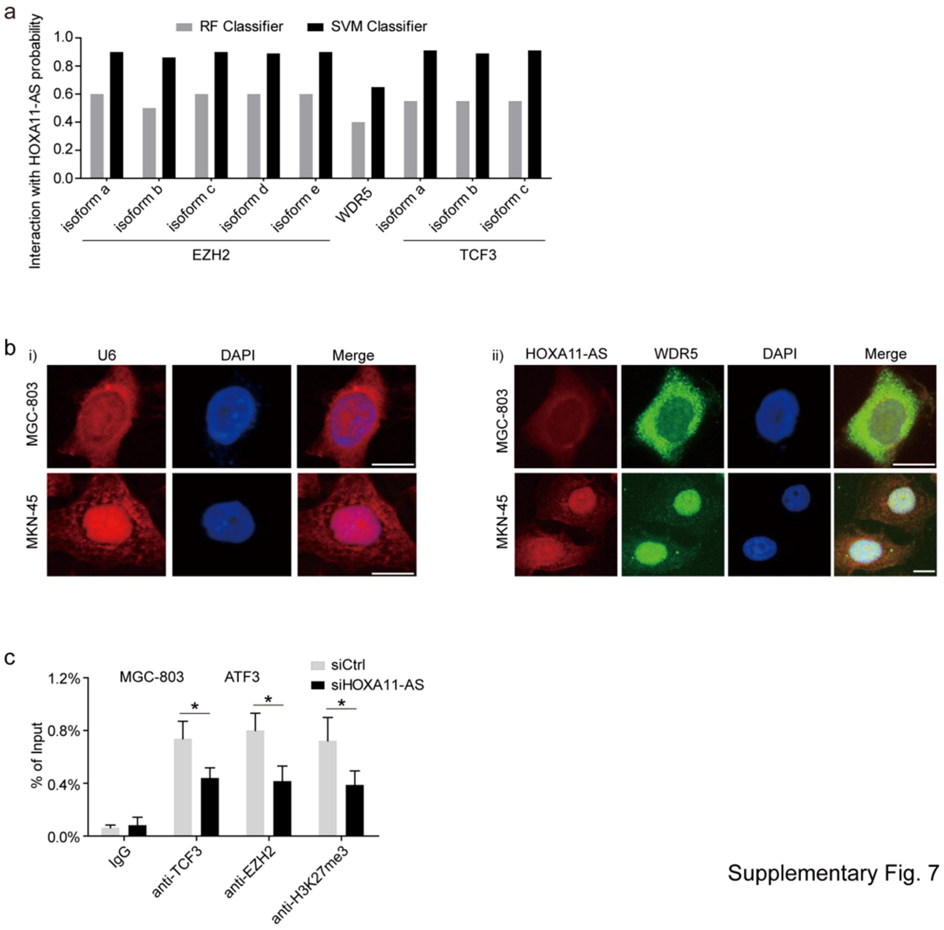

Supplementary Fig. 7

Supplementary Fig. 7 HOXA11-AS co-localized with TCF3 and WDR5 in GC cells. **a** Bioinformatics prediction of HOXA11-AS-interacting proteins. RPISeq predictions were based on random forest (RF) or support vector machine (SVM). **b** The human U6 snRNA was analyzed as a positive control by FISH in MGC-803 and MKN-45 cells (i). Red, U6; blue, DAPI. The co-location of HOXA11-AS and WDR5 was analyzed by co-staining of FISH and IF in MGC-803 and MKN-45 cells (ii). Red, HOXA11-AS; Green, WDR5 or TCF3; blue, DAPI. Scale bar: 10μm. **c** ChIP-qPCR analysis of TCF3, EZH2 and H3K27me3 occupancy in the ATF3 promoter in HOXA11-AS knockdown MGC-803 cells. * $p < 0.05$; ** $p < 0.01$ and ***$p < 0.001$ by student's t-test compared with the corresponding control group.

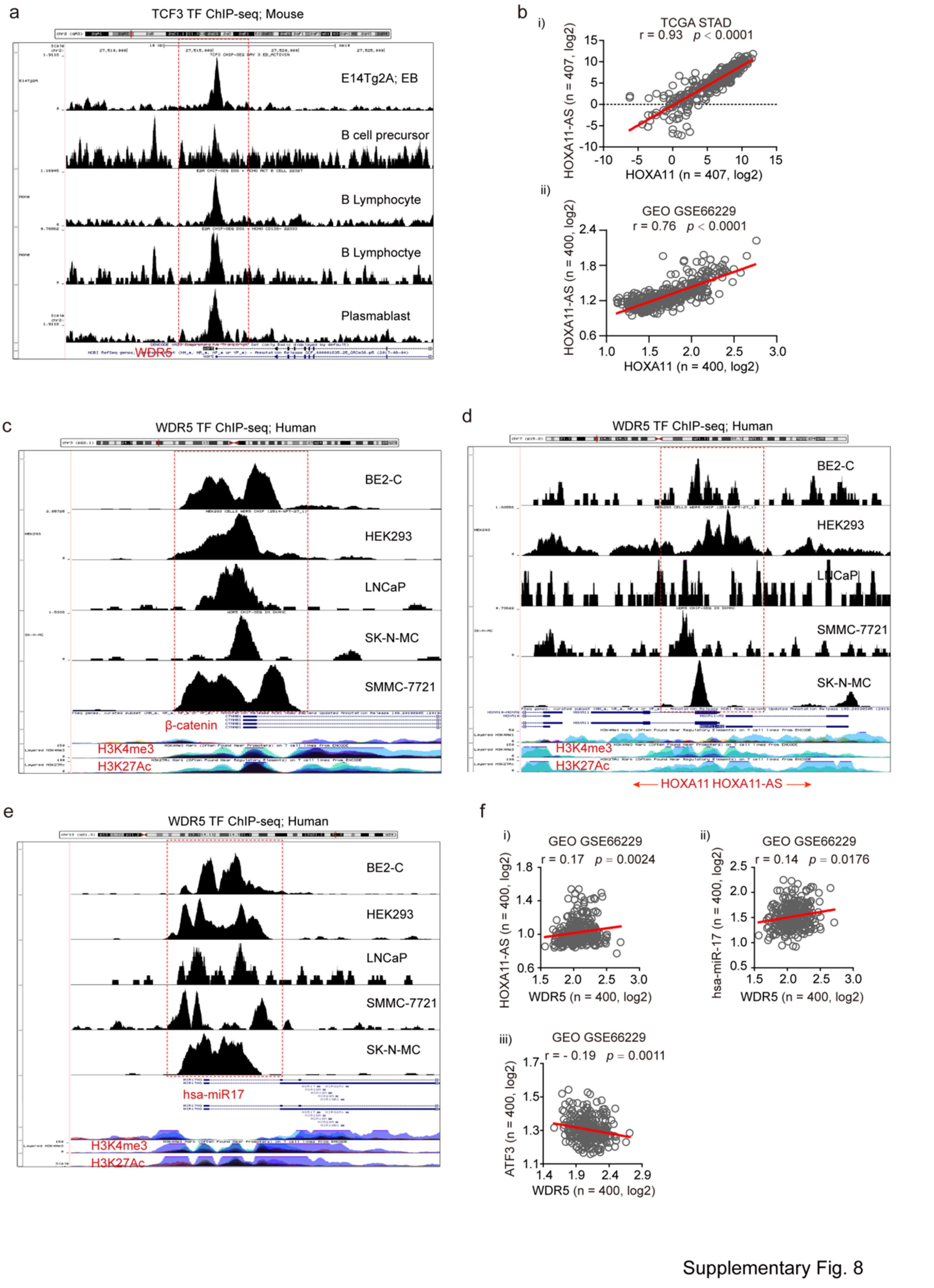

Supplementary Fig. 8 Abnormally elevated TCF3 in GC cells transactivated WDR5 and promoted the expression of β-catenin, HOXA11-AS and miR-17-5p. **a** Published mouse TCF3 ChIP data in Cistrome database (http://cistrome.org). The binding of mouse TCF3 to the promoter locus of mouse WDR5 in E14Tg2A, B cell precursor, B lymphocyte and plasmablast cells were analyzed. **b** Correlation between

the expression levels of HOXA11 and HOXA11-AS in GC specimens derived from the TCGA STAD samples (i) and GEO GSE66229 dataset (ii). **c, d and e** Published human WDR5 ChIP data was retrieved from the Cistrome database (http://cistrome.org). **(c)** The binding of WDR5 to the promoter locus of β-catenin in BE2-C (Neruoblast; Brain), HEK293 (Epithelium; Embryonic kidney), LNCap (Epithelium; Prostate), SK-N-MC (Brain) and SMMC-7721 (HCC) cells were analyzed. **(d)** The binding of WDR5 to the promoter locus of HOXA11-AS in BE2-C, HEK293, LNCap, SMMC-7721 and SK-N-MC cells were analyzed. **(e)** The binding of WDR5 to the promoter locus of hsa-miR-17 in BE2-C, HEK293, LNCap, SMMC-7721 and SK-N-MC cells. **f** Correlations between the expression levels of WDR5 and HOXA11-AS (i), hsa-miR-17 (ii) or ATF3 (iii) in 400 pairs of GC specimens derived from the GEO GSE66229 datasets.
